# STRIPAK-associated greenbeard proteins DOC1 and DOC2 regulate MAK2 signaling and sexual development in *Sordaria macrospora*

**DOI:** 10.1371/journal.pbio.3003969

**Published:** 2026-08-27

**Authors:** Lucas S. Hollstein, Kerstin Schmitt, Lucas Well, André Fleißner, Oliver Valerius, Stefanie Pöggeler

**Affiliations:** 1 Department of Genetics of Eukaryotic Microorganisms, Institute of Microbiology and Genetics, University of Göttingen, Göttingen, Germany; 2 Department of Molecular Microbiology and Genetics, Institute of Microbiology and Genetics, University of Göttingen, Göttingen, Germany; 3 Service Unit LC–MS Protein Analytics, Göttingen Center for Molecular Biosciences (GZMB), University of Göttingen, Göttingen, Germany; 4 Department of Fungal Genetics, Institute of Genetics, Technische Universität Braunschweig, Braunschweig, Germany; Duke University Medical Center, UNITED STATES OF AMERICA

## Abstract

Hyphal fusion and sexual development in filamentous fungi rely on coordinated signaling of numerous conserved nodes such as the striatin-interacting phosphatase and kinase (STRIPAK) complex or the pheromone response (PR) MAP kinase cascade (MIK2, MEK2, MAK2, HAM5). Here, we used the homothallic ascomycete *Sordaria macrospora* (Sm) to screen for putative protein interactors of the SmSTRIPAK complex. Using the STRIPAK complex interactor 1 (SCI1) subunit of the complex as bait, we enriched and identified canonical SmSTRIPAK components and a determinant of communication (DOC) protein. The DOC proteins were previously described in the closely related and heterothallic species *Neurospora crassa*, functioning in allorecognition of germlings and hyphal fusions. We generated ΔSmdoc1, ΔSmdoc2 single-deletion strains and the double deletion mutant ΔSmdoc1ΔSmdoc2 in *S. macrospora*. Deletion phenotypes were paradoxical: single knockouts (ΔSmdoc1 or ΔSmdoc2) were nearly sterile, and sexual development was impaired, yet the double mutant (ΔSmdoc1ΔSmdoc2) exhibited wild-type fertility and development, demonstrating non-redundant and mutually antagonistic roles. Using gene tagging at the native locus, we performed TurboID-based proximity mapping with SmDOC1 and SmDOC2 as bait proteins. This proximity mapping demonstrated close ties of SmDOC1/2 to components of the PR MAP kinase pathway and revealed mutual SmDOC1 – SmDOC2 proximity. Yeast two-hybrid experiments with SmDOC1 confirmed the direct interaction with the MAP kinases MEK2 and MAK2. Fluorescence microscopy revealed that SmDOC1-TagRFP-T localized to structures near septal pores. Our results demonstrate that the DOC system is not restricted to heterothallic *N. crassa* but also plays an essential role in the development of fruiting bodies in the homothallic fungus *S. macrospora*. These findings suggest the DOC1/2 proteins as a novel system that integrates STRIPAK and PR pathways, providing a possible mechanistic explanation for their non-additive deletion strain phenotypes.

## Abstract 2

How can signaling networks coordinate hyphal fusion and sexual development in fungi? This study uses STRIPAK proximity mapping to identify DOC1 and DOC2 as antagonistic links between STRIPAK and MAK2 signaling that control fertility in *Sordaria macrospora*.

## Introduction

In this study, we used the filamentous ascomycete *Sordaria macrospora* to study the molecular mechanisms of multicellular development during fruiting body formation [1]. This homothallic (self-fertile) fungus undergoes sexual development and production of meiotic spores (ascospores) without the need of a mating partner within seven days under laboratory conditions. The sexual development starts with the formation of ascogonial coils, the female gametangia, which are enveloped by sterile hyphae, giving rise to the spherical protoperithecia (pre-fruiting bodies). After melanization of the perithecial wall, differentiation continues and the characteristic flask-shaped perithecia enclosing asci are formed after karyogamy and meiosis. Once mature perithecia are developed, they eject haploid ascospores [2]. Filamentous fungi have evolved complex and conserved regulatory circuits to orchestrate growth and differentiation processes, such as hyphal fusion, sexual reproduction and multicellular development. These circuits function as molecular information processing switches by coordinating the appropriate cellular responses depending on a variety of environmental and developmental factors. Two examples of such regulatory networks are the striatin-interacting phosphatase and kinase (STRIPAK) complex and the pheromone response (PR) pathway. Both signaling pathways are conserved in unicellular and filamentous fungi and act as crucial elements to balance developmental programs in eukaryotes. The PR signaling pathway consists of the three-tiered mitogen-activated protein (MAP) kinase cascade MIK2 (MAPKKK), MEK2 (MAPKK), the terminal kinase MAK2 (MAPK) and the scaffolding protein HAM5 in filamentous ascomycetes such as the self-sterile (heterothallic) *Neurospora crassa* and *S. macrospora* [3–8]. The PR signaling complex regulates sexual development, hyphal fusion and melanin-dependent ascospore germination [8]. In *N. crassa*, components of the PR pathway exhibit a characteristic oscillatory dynamic during the chemotropic communication among two cells prior to cell fusion [9]. Components of the PR pathway and the SOFT (SO) scaffolding protein of the cell wall integrity MAPK (MAK1) kinase pathway are recruited to opposing cell tips in a coordinated out-of-phase manner. While the PR signaling complex accumulates at the plasma membrane of one of the cells, the opposing cell exhibits antiphase accumulation of SO. The roles of the cells are switched after each wave, which lasts for three to six minutes. This cycle of oscillating spatiotemporal coordination is repeated until physical contact of the two cells is established [3,10]. This oscillatory communication system of *N. crassa* is accompanied by the determinant of communication (doc) system, which was discovered by bulk segregant analysis followed by whole-genome sequencing using a wild *N. crassa* population [11]. The highly polymorphic “greenbeard genes” *doc-1*, *doc-2*, and *doc-3* were shown to regulate long-distance self/non-self recognition by defining five distinct communication groups (CGs) within *N. crassa* populations. The concept of “greenbeard genes” was advanced as a theoretical proposition in the form of a thought experiment, intended to provide an explanation for the proposition that altruism may be a self-serving strategy from the standpoint of genes [12,13]. Isolates with the same CG affiliation show higher communication frequencies, whereas isolates from different CGs show lower communication and cell fusion frequencies. The *doc* genes regulate the chemotropic interactions of isolates depending on the set of *doc* alleles harbored by the two individuals (allorecognition) and prevent somatic cell fusion of genetically dissimilar cells. Fluorescence microscopy of DOC1 tagged with fluorescent protein demonstrated a co-oscillation with MAK2 to the cell tips [11].

Mounting evidence suggests bidirectional crosstalk between the STRIPAK complex and the PR pathway, with STRIPAK complex components acting as upstream regulators and downstream targets of MAP kinase cascades [14,15]. In contrast to the kinase-driven mode of action in the PR pathway, the STRIPAK complex is mainly characterized by its phosphatase activity. The STRIPAK complex is a multisubunit phosphatase assembly that has been extensively characterized in fungi, where it regulates fruiting body formation, cell fusion, vegetative and sexual development, pathogenicity, secondary metabolism, and virulence of plant pathogens [14,16–21].

In *S. macrospora*, the SmSTRIPAK complex has been investigated for over 20 years. It consists of the striatin homolog PRO11 (homolog of human striatin), the scaffolding subunit SmPP2AA (PP2AA), the catalytic subunit SmPP2Ac1 (PP2AC), the developmental protein PRO22 (STRIP1/2), the kinase activator SmMOB3 (phocein), the small coiled-coil protein STRIPAK complex interactor 1 (SCI1, homolog of human SIKE1) and sarcolemma membrane-associated protein (SLMAP) PRO45 [22–27]. Furthermore, the *S. macrospora* homologs of the human kinases MST1/2 (SmKIN3) and MST3/4 (SmKIN24) were shown to associate with the assembly [28,29]. The deletion of SmSTRIPAK components often results in impaired vegetative growth and an arrest of sexual development at early stages, leading to sterility [30]. According to a cryo-EM structure of the human STRIPAK complex, the SmSTRIPAK complex stoichiometry consists of a PRO11 homotetramer, which functions as the main backbone of the multiprotein assembly [31]. It is accompanied by one copy of each of the other components: SmPP2AA, SmPP2Ac1, PRO22, SmMOB3, SCI1 and PRO45. The STRIPAK complex functions as a signaling hub by controlling dephosphorylation of numerous target proteins, thereby regulating diverse cellular processes. Correspondingly, phosphoproteomic analyses of SmSTRIPAK null mutants identified over 100 proteins with SmSTRIPAK-dependent phosphorylation sites [15,32].

In our study, Biotin Identification (BioID) proximity labeling combined with mass spectrometry was applied to capture the protein networks of the SmSTRIPAK complex within the microenvironment of the subunit SCI1. Using multiple control setups, we significantly enriched and identified the *S. macrospora* homolog of *N. crassa*’s DOC-2 as a putative interactor of the SmSTRIPAK complex. We then investigated and characterized the *Smdoc1* and *Smdoc2* genes of the homothallic species *S. macrospora* and demonstrated that the function of the DOC system is not restricted to the heterothallic *N. crassa*, but extends to homothallic *S. macrospora*. The single knockout strains ΔSmdoc1 and ΔSmdoc2 are severely impaired in sexual development, while the double knockout strain ΔSmdoc1ΔSmdoc2 displays wild-type-like phenotypes. For further analysis, *Smdoc1/2* fusions with the TurboID ligase and fluorescent tags were constructed and integrated at their respective *Smdoc* loci. Proximity labeling with mass spectrometry and yeast two-hybrid (Y2H) interaction studies demonstrated close ties and direct interactions of the SmDOC system with the PR MAP kinase signaling module. Our findings propose a novel role of the *doc* genes in sexual reproduction and a so far uncharacterized negative regulatory network that might link the SmSTRIPAK signaling with the PR MAP kinase pathway via the SmDOC1/2 system.

## Results

### SCI1-BioID experiments identified SmDOC2 as a putative SmSTRIPAK interactor

**Fig 1:**
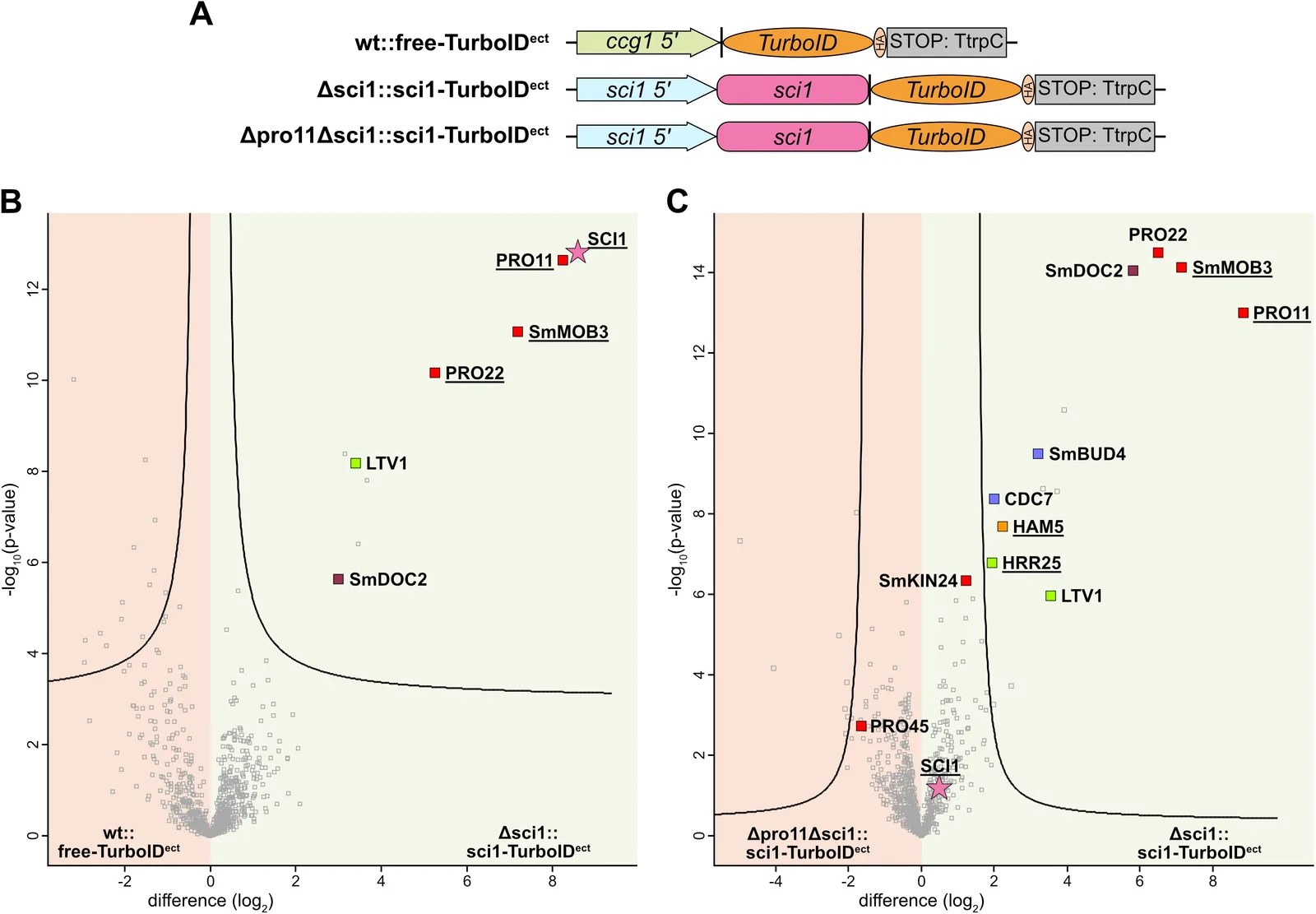
Volcano plot analysis of the SCI1-BioID experiments. **(A)** Constructs for SCI1-BioID experiments using either the constitutive *clock-controlled gene 1* (*ccg1*) promoter from *N. crassa* or the native *sci1* promoter for expression. *TurboID* exhibits an N-terminal *linker* (2× GGGGS) and a C-terminal *3x HA* tag. **(B, C)** Volcano plot analysis of SCI1-BioID experiments using a free TurboID control **(B)** and a SmSTRIPAK-dependent control **(C)**, expressing *sci1-TurboID* in the Δpro11 background. The graphs plot the difference in Label-Free Quantification (LFQ) intensities (log_2_ transformed) of the control strain on the left (red) and Δsci1::sci1-TurboID^ect^ on the right (green). The − log_10_(*p*-value) is plotted on the y-axis. Significantly enriched proteins are separated from non-significant proteins by the plotted curve. The bait protein SCI1 is marked with a pink star, other marked proteins include components of the SmSTRIPAK complex (red), components of the septation initiation network (SIN) in blue, components of the pheromone response pathway (orange), the casein kinase HRR25 and LTV1 (light green) and SmDOC2 (magenta). Proteins that were identified with biotin site information are underlined. **(B)** Eight biological replicates of each, wt::free-TurboID^ect^ and Δsci1::sci1-TurboID^ect^ were grown in liquid BMM medium for three days at 27 °C under constant light. Statistical parameters: FDR = 0.01; s0 = 0.1. The data underlying this figure can be found in S1 and S2 Data. **(C)** Eight biological replicates of each Δpro11Δsci1::sci1-TurboID^ect^ and Δsci1::sci1-TurboID^ect^ were grown in liquid BMM medium for four and three days, respectively, at 27 °C under constant light. Statistical parameters: FDR = 0.01; s0 = 2. The data underlying this figure can be found in S3 and S4 Data.

To gain a deeper understanding of the SmSTRIPAK complex, we performed an unbiased screening for putative protein-protein interactors by applying the BioID method in combination with mass spectrometry. The BioID approach relies on a promiscuous biotin ligase, here TurboID, which is fused to a protein of interest [33,34]. This fusion construct is expressed in the organism and upon the availability of biotin, adjacent proteins get covalently labeled with biotin in vivo (S1 Fig). This covalent labeling enables protein extraction and enrichment of biotinylated proteins under denaturing conditions. This supports efficient solubilization of membrane-bound, aggregated or poor resolvability and reduces artificial association of proteins with the bait during or after cell lysis. The biotinylated proteins are selectively enriched from denatured whole cell lysates by affinity purification and are subsequently identified by mass spectrometry [35,36]. Proteins of the bait-ligase fusion-harboring strain are quantified relative to a control strain (e.g., expressing an unfused biotin ligase). In this study, we used the *sci1* subunit-encoding gene of the SmSTRIPAK complex as bait and fused it to the *TurboID* biotin ligase for the proximity labeling [37]. The SCI1 subunit has previously to be a component of the SmSTRIPAK complex. Its relatively small molecular weight of ~33 kDa allows simple handling during cloning and protein experiments [25]. In this new SCI1-BioID experiment, significantly enriched proteins were determined by relative quantification with a control strain, which expresses an unfused TurboID ligase under the control of the constitutive *clock-controlled gene 1* (*ccg1*) promoter from *N. crassa* [38,39]. The *S. macrospora* strains were cultivated in liquid medium, cells were lysed in the presence of SDS as detergent, and biotinylated proteins were enriched from the protein crude extract using Strep-Tactin Sepharose. The captured proteins were digested with trypsin, and the resulting peptides were analyzed by LC–MS as described previously [40]. This SCI1-BioID experiment identified the already known SmSTRIPAK subunits PRO11, SmMOB3 and PRO22, thereby validating the proximity labeling approach (Fig 1A and 1B; S1 Data). In addition to the known SmSTRIPAK components, the BioID experiment significantly enriched the Low temperature viability 1 (LTV1) protein and a protein annotated as SMAC_06902. Sequence analysis via BLASTP with SMAC_06902 as query identified the *N. crassa* greenbeard protein DOC-2 (NCU07192) with an amino acid identity of 86% (100% query coverage) and an *e*-value of 0.0 as closest hit. Thus, we named the protein encoded by *SMAC_06902* SmDOC2. Using the amino acid sequence of the neighboring gene of *Smdoc2*, SMAC_06903 as query for BLASTP, we identified *N. crassa* DOC-1 (NCU07191) as the closest hit with 91% sequence identity (100% query coverage); hence, we named the protein SmDOC1. Amino acid alignments of SmDOC1 and SmDOC2 with DOC-1 and DOC-2 from *N. crassa* are shown in S2A and S2B Fig. The loci of the *doc* genes of *S. macrospora* and the common *N. crassa* lab strain FGSC 2,489 are syntenic (S2C Fig).

For a more refined enrichment quantification of proteins in SCI1-BioID experiments, we constructed a novel control strain by crossing the SCI1-TurboID strain with the SmSTRIPAK mutant Δpro11. PRO11 is the homolog of vertebrate striatin and functions as the main scaffold protein of the SmSTRIPAK complex. The Δpro11 deletion strain exhibits a sterile phenotype and only produces rudimentary ascogonia [23]. The outcome of the cross was analyzed by PCR and Southern blot experiments to properly verify the double deletion Δpro11Δsci1 (S3 Fig). This experimental setup aims to identify the striatin (PRO11)-dependent (and in a broader context the SmSTRIPAK-dependent) proxiome of SCI1-TurboID. Consistent with the sterile phenotype of Δpro11, the newly generated Δpro11Δsci1::sci1-TurboID^ect^ strain produced no fruiting bodies, even after prolonged incubation (S4 Fig). The BioID experiment using the Δsci1::sci1-TurboID^ect^ strain in combination with the Δpro11Δsci1::sci1-TurboID^ect^ control strain showed pronounced enrichment of the SmSTRIPAK components PRO22, PRO11 and SmMOB3 (Fig 1C). The SmSTRIPAK-associated kinase SmKIN24 was slightly enriched but did not pass the significance threshold. Moreover, PRO45 (SLMAP homolog), a direct interactor of SCI1, was found in the SCI1 proximity with two biotin sites independent of the Δpro11 deletion strain background. PRO45 was shown to directly interact with SCI1 in Y2H experiments [25]. Contrary to the experimental BioID setup using the free TurboID control, the bait protein SCI1-TurboID is not among the significantly enriched proteins, since it is expressed equally by both strains (Fig 1A). However, in both experimental setups, SmDOC2 was significantly enriched. Upon deletion of the gene encoding the SmSTRIPAK scaffold PRO11, SmDOC2 was identified with an extremely high enrichment value of log_2_(difference) = 5.8, which corresponds to a 55-fold increase in intensity. Only the known SmSTRIPAK components PRO11, SmMOB3 and PRO22 report higher enrichment values or statistical significance than SmDOC2 in this experiment (Fig 1C). Among the other significantly enriched proteins are components of the septation initiation network (SIN), namely the proteins STE kinase CDC7 and the downstream landmark protein SmBUD4. The SIN has previously been described in connection with the STRIPAK complex [41–43]. The proximity labeling data also revealed significant enrichment of the scaffolding protein HAM5, which acts in the MAP kinase cascade of the PR pathway [3]. In this experiment, HAM5 was identified with two peptides containing biotinylated lysine residues in seven out of the eight replicates of Δsci1::SCI-TurboID^ect^. Other notable hits among the significantly enriched proteins include the casein kinase HO and radiation repair 25 (HRR25) and the ribosome assembly factor LTV1.

Due to the vastly different developmental potential of the strains used in the SmSTRIPAK-dependent experimental setup, comparing a fertile with a sterile strain (Δsci1::sci1-TurboID^ect^ and Δpro11Δsci1::sci1-TurboID^ect^), we performed mass spectrometry analysis of the crude protein extracts prior to biotin affinity purification. This enabled us to identify false positive hits, that emerged from differences in global protein abundances, rather than changes in the proxiome of the SCI1-TurboID labeling complex. Consistent with the literature, this proteome dataset showed downregulation of proteins involved in melanin biosynthesis in the Δpro11 background, which has been observed in RNA-seq experiments [44]. Most importantly, this analysis showed that the protein abundances of SmDOC1 and SmDOC2 are not affected by the deletion of *pro11* in the Δpro11Δsci1::SCI-TurboID^ect^ BioID control strain (S5 Fig; S5 and S6 Data). Hence, the significant enrichment of SmDOC2 in the BioID experiment probing the SmSTRIPAK-dependent environment of SCI1 is based on the proximity of SCI1 and SmDOC2, rather than proteomic downregulation in the Δpro11 deletion strain background.

### Deletion of *Smdoc1* and *Smdoc2* impairs sexual development

Based on the experimental SCI1-BioID data, SmDOC2 represents the most compelling candidate for further investigation among the identified proteins for several reasons: SmDOC2 demonstrated significant enrichment against the free TurboID control. Additionally, SmDOC2 was enriched in a SmSTRIPAK-dependent manner, since its abundance was drastically reduced in the Δpro11 deletion strain background. Only the SmSTRIPAK components PRO11, SmMOB3, and PRO22 showed higher enrichment values, placing SmDOC2 in an outstanding category of potential interactors. Unlike the other candidates described above, SmDOC proteins have been identified across multiple proteomic approaches using SmSTRIPAK components as bait [24,45]. Another argument for prioritizing SmDOC2 lies in the functional overlap between DOC proteins and the SmSTRIPAK complex. Both regulatory systems control hyphal fusion events, a fundamental process in fungal development. The DOC proteins of *N. crassa* mediate pre-contact allorecognition between germlings and control this first checkpoint during somatic cell fusion [11]. Similarly, the SmSTRIPAK complex signaling is essential for hyphal fusion and multicellular development in filamentous fungi [21]. This functional convergence might place the SmDOC proteins as a novel SmSTRIPAK-interacting regulatory node connecting these two critical signaling pathways. Therefore, we decided to functionally characterize *Smdoc1* and *Smdoc2* in *S. macrospora.*

**Fig 2:**
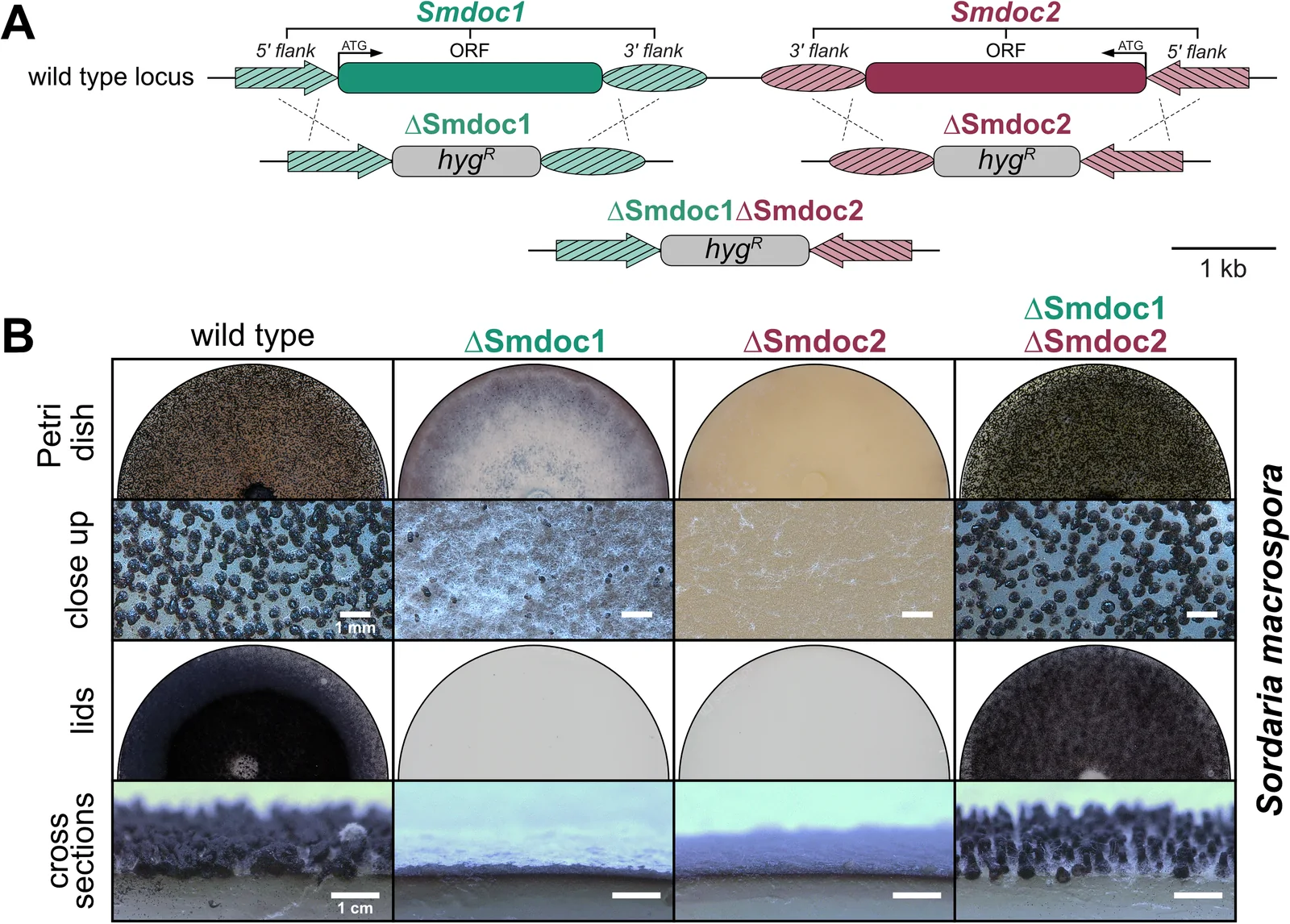
The *Smdoc* genes control sexual development in *S. macrospora.* **(A)** Genetic map of the *Smdoc* locus in the homothallic ascomycete *S. macrospora*. The single knockouts ΔSmdoc1 and ΔSmdoc2 as well as the double knockout ΔSmdoc1ΔSmdoc2 were generated via homologous recombination. The knockout cassette (*hyg*^*R*^) confers resistance to the hygromycin antibiotic. 1 kb flanking regions were used to target the cassette to the respective locus. **(B)** The single spore isolates were grown at 27 °C on solid *Sordaria* Westergaard’s (SWG) fructification medium. Pictures of the Petri dishes, the close-ups, the lids and the cross sections were taken after 14 days of incubation. Once the black ascospores are fully matured inside the fruiting bodies, they are forcefully ejected towards the light source and stick to the lids of the Petri dishes, thereby staining them black. *hyg*^*R*^, hygromycin resistance cassette expressing the *hygromycin B phosphotransferase* gene from *E. coli* under the control of the constitutive *trpC* promoter from *A. nidulans* [46].

For functional characterization of *Smdoc1/2* in *S. macrospora*, single deletion strains of ΔSmdoc1, ΔSmdoc2 and the double deletion strain ΔSmdoc1ΔSmdoc2 were generated (Fig 2A) and verified by PCR and Southern hybridization experiments (S6–S8 Figs). When grown on agar plates under standard conditions, the ΔSmdoc1 and ΔSmdoc2 single knockouts exhibit severe impairments in fruiting body formation and only a few fruiting bodies are produced, while most sexual structures did not progress past the stage of protoperithecia (Fig 2B). Ascospores harvested from the sparse fruiting bodies were viable. Additionally, the single knockouts ΔSmdoc1 and ΔSmdoc2 exhibit an unusually dense mycelium layer on top of the agar, which was cut-resistant when cutting agar pieces with a lancet. Hyphal fusion events in the vegetative mycelium were observed in both single knockout strains (S9 Fig). Vegetative growth analyses were performed in 30 cm long race tubes filled with synthetic SWG fructification medium. In this experiment, we noticed a delay in colony establishment of ΔSmdoc1 and ΔSmdoc2. The single knockouts took up to two days post-inoculation until the growth front of the mycelium was visible to the eye. Accordingly, this slower colony establishment of ΔSmdoc1 and ΔSmdoc2 resulted in shorter total growth when measurements were taken after 3 days. However, once the colony establishment stage was passed, the single knockouts reached wild-type-like growth rates per day (~25 to 30 mm per 24 h). The double knockout ΔSmdoc1ΔSmdoc2 did not show any delay in colony establishment or vegetative growth rate and was indistinguishable from the wild type in this experiment (S10 Fig; S7 and S8 Data). Surprisingly, the ΔSmdoc1ΔSmdoc2 double knockout produced fruiting bodies in quantities similar to the wild type (Fig 2B). The double knockout does not display any abnormal growth phenotypes. The single deletion strains ΔSmdoc1 and ΔSmdoc2 were complemented by ectopic integration of constructs harboring the *Smdoc1* or *Smdoc2* ORF flanked by 1 kb upstream and downstream regions. The fruiting body formation of ΔSmdoc2 reverted to wild-type levels upon reintroduction of the *Smdoc2* gene. Similarly, the ΔSmdoc1::Smdoc1^ect^ strain regained the ability to produce fruiting bodies, however, the density of fruiting bodies was increased when compared to the wild type (S11–S13 Figs and S9 Data).

**Fig 3:**
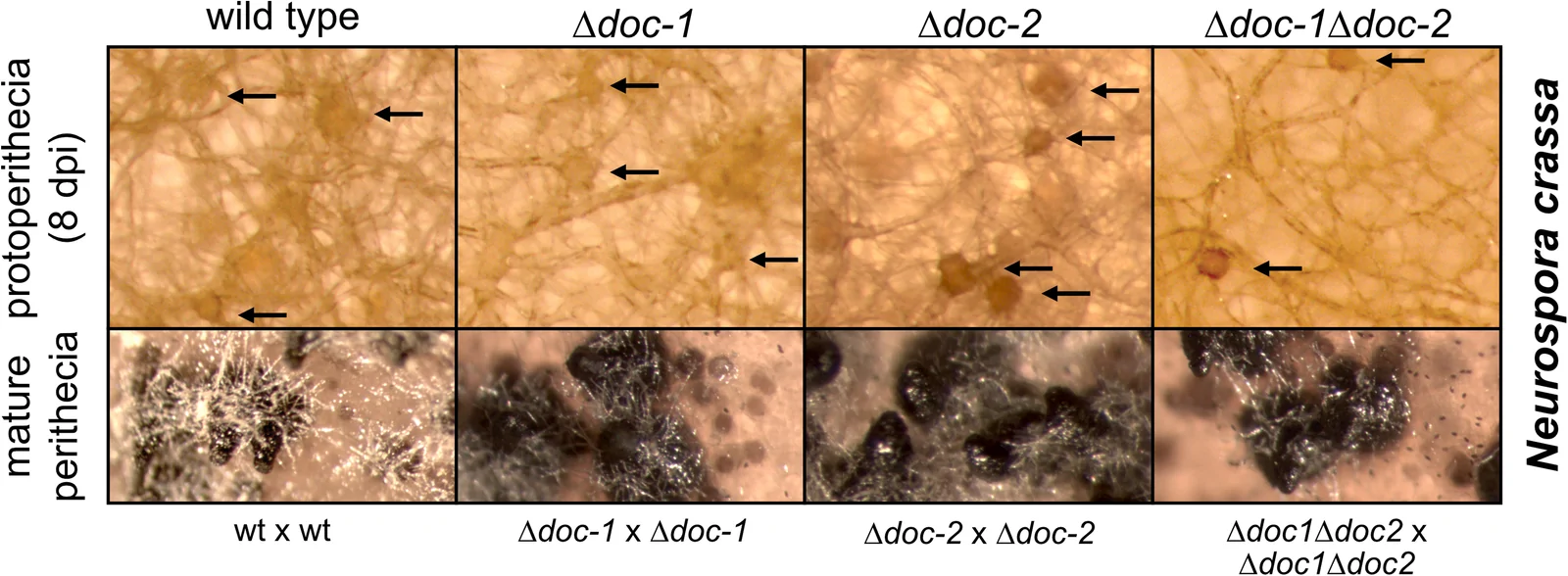
DOC-1 and DOC-2 are dispensable for sexual development in *N. crassa.* Sexual crosses of the wild type, Δ*doc-1*, Δ*doc-2* and Δ*doc-1*Δ*doc-2* of the heterothallic ascomycete *N. crassa*. Formation of the female sexual structures, the protoperithecia (indicated by black arrows), is comparable in the mutants and the wild type. Fertilization of the protoperithecia with conidia resulted in mature perithecia containing ascospores. Post-fertilization development is comparable in all strains. The images show representative pictures. dpi, days post-inoculation.

Due to the severe impairment in sexual development in the homothallic *S. macrospora*, we questioned whether sexual development of the heterothallic *N. crassa doc* knockout strains was impaired. Previous studies in *N. crassa* focused on aspects of pre-contact communication in germlings, but did not investigate the sexual development. The *N. crassa* Δ*doc-1* and Δ*doc-2* deletion strains were described to be “macroscopically indistinguishable” from the commonly used lab strain FGSC 2489 during vegetative growth [11]. To assess a potential role of DOC-1 and DOC-2 in sexual development, Δ*doc-1*, Δ*doc-2*, and Δ*doc-1*Δ*doc-2* deletion strains were phenotypically characterized under conditions promoting sexual development. All mutant strains formed female sexual structures (protoperithecia) with timing comparable to the *N. crassa* wild type (Fig 3). Following fertilization, these structures produced ascospores in a wild-type manner, indicating that DOC-1 and DOC-2 are dispensable for sexual development.

### The SmDOC1/2-proxiomes include components of the MAK2 MAP kinase cascade

**Fig 4:**
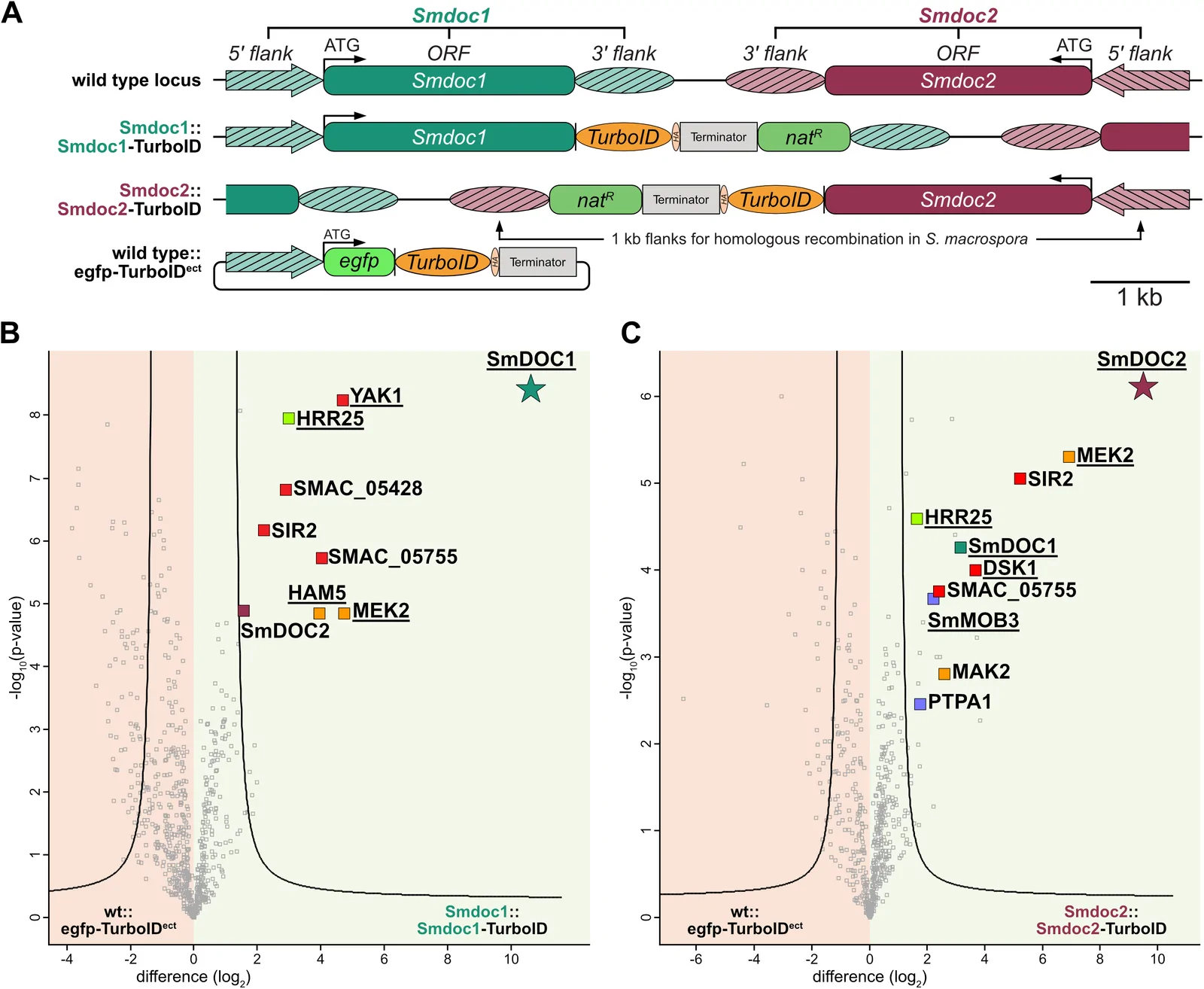
Volcano plot analysis of the SmDOC1-BioID and SmDOC2-BioID experiments. **(A)** Genomic organization of the *Smdoc* locus in *S. macrospora.* The *Smdoc1/2-TurboID* fusion constructs were integrated at their respective native locus. Each fusion gene is accompanied by a nourseothricin resistance cassette (*nat*^*R*^). Homologous recombination was performed using the 1 kb 5′ and 3′ flanking regions. For relative quantification, we ectopically integrated an *egfp-TurboID* fusion gene under the control of the 1 kb 5′ *Smdoc1* region into the *S. macrospora* wild type (wt::egfp-TurboID^ect^). Expression of the *TurboID* ligase is terminated by the terminator of the *anthranilate synthase trpC* gene of *A. nidulans*. TurboID is C-terminally tagged with a triple HA-Tag. **(B, C)** The graphs represent independent experiments, each relying on their own replicates of the wt::egfp-TurboID^ect^ control strain for relative quantification. The graphs plot the difference in Label-Free Quantification (LFQ) intensities (log_2_ transformed) of the control strain on the left and the *Smdoc-TurboID* fusion on the right. The -log_10_(p-value) is plotted on the y-axis. Significantly enriched proteins are separated from non-significant proteins by the plotted curve. Proteins that were identified with biotin site information are underlined. The bait protein is marked with a star colored in green (SmDOC1) or magenta (SmDOC2). Among the significantly enriched proteins, components of the MAK2 pathway are marked in orange, the casein kinase HRR25 is marked in light green and the SmSTRIPAK-associated proteins SmMOB3 and PTPA1 are marked in blue. The strains were grown in liquid BMM medium for three days at 27 °C under constant light. Statistical parameters of the volcano plots: FDR = 0.01; s0 = 2. **(B)** Statistical volcano plot analysis of the BioID experiment using SmDOC1-TurboID (*n* = 5) as bait. In total 1563 proteins were identified and filtering for four out of five valid values reduced the protein count to 720. The data underlying this figure can be found in S10 and S11 Data. **(C)** Statistical volcano plot analysis of the BioID experiment using SmDOC2-TurboID (*n* = 4) as bait. In total 1279 proteins were identified and filtering for four out of five valid values reduced the protein count to 573. The data underlying this figure can be found in S12 and S13 Data. *nat*^*R*^*,* nourseothricin resistance cassette expressing the *nourseothricin acetyltransferase* gene from *Streptomyces noursei* under the control of the constitutive *trpC* promoter from *A. nidulans*.

To systematically investigate the putative interaction networks of SmDOC1 and SmDOC2 in vivo, we constructed strains expressing the *Smdoc1-TurboID* or *Smdoc2-TurboID* fusions from their native loci, under the control of their native 5′ regions (Fig 4A). This approach aims to preserve endogenous regulatory networks and maintain physiological expression levels, avoiding artifacts that could arise from ectopic integration or overexpression systems.

Both C-terminally tagged *Smdoc*-fusions were integrated via homologous recombination into the Δku80 strain using 1 kb flanking regions [47]. The *Smdoc* baits were fused to *TurboID* via a GGGGSGGGS linker to allow flexibility. Transcription is terminated by the terminator of the *anthranilate synthase* gene of *A. nidulans* [48] (Fig 4A). The thereby generated Smdoc1::Smdoc1-TurboID and Smdoc2::Smdoc2-TurboID strains were analyzed by PCR and Southern hybridization experiments to verify the correct integration of the bait-TurboID fusion construct and to verify the absence of the wild-type *Smdoc1* or *Smdoc2* gene (S14 and S15 Figs). Importantly, both strains retained fertility and did not show any phenotypes of the knockout strains, demonstrating functionality of the *Smdoc-TurboID* fusions (S12 and S13 Figs). While the SmDOC1-TurboID (130 kDa) and SmDOC2-TurboID (133 kDa) fusion proteins exhibit large molecular weights, the unfused TurboID ligase in the control strain wt::free-TurboID^ect^ (39 kDa) appears small by comparison. However, large proteins are often exposed to translational challenges, including the higher risk of translational errors or co-translational misfolding due to the prolonged synthesis duration [49,50]. To balance the size discrepancy between the SmDOC-fusion proteins and the free TurboID control, we constructed an appropriate BioID control by genetically fusing *egfp* to *TurboID* via a GGGGSGGGGS linker. We chose *egfp*, because it is known to fold autonomously and has been extensively validated for its use in *S. macrospora* in complementation assays of gene knockouts, fluorescence microscopy and GFP-Trap experiments [24,51–53]. The resulting *egfp-TurboID* fusion gene encoded a protein of 66 kDa and was put under the control of the 1 kb 5′ region of *Smdoc1* rather than an overexpression or constitutive promoter to more closely match the expression and biotinylation activity of the SmDOC-TurboID fusion proteins. The *egfp-TurboID* construct was ectopically integrated into the *S. macrospora* wild type and yielded fertile single spore isolates. The biotinylation activity of the SmDOC1-TurboID, SmDOC2-TurboID and EGFP-TurboID fusion proteins was assessed in western blot experiments using a Streptavidin-HRP conjugate for signal detection (S16 and S17 Figs). Fluorescence microscopy of the wt::egfp-TurboID^ect^ strain showed uniform EGFP signal in the cytoplasm without hotspots, which could indicate protein aggregation or degradation (S18 Fig). The wt::egfp-TurboID^ect^ control strain was used for relative quantification in the independent BioID experiments using either SmDOC1 or SmDOC2 as bait. The two independent experiments showed considerable overlap among the significantly enriched proteins (Fig 4B and 4C). The bait itself was the most highly enriched protein in each experiment. The SmDOC1-BioID identified a total of 14 phosphorylated residues and 13 biotinylated sites for SmDOC1, while the SmDOC2-BioID recovered nine distinct phosphorylated residues and 12 biotinylated sites for SmDOC2. Interestingly, there was reciprocal enrichment of SmDOC2 using SmDOC1 as bait and vice versa. Both experiments enriched components of the PR pathway, namely HAM5 (SMAC_0247) in the SmDOC1-BioID, MAK2 (SMAC_03492) in the SmDOC2-BioID and MEK2 (SMAC_06526) in both experiments. Additionally, both experiments enriched the protein SMAC_05755 containing a domain of unknown function 7624, the casein kinase HRR25 (SMAC_01363) and the deacetylase SIR2 (SMAC_12019). Other significantly enriched proteins in the SmDOC1-BioID experiment included (sorted from high to low enrichment values): the protein kinase YAK1 (SMAC_02146) and the putative septal pore-associated protein SMAC_05428. Biotin site information in the SmDOC1-BioID experiment was recovered for the bait SmDOC1 itself, YAK1, MEK2, HAM5 and HRR25. Among the other significantly enriched proteins of the SmDOC2-BioID were (sorted from high to low enrichment values): the protein kinase DSK1 (SMAC_01589), the SmSTRIPAK subunit SmMOB3 (SMAC_00877) and the PP2A phosphatase activator PTPA1 (SMAC_03446). Biotin site information was recovered for peptides of SmDOC2, MEK2, SmDOC1, DSK1 and HRR25. Proteins identified in SmDOC1 and SmDOC2-BioID-experiments are listed in S10–S13 Data.

**Fig 5:**
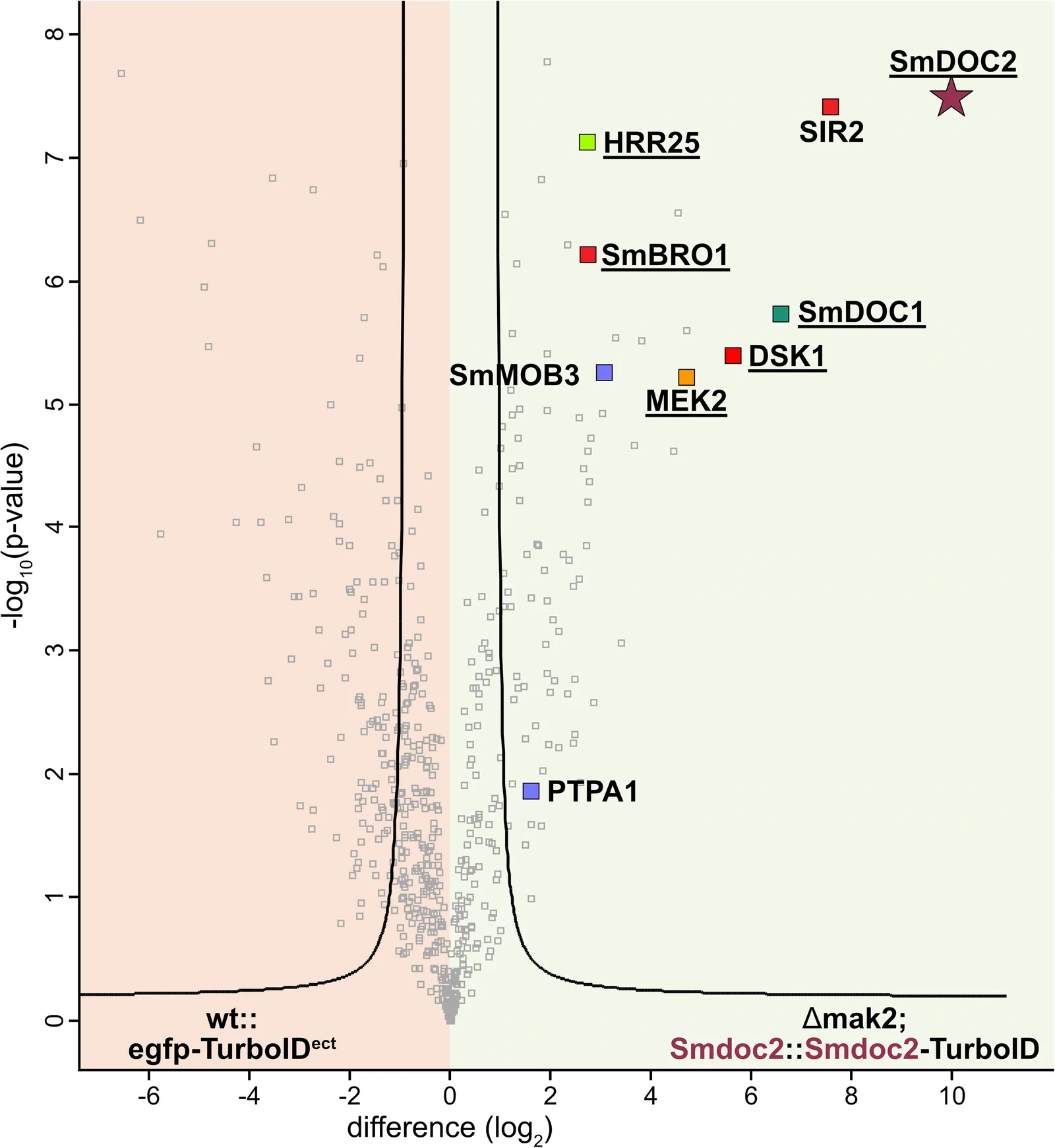
Volcano plot analysis of the MAK2-dependent environment of SmDOC2-TurboID. The Δmak2; Smdoc2::Smdoc2-TurboID strain was constructed by crossing the Smdoc2::Smdoc2-TurboID strain from the previous experiment with the Δmak2 deletion strain. The graph plots the difference in label-free quantification (LFQ) intensities (log_2_ transformed) of the control strain on the left and the SmDOC2-TurboID fusion in the Δmak2 deletion strain on the right. The −log_10_(*p*-value) is plotted on the *y* axis. Significantly enriched proteins are separated from non-significant proteins by the plotted curve. Proteins that were identified with biotin site information are underlined. Among the significantly enriched proteins, components of the MAK2 pathway are marked in orange, the casein kinase HRR25 is marked in light green and the SmSTRIPAK-associated protein SmMOB3 and PTPA1 are marked in blue. The strains were grown in liquid BMM medium for four days at 27 °C under constant light. Statistical parameters of both volcano plots: FDR = 0.01; s0 = 0.1; *n* = 4. The data underlying this figure can be found in S14 and S15 Data. *nat*^*R*^*,* nourseothricin resistance cassette expressing the *nourseothricin acetyltransferase* gene from *S. noursei* under control of the constitutive *trpC* promoter from *A. nidulans*.

Given the prominent and overlapping enrichment of components belonging to the PR signaling cascade (HAM5 and MEK2 in SmDOC1-BioID; MEK2 and MAK2 in SmDOC2-BioID), we constructed a pathway-specific BioID control strain to dissect the MAK2-dependent interactions of SmDOC2. The *S. macrospora* Δmak2 deletion is sterile, exhibits a hyphal fusion defect and is impaired in ascospore germination [8]. The new BioID strain was constructed by crossing the Δmak2 deletion strain in the spore color mutant background fus1−1 with the Smdoc2::Smdoc2-TurboID (*nat*^*R*^) strain. Spores were picked from recombinant perithecia, and sterile isolates (*nat*^*R*^) were tested for the deletion of *mak2* by PCR (S19 Fig). The resulting Δmak2; Smdoc2::Smdoc2-TurboID strain was used to explore the MAK2-dependent proxiome of SmDOC2-TurboID. The biotinylation activity of the SmDOC2-TurboID fusion protein was not affected by the introduction of the Δmak2 deletion strain background (S20 Fig). Consistent with the *mak2* deletion, MAK2 peptides were absent from the BioID eluates as well as the input controls of Δmak2, Smdoc2::Smdoc2-TurboID. The significant enrichment of the proteins SIR2, SmDOC1, DSK1, MEK2, SmMOB3, PTPA1 and HRR25 was not affected in the Δmak2 deletion background when compared to the wt::egfp-TurboID^ect^ strain (Fig 5). Biotin sites were detected for SmDOC2, MEK2, SmDOC1, DSK1 and HRR25 in the replicates of Δmak2; Smdoc2::Smdoc2-TurboID. The LFQ intensities for SIR2, SmDOC1 and DSK1 were slightly increased in the Δmak2 background when compared to the Smdoc2::Smdoc2-TurboID strain, whereas the intensity of MEK2 was reduced upon deletion of *mak2* (S14 Data). An additional candidate among the significantly enriched protein is SmBRO1 (SMAC_01835), a homolog of the *N. crassa* BRO1 (NCU08001). BRO1 was reported to be essential in *N. crassa* and investigation of *bro1* knockdowns revealed a defect in germling fusion [54]. However, it needs to be noted that the enrichment value of SmBRO1 in Δmak2; Smdoc2::Smdoc2-TurboID is rather low (log2 difference = 2.7), and the input controls show a slight upregulation of SmBRO1 in the Δmak2 background (S14 Data). Biotin sites of SmBRO1 were identified in all four biological replicates in the Δmak2 background, but not in the Smdoc2::Smdoc2-TurboID strain, the wt::egfp-TurboID^ect^ control strain or the input control samples.

### SmDOC1/2 BioID experiments enrich two MEK2 isoforms

Previous proteogenomic analysis of *S. macrospora* identified novel alternative splicing events, contributing to a refinement of the genome annotation [55]. Among them, a novel intron retention event was identified that affects the PR signaling component MEK2, resulting in an extended MEK2 isoform with an alternative and extended protein C-terminus (MEK2-t2). Proteomic analysis showed a downregulation of the MEK2-t2 isoform during sexual development starting at 3 days of incubation [55]. The SmDOC1- and SmDOC2-BioID experiments enriched unique peptides of both the shorter MEK2-t1 and the novel MEK2-t2 isoform in the BioID eluates (S16 and S17 Data). Interestingly, no MEK2-t2 peptides were identified in the BioID eluates of control samples or the input control samples.

### SmDOC1 physically interacts with components of the PR signaling pathway

**Fig 6:**
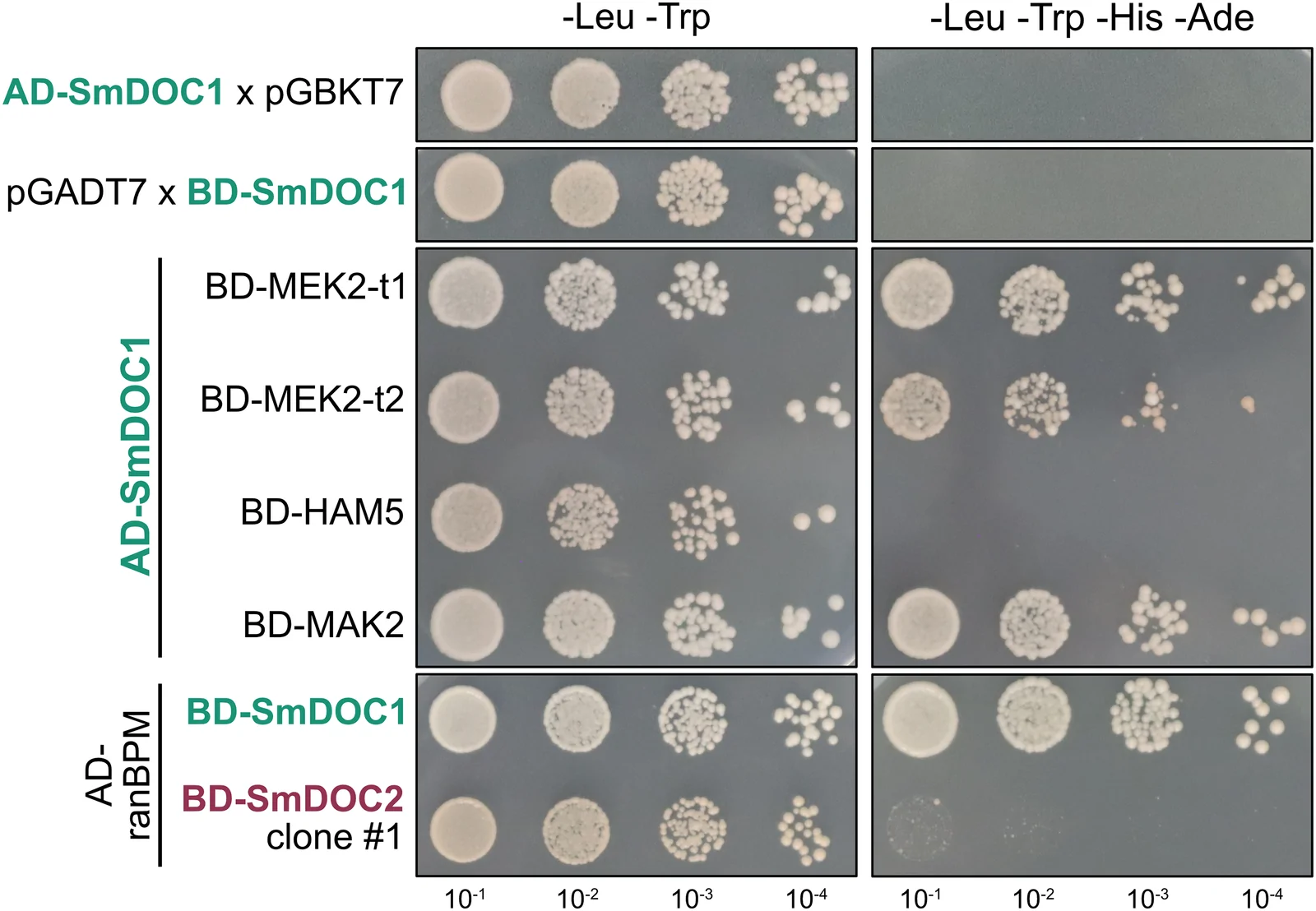
Yeast two-hybrid assay for the SmDOC1 interaction with PR components. Constructs of *Smdoc1*, *mek2-t1*, *mek2-t2*, *ham5* and *mak2* were either fused to the Gal4 activation domain (AD) or the Gal4 DNA-binding domain (BD). Serial dilutions of the yeast cells (10^−1^ = 0.5 × 10^5^ cells) were spotted onto synthetic defined (SD) medium lacking leucine and tryptophan (-Leu-Trp) or lacking leucine, tryptophan, histidine and adenine (-Leu-Trp-His-Ade). Cells harboring both plasmids (AD and BD) grow on SD-Leu-Trp. Positive interactions are indicated by hybridized yeast cell survival on selective SD-Leu-Trp-His-Ade medium. The plates were incubated at 30 °C. The full plates as well as additional clones of the AD-ranBPM × BD-SmDOC2 mating are shown in S21 Fig. AD, activation domain; BD, binding domain.

To verify the putative interactions of the SmDOC1/2 proteins with components of the PR signaling pathway proposed by the in vivo proximity labeling data, we performed Y2H analysis. For this purpose, *Smdoc1*, *Smdoc2*, *mek2-t1*, *mek2-t2*, *ham5* and *mak2* cDNAs were cloned into vectors pGBKT7 (bait) and pGADT7 (prey). These vectors harbor the Gal4 DNA-binding domain (BD) or the Gal4 activation domain (AD), respectively. Negative controls with the empty vectors pGBKT7 and pGADT7 showed that SmDOC1 does not exhibit stickiness or autoactivation activity. When SmDOC1 was fused to the AD, it was able to interact with BD-MEK2-t1, BD-MEK2-t2 and BD-MAK2 (Fig 6). An interaction of AD-SmDOC1 with BD-HAM5 was not detected. However, the BD-HAM5 fusion used here may be non-functional, since previous Y2H experiments with *S. macrospora* PR signaling pathway components were only able to demonstrate the well-characterized interaction of HAM5 with MEK2 and MAK2 when HAM5 was fused to the AD [8]. Conversely, there was no interaction when BD-SmDOC1 was mated with any of the tested AD-prey fusions, although the positive control with AD-ranBPM × BD-SmDOC1 [56] yielded positive results. Matings of the positive control AD-ranBPM with BD-SmDOC2 resulted in reduced cell viability, demonstrating an incompatibility of SmDOC2 with the Y2H assay. Therefore, protein interactions of SmDOC2 could not be tested in Y2H experiments.

### SmDOC1 appears to localize to the septal pore

**Fig 7:**
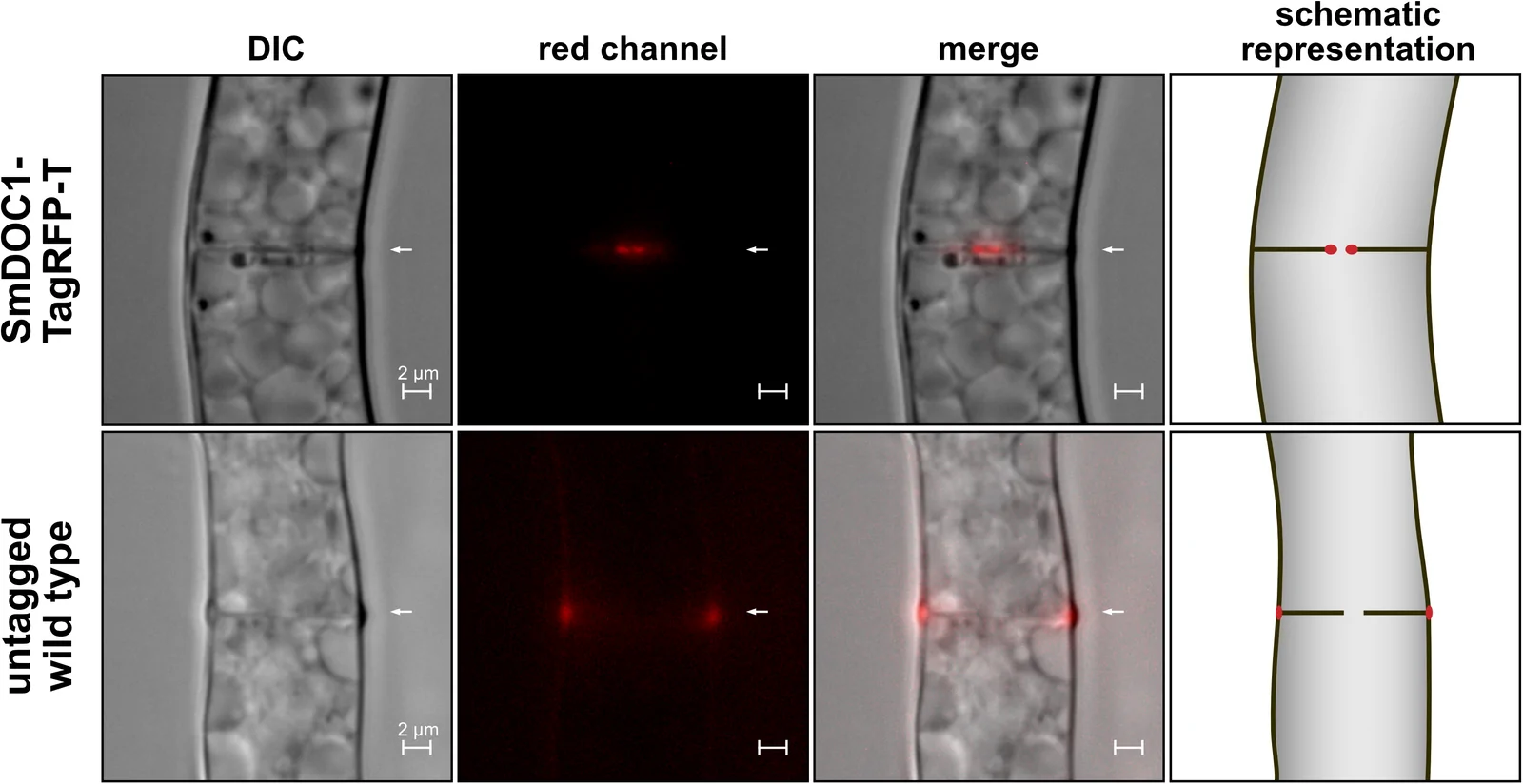
SmDOC1-TagRFP-T localizes to the septal pore. Fluorescence microscopy to determine the subcellular localization of SmDOC1. **(A)** *S. macrospora* expressing the *Smdoc1-TagRFP-T* fusion from the native *Smdoc1* locus under control of the native 5′ region. SmDOC1-TagRFP-T localizes to the center of septa in mature hyphae. The fluorescence signal is enriched at the septal pore. **(B)** Fluorescent signal of the untagged *S. macrospora* wild type, which does not express any fluorescent marker protein. The signal of the untagged wild type seems to be specific to the distal regions of the septum towards the protrusions at the cell wall. The samples were grown on microscopy slides covered in BMM. Arrows indicate septa within the focal plane. DIC, differential interference contrast; scale bars are indicated.

Fluorescence microscopy was performed to determine the subcellular localization of SmDOC1 and SmDOC2. Therefore, we performed *in locus* tagging of *Smdoc1* with the red fluorescent protein *TagRFP-T* at its C terminus [57]. The *Smdoc1-TagRFP-T* construct was integrated at the native *Smdoc1* locus using homologous recombination in the Δku80 strain based on homologous 1 kb 5′ and 3′ flanking regions. Nourseothricin-resistant single spore isolates were analyzed by PCR and Southern blot experiments to verify the absence of untagged wild-type *Smdoc1* (S14 Fig). The resulting Smdoc1::Smdoc1-TagRFP-T strain showed wild-type-like sexual development and production of fertile ascospores. Microscopic analyses of the Smdoc1-TagRFP-T strain showed weak, but distinct and consistent fluorescent signal at the center of septa in mature hyphae (Fig 7). The signal at the center of the septum is not only restricted to a single septum but is visible at all septa that are within the focal plane (S22 Fig). While the untagged *S. macrospora* wild type sometimes in a few hyphae exhibits autofluorescence in the red channel, this signal originates from the protrusions of the cell wall at the height of the septum. The untagged wild type did not exhibit any red fluorescent signal at the center of the septum. Ectopic integration of the *Smdoc1-TagRFP-T* fusion under the control of the constitutive *ccg1* promoter from *N. crassa* rather than the native *Smdoc1* 5′ region did not result in increased fluorescence signal intensity at the center of the septum (S23 Fig).

To determine the subcellular localization of SmDOC2, we generated strains expressing *egfp* and *TagRFP-T*-tagged variants of *Smdoc2* by *in locus* integration and subsequent verification by PCR and Southern blot experiments (S24 Fig). The resulting strains were fertile and did not show the ΔSmdoc2 phenotype, demonstrating the functionality of the tagged SmDOC2 fusion proteins. In both strains, only weak signals were observed in the membrane or cell wall region of the hyphae (S25 Fig). However, similar signals were detected in the untagged wild-type strain and therefore might be attributed to autofluorescence. Expression of *Smdoc2-egfp* or *Smdoc2-TagRFP-T* under the control of the constitutive *ccg1* promoter did not result in improved signal yield (S25 Fig).

## Discussion

### The proxiome of SCI1 embraces the role of the SmSTRIPAK complex as a signaling hub

This study mapped the in vivo protein neighborhood of the SCI1 subunit of the SmSTRIPAK complex in the filamentous ascomycete *S. macrospora* using proximity labeling with biotin. For this, two independent control setups were applied to assure reliable relative quantification of the mass spectrometry data and avoid false positive hits. The proxiome dataset demonstrates the capture of already known as well as previously suspected links between the SmSTRIPAK complex and other conserved fungal signaling pathways such as the SIN, MAPK signaling and ribosome biogenesis.

One example of the interaction of the SmSTRIPAK with other conserved signaling pathways is the significant enrichment of the SIN component CDC7 and the downstream protein SmBUD4. The SIN is a conserved fungal signaling pathway that coordinates cytokinesis and septum formation through a regulated kinase cascade. In filamentous fungi, SIN components contribute to proper septation, cellular compartmentalization, and developmental processes [58]. The significant enrichment of CDC7 and SmBUD4 in our BioID data coincides with the well-documented connection between the STRIPAK complex and the SIN, first established in *Schizosaccharomyces pombe*, where the STRIPAK homolog (termed SIN-inhibitory PP2A (SIP) complex) negatively regulates the SIN [41]. Additionally, our findings align with phosphoproteomic evidence from SmSTRIPAK mutant strains, which revealed SmSTRIPAK-dependent phosphorylation of the *S. macrospora* SIN components CDC7 (two sites) and SmBUD4 (five sites) [42]. More evidence for the capture of biologically relevant protein-protein interactions in the SCI1 proxiome dataset is posed by the co-enrichment of the functionally coupled pair HRR25-LTV1. This circuit is conserved from yeast to humans and functions in the biogenesis of the small 40S ribosomal subunit. The HRR25-mediated phosphorylation of the assembly factor LTV1 triggers the release of LTV1 from pre-40S ribosomal subunits and allows subunit maturation [59,60]. Deletion of LTV1 in yeast results in increased sensitivity to cold temperatures [61,62]. Notably, HRR25 was previously detected with moderate spectral counts in SCI1-eGFP pulldown experiments [45]. Phosphoproteomic analyses of the SmSTRIPAK mutant strains ΔSmpp2ac1, Δpro11, Δpro22 and the double mutant Δpro11Δpro22 reported increased phosphorylation of S134 of LTV1 [15,32]. Recent transcriptome profiling of STRIPAK mutants in the basidiomycete *Cryptococcus neoformans* suggests a connection of ribosome biogenesis with STRIPAK [63].

Furthermore, the enrichment of HAM5 in the SCI1 proxiome dataset demonstrates the link between the SmSTRIPAK complex and the PR pathway [3,8]. In *N. crassa*, HAM-5 functions as the MAP kinase scaffold protein of the PR signaling pathway, showing close associations to each of the kinases from the three-tiered cascade, demonstrated by extensive co-localization studies and positive interactions in Y2H experiments [3,4,8]. Beyond this, an interaction between the STRIPAK complex and other components of the PR signaling pathway is supported by GFP-trap experiments using the three kinases of the MAK-2 cascade in *N. crassa* as bait. This GFP-trap weakly enriched the STRIPAK subunits HAM-3 (PRO11), MOB-3 (SmMOB3), PP2A-A (SmPP2AA) and PPG-1 (SmPP2Ac1) [4]. While these data suggest a transient interaction or possibly a false positive hit, further experiments demonstrated that the STRIPAK complex and PR signaling are tightly interconnected through reciprocal regulation of phosphorylation and direct protein-protein interactions. Biochemical in vitro experiments showed phosphorylation of the STRIPAK complex subunit MOB-3 at its N-terminus by MAK-2. Additionally, an in vivo interaction of the STRIPAK with the PR signaling pathway was demonstrated by capture of MAK-2 in co-IP experiments under mild washing conditions using the PRO11 homolog HAM-3 as bait [14]. Phosphoproteomic studies in *S. macrospora* identified two phosphorylation sites of HAM5 that were differentially regulated in SmSTRIPAK deletion mutants Δpro11, Δpp2Ac1Δpro22 and Δpro11Δpro22, thus suggesting HAM5 as a substrate of the SmSTRIPAK phosphatase activity [15].

Collectively, this SCI1-proxiome dataset provides additional in vivo evidence for previously reported association of the SmSTRIPAK complex with the SIN and the PR pathway. Additionally, it proposes a role of the SmSTRIPAK complex in ribosome biogenesis by interaction with the HRR25-LTV1 circuit. These findings emphasize the STRIPAK’s role as a signaling hub in fungi and demonstrate the power of the BioID methodology to identify biologically relevant protein environments in vivo.

### SmDOC proteins regulate sexual development in *S. macrospora*

Besides its significant enrichment as the most promising candidate in the SCI1 proxiome dataset, SmDOC2 was also recovered in previous tandem affinity purification (TAP) experiments using PRO45 as bait, where it was identified with 66 spectral counts. Thereby ranking SmDOC2 the 17th out of the total 580 proteins when sorted by cumulative spectral counts of the three replicates. In this TAP-MS experiment, other SmSTRIPAK components were identified as follows: PRO11 (164 counts), SCI1 (81 counts) and SmMOB3 (22 counts) [24]. Additionally, SmDOC1 was identified in two of three SCI1-eGFP pulldown replicates [45]. The identification of SmDOC1/2 proteins as potential SmSTRIPAK-interacting proteins proposes an intriguing link between two fungal regulatory systems, and we decided to generate *Smdoc1/2* knockout strains to further investigate the interaction.

When assessing self-communication during germling fusion, Heller and colleagues reported a non-additive pattern of the *doc* knockout phenotypes in the heterothallic ascomycete *N. crassa* [11]. The single knockouts Δ*doc-1* and Δ*doc-2* showed reduced self-communication, while the double knockout Δ*doc-1*Δ*doc-2* showed wild-type levels of communication, implying that the presence of only one DOC protein disrupts balanced allorecognition. Our results extend this model and demonstrate that the DOC system is not only restricted to the heterothallic *N. crassa*, but also regulates interactions in the homothallic *S. macrospora*. Using the ΔSmdoc1, ΔSmdoc2 and ΔSmdoc1ΔSmdoc2 deletions, strains of *S. macrospora,* we showed that *Smdoc1* and *Smdoc2* play critical roles in early sexual development. While single deletions of *Smdoc1* or *Smdoc2* were severely impaired in fruiting body formation, the double deletion mutant ΔSmdoc1ΔSmdoc2 was not impaired and displayed wild-type fertility (Fig 2).

Since earlier studies of the DOC system in *N. crassa* focused on germling communication, we analyzed the *N. crassa doc* knockouts and identified no impairment in sexual reproduction. In homothallic fungi, DOC proteins may play a role that differs from that in the heterothallic fungi like *N. crassa*, potentially being more involved in the regulation of hyphal fusion associated with ascogenous hyphae formation at early sexual development stages, which are critical for subsequent perithecia formation. Despite their differences in mating systems, both *N. crassa* (heterothallic) and *S. macrospora* (homothallic), exhibit overlaps in the characteristic non-additive phenotypes of single *doc* knockout strains. This implies a conserved mechanism, requiring balanced DOC interactions for proper hyphal fusion during vegetative growth in *N. crassa* and early sexual development in *S. macrospora*.

### SmDOC1 seems to be associated with the spatial environment of the septal pore

To determine the subcellular localization, we generated *Smdoc1/2* fusions tagged with fluorescent proteins and subjected to fluorescence microscopy. However, the localization of SmDOC2 remains elusive since *egfp*- and *TagRFP-T*-tagged variants of *Smdoc2* could not be localized in fluorescence microscopy and weak signals could be attributed to wild-type autofluorescence. Additionally, the proximity labeling data does not reveal any obvious spatial environment, since DSK1, SIR2, SmMOB3, MEK2 and MAK2 localize to the nucleus, the nuclear envelope, the cytoplasm or septa in *S. macrospora* or other fungal species [8,64–67]. Previous studies localized *N. crassa* DOC-2 to the hyphal membrane and septa [11].

Fluorescence microscopy of SmDOC1-TagRFP-T under control of the native promoter suggested a localization to the center of the septum (Fig 7). These fluorescence microscopy results overlap with the results of the SmDOC1-BioID experiment, which showed significant enrichment of the proteins SMAC_05428, YAK1, MEK2 and HAM5. These proteins are associated with the septal pore in *S. macrospora* or other organisms. The *N. crassa* homolog of SMAC_05428, NCU01984 was predicted to be a septal pore-associated protein [68]. The *A. fumigatus* YakA ortholog localized to the center of septa and was shown to be involved in plugging of fungal septa upon exposure to stress. Additionally, the ΔyakA deletion strain exhibited a penetration defect into solid substrates and was unable to grow under iron-limiting conditions [69,70]. Coincident with their enrichment in the SmDOC1-BioID, the PR components MEK2 and HAM5 localize to septa and the region around the pore in *S. macrospora* [8]. Although BioID reflects proximity rather than definitive spatial localization, the overlap in enrichment of protein associated with the septal pore by the proximity labeling method in conjunction with the weak fluorescent signal of SmDOC1-TagRFP-T suggests that SmDOC1 localizes to the septal pore or the environment surrounding it. The localization of SmDOC1 to the septal pore suggests a potential role in regulating processes associated with intercompartmental communication during sexual development. In filamentous ascomycetes, septa are not complete barriers but contain central pores that permit cytoplasmic continuity and regulated movement of molecules and organelles between adjacent hyphal compartments. These pores therefore represent specialized subcellular structures that contribute to the coordination of growth, differentiation, and developmental transitions [71,72].

### SmDOC1/2 appear to be interwoven with PR signaling components

Proximity labeling experiments using SmDOC1 or SmDOC2 as bait showed consistent significant enrichment of components of the MAK2 MAPK cascade. The PR component HAM5 was captured only by SmDOC1, MAK2 only by SmDOC2, whereas MEK2 was significantly enriched using both SmDOC proteins as bait. Moreover, our proximity labeling data showed significant reciprocal enrichment of SmDOC2 when using SmDOC1 as bait, and vice versa, indicating a direct or closely associated interaction between the two SmDOC proteins. Since biotinylation is a highly specific post-translational modification, mainly restricted to histones or carboxylases [73–75], the capture of biotinylated peptides of SmDOC1, HAM5 and MEK2 poses additional evidence indicating artifact-free data and true positive hits.

The close ties of SmDOC1/2 to the PR signaling pathway are also reflected by the Y2H experiments (Fig 6). Here, SmDOC1 interacted with MEK2-t1, MEK2-t2 and MAK2, but not HAM5. However, the absence of HAM5 interaction might be caused by the incompatibility of fusion of the BD to HAM5. Y2H experiments with SmDOC2 indicated potential toxicity effects and overall incompatibility with the assay, illustrated by the drastically reduced cell viability of the positive control AD-ranBPM × BD-SmDOC2 (S21D Fig).

Beyond the direct interactions in Y2H, the SmDOC1/2 BioID experiments significantly enriched PR proteins themselves and proteins that have been reported to be associated with PR pathway components. Among them, the uncharacterized protein SMAC_05755, the *N. crassa* homolog of NCU02606, was significantly enriched in both SmDOC proxiome datasets. The protein NCU02606 was previously identified in *N. crassa* GFP-trap experiments using the PR kinases MAK-2, MEK-2 and NRC-1 as bait. However, the ΔNCU02606 deletion strain did not exhibit any impairments in germling communication assays of *N. crassa* [4]. Another example for the close association of SmDOC1/2 with the PR pathway is demonstrated by the enrichment of SIR2, which has functional ties to MEK2 in human cell systems. Downregulation of SIRT2 (homolog of *S. macrospora* SIR2) in human cells caused increased acetylation of the MAPKK MEK1 (homolog of *S. macrospora* MEK2) and resulted in hyperactivation of extracellular signal-regulated kinases (ERKs) 1/2 (homolog of *S. macrospora* MAK2). Immunoprecipitation experiments demonstrated the direct protein–protein interaction of MEK1 and ERK1/2 with SIRT2 [76,77]. Interestingly, a feedback loop was discovered in which ERK1/2 activation increased protein levels of SIRT2, its protein stability and its deacetylase activity [78]. While it is not clear whether the PR pathways-associated proteins SMAC_05755 or SIR2 are functionally connected to SmDOC1/2, their co-capture in the BioID datasets demonstrates that SmDOC1 and SmDOC2 occupy overlapping molecular neighborhoods in vivo.

### SmDOC1/2 might aid in disassembly of the PR signaling complex

**Fig 8:**
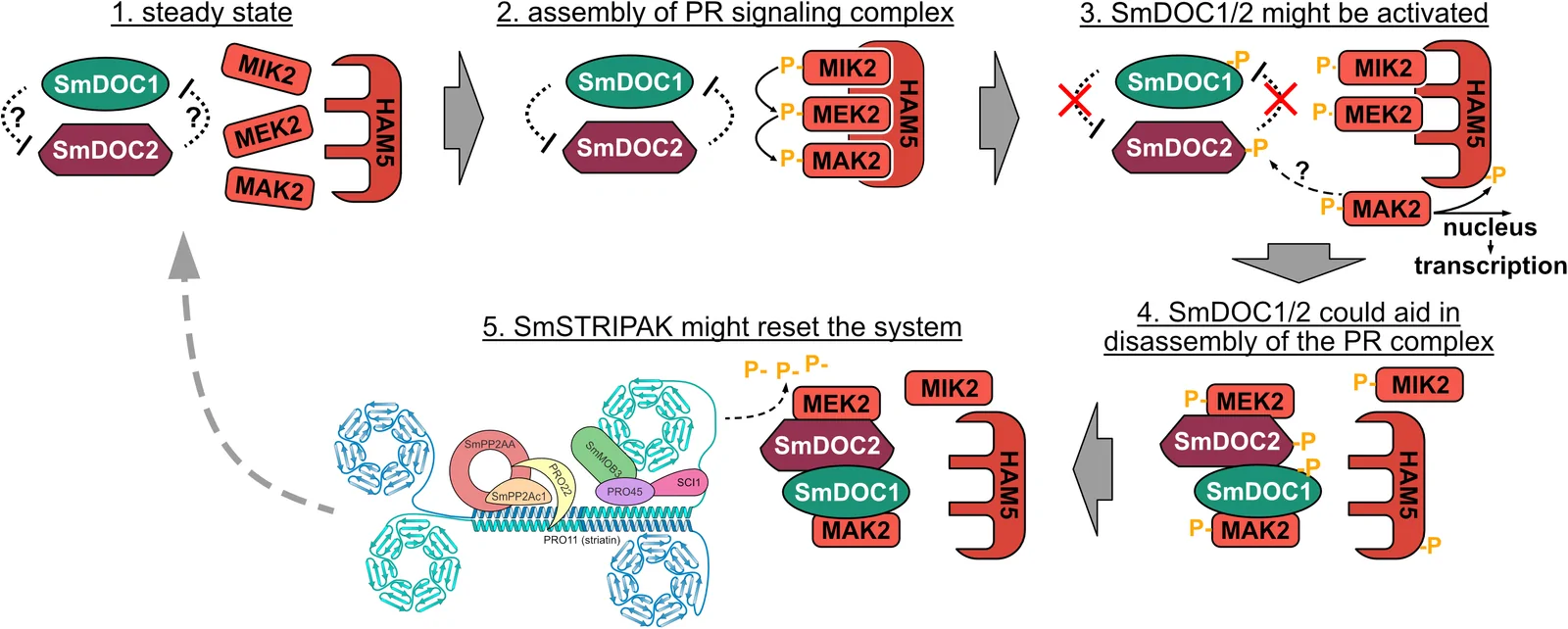
Hypothetical model of SmDOC1/2 in a homothallic interaction. This hypothetical schematic describes one possible mechanism for the function of SmDOC1/2 in *S. macrospora*. We speculate that SmDOC1/2 might regulate PR complex assembly through a cyclic mechanism. (1) In the inactive state, SmDOC1 and SmDOC2 might suppress each other’s activity by direct or indirect reciprocal inhibition. (2) Upon chemotropic signaling, the PR MAP kinase cascade (MIK2/MEK2/MAK2/HAM5) assembles, and phosphorylation is passed downstream along the cascade until the terminal MAK2 kinase is phosphorylated. (3) We speculate that at this point phosphorylated MAK2 or associated kinases might phosphorylate and activate SmDOC1/2, which could release their reciprocal repression. (4) Activated SmDOC1/2 might bind directly to the PR components, MEK2, MAK2 and HAM5, facilitating the disassembly of complex and thereby terminating PR signaling. (5) The SmSTRIPAK phosphatase might reset the system by dephosphorylating SmDOC1/2 and PR components, which would return the system to its initial state. This speculative model is based on the combined genetic, proteomic, interaction, and localization data. The dotted lines indicate unclear and hypothetical relationships. It should be noted that direct experimental validation of the underlying molecular mechanisms will require future studies.

Taking all results into consideration, we hypothesize the following mechanistic principles for the SmDOC system. (1) Due to the paradoxical and non-additive phenotypes of *doc* single deletion strains in *S. macrospora* and *N. crassa*, SmDOC1/2 likely exhibit an inhibitory effect on sexual development, rather than an activating stimulus. Hence, the double deletion strain ΔSmdoc1ΔSmdoc2, which does not encode any SmDOC protein, is not impaired in sexual development. (2) SmDOC1 and SmDOC2 are not redundant in their function. (3) A mutual antagonism mechanism might regulate the activity of SmDOC1 and SmDOC2. If only one of the components is absent, the mutual inhibitory effect on the remaining SmDOC protein (e.g., SmDOC1 in ΔSmdoc2 or SmDOC2 in ΔSmdoc1) is lifted, which in turn leads to the suppression of sexual development by the remaining SmDOC protein. Alternatively, the proteins could function in competing pathways where their balanced activity is crucial for normal development, and the complete absence of both pathways allows for the engagement of alternative developmental mechanisms. Based on the reciprocal capture of SmDOC1 in the proximity labeling data of SmDOC2-TurboID, and vice versa, we speculate that the SmDOC proteins could interact with each other, possibly by forming a heterodimer. Although due to the incompatibility of SmDOC2 in the Y2H experiments, we could not verify any physical interaction between SmDOC1 and SmDOC2. (4) The proximity enrichment of MEK2, MAK2, HAM5 and proteins associated with the PR complex, direct interaction of SmDOC1 with MEK2 and MAK2 in Y2H assays and overlapping subcellular localization in fluorescence microscopy, all converge on a model in which SmDOC1/2 might operate in close proximity or even directly interact with the PR signaling pathway. We hypothesize that SmDOC1/2 could assist in disassembly of the PR signaling complex to regulate sexual development in *S. macrospora* based on the following hypothetical model (Fig 8). The inactive system would be characterized by reciprocal inhibition of SmDOC1 and SmDOC2. Upon chemotropic interactions, the PR MAP kinase cascade of MIK2, MEK2, MAK2 and the scaffold HAM5 assembles, and phosphorylation of the kinase cascade takes place. Once the terminal kinase, MAK2 is phosphorylated, it translocates to the nucleus and regulates the expression of fusion-related genes [7,79]. During this step, active MAK2 phosphorylates HAM5, initiating a negative feedback mechanism, which leads to disassembly of the MAPK complex [3]. We speculate that at this stage SmDOC1/2 are activated, e.g., by phosphorylation through MAK2 or another kinase, which could lift the reciprocal repression cycle of the SmDOC proteins. Active SmDOC1/2 might bind to or directly interact with MEK2, MAK2 and HAM5, thereby aiding in the disassembly of the signaling complex and supporting the termination of the PR signaling response. In the last step, the SmSTRIPAK could reset the cycle by its phosphatase activity, dephosphorylating SmDOC1/2 and possibly components of the PR pathway. This would return the system to its initial state. This hypothetical mechanism could explain the observed non-additive phenotypes of the *Smdoc* knockouts in conjunction with the enrichment of PR components in BioID experiments in *S. macrospora.* This model should be viewed as a testable hypothesis and starting point for further research rather than an experimentally validated mechanism. Especially the temporal dynamics of the interaction model escape the detection of our current BioID setup and require context-specific approaches with higher resolution on the temporal scale such as split-BioID.

Since our experiments in *S. macrospora* were performed in homokaryotic cultures, lacking any allelic variety of *Smdoc1/2*, our results and therefore this model reflect a compatible allorecognition interaction. When extending our model to incompatible interactions, we speculate that SmDOC1/2 might be regulated by a two-step activation mechanism through multi-site phosphorylation. The sensing of an allelic incompatibility through a receptor could lead to additional phosphorylation and activation of SmDOC1/2, further increasing the inhibitory activity on the PR complex. The regulatory potential of SmDOC1 and SmDOC2 is demonstrated by the high number of phosphosites that were identified in the proximity labeling data (14 and 9 distinct phosphosites, respectively). Multi-phosphorylated SmDOC1/2 might bind PR components more tightly without releasing them. This would result in a situation of competitive inhibition, which prevents the assembly of the MAPK signaling cascade and thereby suppresses oscillation. However, this theory of SmDOC1/2 as phosphorylation-gated inhibitors requires extensive experimental validation of the biological relevance of the individual phosphosites.

## Methods

### Generation of *S. macrospora* strains

The plasmids used in this study are listed in S1 Table and the primers are listed in S2 Table. The plasmid pHRSmdoc1-TurboID was cloned by amplifying the *Smdoc1* 1 kb 5′ flank with primer pair LH88/LH89 from *S. macrospora* wild-type gDNA, the *Smdoc1*-ORF with primer pair LH90/91 from *S. macrospora* wild-type gDNA, the *TurboID+TtrpC* sequence with primer pair LH98/LH93 from p5′-sci1-L-TurboID, the *nat*^*R*^ with primer pair LH94/95 from pRS_nat, and the *Smdoc1* 1 kb 3′ flank with primer pair LH96/LH97 from *S. macrospora* wild-type gDNA. The fragments were integrated into *Not*I- and *EcoR*I-linearized pRS426 using the NEBuilder HiFi DNA Assembly kit (New England Biolabs GmbH, E2621S) according to the manufacturer’s instructions.

The plasmid pHRSmdoc1-TagRFP-T was cloned by amplifying the *Smdoc1* 1 kb 5′ flank with primer pair LH88/LH89 from *S. macrospora* wild-type gDNA, the *Smdoc1*-ORF with primer pair LH90/91 from *S. macrospora* wild-type gDNA, the *TagRFP-T+TtrpC* sequence with primer pair LH92/LH93 from p5′vac14-TagRFP-T, the *nat*^*R*^ with primer pair LH94/95 from pRS_nat, and the *Smdoc1* 1 kb 3′ flank with primer pair LH96/LH97 from *S. macrospora* wild-type gDNA. The fragments were integrated into *Not*I- and *EcoR*I-linearized pRS426 using the NEBuilder HiFi DNA Assembly kit (New England Biolabs GmbH, E2621S) according to the manufacturer’s instructions. The plasmid pHRccg1Smdoc1-TagRFP-T was generated to put the *Smdoc1-TagRFP-T* fusion under control of a constitutive promoter. Therefore, pHRSmdoc1-TagRFP-T was linearized by *EcoR*I and a fragment containing the constitutive *ccg1* promoter was amplified from pc-sci1-TurboID using the primer pair LH109/LH110 and inserted in between the *Smdoc1* 1 kb 5′ flank and the start codon of *Smdoc1*.

The plasmid p5′Smdoc2-L-TurboID for ectopic integration was cloned by amplifying the *Smdoc2* promoter and *Smdoc2* ORF from *S. macrospora* wild-type gDNA with primers LH05/LH09 and the sequences for TurboID with the 3× HA Tag and T*trpC* terminator were amplified from p5′-sci1-L-TurboID using primers TtrpC_pRS_r/ SmtBioID-L-f-2. Both fragments were cloned into *Xho*I-linearized pRS_nat using the NEBuilder HiFi DNA Assembly kit (New England Biolabs GmbH, E2621S) according to the manufacturer’s instructions.

For construction of the plasmid pHRSmdoc2-TurboID, the *S. macrospora Smdoc2* promoter, *Smdoc2*-ORF and TurboID with the 3x HA Tag and T*trpC* terminator were amplified from plasmid p5′Smdoc2-L-TurboID with primers LH05/LH37. The *nat*^*R*^ was amplified from plasmid pRS_nat using primers LH38/nat-1r. The 3′ flank of *Smdoc2* was amplified from *S. macrospora* wild-type gDNA with primers LH36/LH08. The resulting fragments were integrated into *Xho*I-linearized pRS426 using the NEBuilder HiFi DNA Assembly kit (New England Biolabs GmbH, E2621S) according to the manufacturer’s instructions.

The plasmid pHRSmdoc2-egfp was cloned by amplifying the *Smdoc2* 1 kb 5′ flank with primer pair LH61/62 from *S. macrospora* wild-type gDNA, the *Smdoc2*-ORF with primer pair LH63/64 from *S. macrospora* wild-type gDNA, the *egfp-TtrpC* sequence with primer pair LH65/66 from template p1783-1 and the *nat*^*R*^ fused to the Smdoc2 1 kb 3′ flank with primer pair LH67/68 from template pHRSmdoc2-TurboID. The fragments were integrated into *Not*I-linearized pRS426 using the NEBuilder HiFi DNA Assembly kit (New England Biolabs GmbH, E2621S) according to the manufacturer’s instructions. The plasmid pHRccg1Smdoc2-egfp was generated to put the *Smdoc2-egfp* fusion under control of a constitutive promoter. Therefore, pHRSmdoc2-egfp was linearized by *EcoR*I and *Bgl*II and a fragment containing the constitutive *ccg1* promoter was amplified from pc-sci1-TurboID using the primer pair LH86/LH87 and inserted in between the *Smdoc2* 1 kb 5′ flank and the start codon of *Smdoc2* using the NEBuilder HiFi DNA Assembly kit (New England Biolabs GmbH, E2621S) according to the manufacturer’s instructions.

The plasmid pHRSmdoc2-TagRFP-T was cloned by amplifying the *Smdoc2* 1 kb 5′ flank and the *Smdoc2* ORF with primer pair LH111/112 from pHRSmdoc2-TurboID, the *TagRFP-T*+*TtrpC*+*nat*^*R*^ sequence with primer pair LH113/114 from pHRSmdoc1-TagRFP-T and the *Smdoc2* 1 kb 3′ flank with primer pair LH115/116 from pHRSmdoc2-TurboID. The fragments were integrated into *Not*I-linearized pRS426 using the NEBuilder HiFi DNA Assembly kit (New England Biolabs GmbH, E2621S) according to the manufacturer’s instructions. For expression of the *Smdoc2-TagRFP-T* fusion under control of the *ccg1* promoter in pHRccg1-Smdoc2-TagRFP-T, the *Smdoc2* 1 kb 5′ flank, the *ccg1* promoter and the *Smdoc2* ORF were amplified with primer pair LH111/112 from template pHRccg1Smdoc2-egfp. The fragments were integrated into *Not*I-linearized pRS426 using the NEBuilder HiFi DNA Assembly kit (New England Biolabs GmbH, E2621S) according to the manufacturer’s instructions.

All *S. macrospora* strains used in this study are listed in S3 Table. The selection of recombinant strains was performed by supplementation of the antibiotics hygromycin B (110 U/ml) or nourseothricin (50 μg/ml). Transformation of *S. macrospora* was carried out as previously described [80]. Primary transformants were crossed to the color spore mutant fus1-1 [81] and single spores were isolated from recombinant perithecia. *S. macrospora* was grown in either liquid or solid biomalt maize medium (BMM) or *Sordaria* Westergaard’s (SWG) fructification medium at 27 °C under constant light [82,83].

The *Smdoc* knockouts were constructed by replacing the respective open reading frames (ORFs) with the hygromycin resistance cassette (*hyg*^*R*^) using homologous recombination in the Δku70 background. For construction of the knockout plasmids pdoc1-KO, pdoc2-KO and pdoc1+2-KO, the 1 kb 5′ and 3′ flanking regions were amplified from *S. macrospora* wild type (DSM997) gDNA and contain 29 bp overhangs to the pRS426 vector [84] or the hygromycin resistance cassette (*hyg*^*R*^). The 1 kb 5′ flanks were amplified using primer pairs LH01/LH02 (pdoc1-KO and pdoc1 + 2-KO) and LH05/LH06 (pdoc2-KO). The 1 kb 3′ flanks were amplified with primer pairs LH03/LH04 (pdoc1-KO), LH07/LH08 (pdoc2-KO) and LH12/LH13 (pdoc1+2-KO). The *hyg*^*R*^ cassette (1,418 bp) expresses the *hygromycin B phosphotransferase* gene (*hph*) of *Escherichia coli* under control of the constitutive *trpC* promoter from *Aspergillus nidulans* and was amplified from plasmid pSmnbr1-KO [53] with the primer pair hph-f/hph-r. The fragments were integrated into *Xho*I-linearized pRS426 by homologous recombination in *S. cerevisiae* strain PJ64-4A [85,86]. The deletion cassettes consisting of the 1 kb flanking regions and the *hyg*^*R*^ cassette were amplified from the sequenced plasmids using the primers LH01/LH04 (ΔSmdoc1), LH05/LH08 (ΔSmdoc2); LH01/LH13 (ΔSmdoc1ΔSmdoc2). The PCR amplicons were desalted and transformed into the nourseothricin-resistant *S. macrospora* Δku70 deletion strain for homologous recombination [87]. In the Δku70 deletion strain, the *ku70* gene was replaced with the nourseothricin resistance cassette (*nat*^*R*^) expressing the *nourseothricin acetyltransferase* gene of *Streptomyces noursei* under control of the constitutive *trpC* promoter from *A. nidulans*. The wild-type *ku70* gene was restored by crossing the primary transformants of the *Smdoc* knockouts with spore color mutant fus1−1 and isolating hygromycin-resistant, but nourseothricin-sensitive spores. These single spore isolates were genotyped using PCR with one of the primers binding outside of the 1 kb flanks within the genomic region to verify the 5′ and 3′ junctions of the *hyg*^*R*^ integration at the respective *Smdoc* locus. Additionally, the absence of the respective wild-type *Smdoc* ORF was verified by PCR. Southern hybridization was performed to rule out off-target integrations of the *hyg*^*R*^ cassette.

### Generation of *N. crassa* strains

The *N. crassa* strains used in this study are listed in S3 Table. To generate Δ*doc-2* strains of *Neurospora crassa*, the parental strain GN10−04 (Δ*doc-2*, *matA*, *his-3*^*−*^), which was obtained from Louise Glass in Berkeley, was crossed with the wild-type strain (*mata*). Following ascospore germination, progeny were selected on a Vogel’s minimal medium lacking histidine and supplemented with hygromycin (200 µg/ml) to isolate successfully transformed and viable recombinants. The integration of the hygromycin resistance cassette at the *doc-2* locus was verified via PCR. Primer pairs were designed to span the homologous integration site, binding upstream of the cassette and within the hygromycin sequence, thereby confirming the intended locus-specific disruption and the desired genotype.

Progeny exhibiting the correct molecular genotype were subsequently subjected to mating type testing to identify *matA* and *mata* individuals. Validated Δ*doc-2* offspring of opposite mating types were then utilized for homozygous Δ*doc-2* × Δ*doc-2* crosses. Cultivation and crossing were performed on Westergaard’s synthetic cross medium. The initial development of protoperithecia was monitored closely. After 7 days of incubation, fertilization was carried out by distributing 1 mL of an aqueous spore suspension containing the complementary Δ*doc-2* mating type across the surface of the agar plate containing the mature protoperithecia.

### Yeast two-hybrid

Yeast Two-Hybrid (Y2H) experiments were performed based on the Matchmaker Gold Yeast Two-Hybrid system (Takara Bio USA). The plasmids, primers and strains used for Y2H experiments are listed in S1–S3 Tables. The plasmids were constructed using the supplied vectors pGADT7 containing the Gal4 activation domain (AD), and pGBKT7 containing the Gal4 DNA-binding domain (BD). The coding sequences of *Smdoc1* (2,523 bp, AD LH125/126 or BD LH127/LH128), *Smdoc2* (2,646 bp, AD LH129/LH130 or BD LH131/LH132), *mek2-t1* (1,160 and 123 bp fragments, AD LH119/LH120 and LH121/LH122, BD LH123/LH120 and LH121/LH124), *mek2-t2* (1,160 and 438 bp fragments, AD LH119/LH137 and LH138/LH139, BD LH123/LH137 and LH138/LH140) and *ham5* (5,139 bp, AD LH133/LH134, BD LH135/LH136) were amplified from *S. macrospora* wild-type cDNA and integrated into *EcoR*I linearized pGADT7 or pGBKT7 via the NEBuilder HiFi DNA Assembly kit (New England Biolabs GmbH, E2621S). The plasmids pAD-mak2 and pBD-mak2 were kindly donated by [8]. The bait plasmids pGBKT7, pBD-Smdoc1, pBD-Smdoc2, pBD-mek2-t1, pBD-mek2-t2, pBD-ham5 and pBD-mak2 were transformed into *S. cerevisiae* strain Y2HGold (MATa, Takara Bio) and transformants were selected on media lacking tryptophan. The prey plasmids pGADT7, pAD-Smdoc1, pAD-Smdoc2, pAD-mek2-t1, pAD-mek2-t2, pAD-ham5, pAD-mak2 and pAD-ranBPM were transformed into strain Y187 (MATα,) and selected for leucine prototrophy. Recombinant bait and prey strains were mated and plated on SC medium lacking tryptophan and leucine to screen for the cells containing both pAD and pBD plasmids. Interactions of bait and prey fusion proteins were analyzed by drop dilution assays. Therefore, the cells were grown in selective SD medium lacking tryptophan and leucine. The cells were diluted to 0.1 optical density (OD) and 25 µL were spotted in 1:10 serial dilutions onto selective SD plates. The plates were incubated at 30 °C for 5 days.

### Light and fluorescence microscopy

For microscopic analyses, the *S. macrospora* strains were grown on sterile microscopy slides that were covered in BMM medium. The slides were inoculated with agar plugs containing mycelium. The slides were grown for 1–2 days at 27 °C under constant light conditions. The slides were analyzed using an Axio Imager M1 microscope (Zeiss, Germany). For detection of EGFP signal (termed green channel), the Chroma filter set 49002 was used and TagRFP-T signals (referred to as red channel) were acquired using the Chroma filter set 49005. Pictures were captured with the Axiocam 503 camera (Zeiss, Germany). Magnifications of fungal colonies for phenotypic analyses were taken with a VHX-500F Digital Microscope (Keyence, Japan).

### Protein extraction and western blot hybridization

In brief, the *S. macrospora* strains were grown in large Petri dishes (⌀ = 150 mm) filled with 50 ml of liquid medium. The medium was inoculated with 5–7 solid agar plugs from precultures. The strains were grown for 3–4 days at 27 °C under constant light conditions. Afterwards, the agar plugs were removed, the mycelium was pressed dry and ground in liquid nitrogen. Proteins were extracted from the mycelium powder using the lysis buffer 10 mM Tris pH 7.5, 150 mM NaCl, 0.5 mM EDTA pH 8, 1 mM PMSF, 2 mM DTT, 0.5% Nonidet-P40, 2× cOmplete EDTA-free Proteinase Inhibitor Cocktail (Roche, Switzerland), 1× PhosSTOP (Roche, Switzerland) followed by centrifugation for 20 min at 10,000*g*. Roughly 50 µg of protein was loaded onto a SDS polyacrylamide gel electrophoresis (PAGE). Blotting was performed with the Trans-Blot Turbo Transfer System (BioRad) using the Trans-Blot Turbo RTA Mini 0.2 µm Nitrocellulose Transfer Kit according to the manufacturer’s instructions. After blotting, reversible Ponceau S staining (0.1% Ponceau S in 5% glacial acetic acid) was used to stain total protein on the membrane to assess the loading. Biotinylated proteins were detected with a Streptavidin-HRP conjugate (Thermo Scientific, 21130, 1:30,000). Membranes were incubated with 500 µl WesternBright ECL HRP (Advansta Corporation, USA) substrate and documented with the digital Lourmat FUSION SL (Vilber, France) imaging system.

### BioID sample preparation

BioID experiments were performed as described in Hollstein and colleagues, 2025 [40]. For BioID experiments, the proteins were extracted from cell lysates under denaturing conditions (4% SDS) including an incubation for 5 min at 65 °C. Biotinylated proteins were enriched using 100 µl slurry of Strep-Tactin Sepharose (IBA Lifesciences GmbH, 2-1201-002) per 1 ml of crude protein extract according to the manufacturer’s instructions for batch purification. Biotinylated proteins were released from the Strep-Tactin Sepharose resin by competitive elution using the biotin-containing buffer BXT (0.1 M Tris-Cl, 0.15 M NaCl, 1 mM EDTA, 50 mM biotin, pH 8). The BXT-eluate was purified by chloroform methanol precipitation [88]. The protein pellet was resuspended in 1× SDS loading dye and separated on a polyacrylamide gel. Proteins were digested in-gel by trypsin and the eluted peptides were desalted with C18 StageTips prior to LC–MS analysis [89,90].

### LC–MS analysis

Although the overall analytical workflow is similar to that used previously study [37], the data shown here derive from independent experiments and were processed using the statistical criteria described here. Processed BioID samples were analyzed with LC–MS by the Service Unit LC–MS Protein Analytics of the Göttingen Center for Molecular Biosciences (GZMB) of the University of Göttingen. Dried peptide samples were reconstituted in 20 µl LC–MS sample buffer (2% acetonitrile, 0.1% formic acid). Two to eight µl of each sample were subjected to reverse phase liquid chromatography for peptide separation using an RSLCnano Ultimate 3000 system (Thermo Fisher Scientific): Peptides were loaded on an Acclaim PepMap 100 pre-column (100 μm × 2 cm, C18, 5 μm, 100 Å; Thermo Fisher Scientific) with 0.07% trifluoroacetic acid at a flow rate of 20 µl/min for 3 min. Analytical separation of peptides was done on an Acclaim PepMap RSLC column (75 μm × 50 cm, C18, 2 μm, 100 Å; Thermo Fisher Scientific) at a flow rate of 300 nL/min. The solvent composition was gradually changed within 94 min from 96% solvent A (0.1% formic acid) and 4% solvent B (80% acetonitrile, 0.1% formic acid) to 10% solvent B within 2 min, to 30% solvent B within the next 58 min, to 45% solvent B within the following 22 min, and to 90% solvent B within the last 12 min of the gradient. All solvents and acids had Optima grade for LC–MS (Fisher Chemical). Eluting peptides were on-line ionized by nano-electrospray (nESI) using the Nanospray Flex Ion Source (Thermo Fisher Scientific) at 1.5 kV (liquid junction) and transferred into a Q Exactive HF mass spectrometer (Thermo Fisher Scientific). Full scans in a mass range of 300–1,650 *m*/*z* were recorded at a resolution of 30,000 followed by data-dependent top 10 HCD fragmentation at a resolution of 15,000 (dynamic exclusion enabled). LC–MS method programming and data acquisition was performed with the XCalibur 4.0 software (Thermo Fisher Scientific). The LC–MS raw files were analyzed using MaxQuant version 1.6.10.43 and the detailed configuration is documented in the “mqpar.xml” file provided in the uploaded MaxQuant search results folder. Due to their vastly different nature in protein content and distribution, proteome controls were configured in a different parameter group than BioID eluate samples and the option “Separate LFQ in parameter groups” was activated in MaxQuant. This avoids the cross-quantification of BioID eluate samples with their non-enriched input controls, which reflect the whole proteome. Therefore, LFQ intensities for BioID eluates are exclusively quantified with other BioID eluate samples. The *S. macrospora* protein sequences from version 4 were used as sequence input into MaxQuant [91]. The data processing steps of LC–MS raw data with MaxQuant and the statistical evaluation with Perseus version 1.6.15.0 are listed in Table 1. The naming scheme of the LC–MS raw files is listed S18 Data.

**Table 1:** Data processing steps of LC–MS raw files.

Setting	Configuration
MaxQuant	
Variable modifications	Add “Biotinylation of Lysine” (Unimod accession #3) and Phospho (STY) as variable modification
Digestion	3 missed cleavage sites (Trypsin/P)
Label-free quantification	Activated with standard settings
Fasta files	2023-04-03-Sm_v04_wtN_proteins-Annotated.fasta
Perseus	
Generic matrix upload	proteinGroups.txt Main: LFQ intensities Numerical: MS/MS counts Multi-numerical: Biotin site positions, Phospho(STY) site positions
Filter rows based on categorical column	Remove rows containing “+” for columns “only identified by site”, “reverse” and “potential contaminant”
Transform	LFQ intensities: log2(*x*)
Categorical annotation	Combine all replicates of a strain into one group. Create groups according to the statistical comparisons that will be drawn (control vs. bait-ligase fusion).
Analysis	Multi-scatter plot to check reproducibility of the replicates Histogram to check LFQ intensity distribution Venn diagram to check protein counts
Filter rows based on valid values	Filter for valid values depending on the experimental setup and data quality, e.g., 5 out of 5 valid values in at least one group
Replace missing values from normal distribution	Mode: Separately for each column
Volcano plot	Perform volcano plot analysis to identify significantly enriched proteins using the grouping defined earlier. Choose statistical stringency based on data quality: , FDR = 0.01, s0 = 2 Select significantly enriched proteins and export selection (reduce matrix)
Imputation	Replace imputed values by NaN

LC–MS raw files were processed with MaxQuant version 1.6.10.43 and statistical evaluation was performed with Perseus version 1.6.15.0.

## Supporting information

S1 Raw Images Raw images used for figure preparation. Original, unprocessed images corresponding to all images presented in the main and supplementary figures. (PDF)

S1 Table Plasmids used in this study. (DOCX)

S2 Table Primers used in this study. (DOCX)

S3 Table Strains used in this study. (DOCX)

S1 Fig Workflow of the BioID method to capture in vivo co-localizing proteins. Overview of the BioID method. The promiscuous biotin ligase (here TurboID) is genetically fused to the bait protein. Proteins in the proximal environment are covalently labeled with biotin, whereas distal proteins are not labeled. Proteins are extracted under denaturing conditions and biotinylated proteins are enriched using the streptavidin mutant Strep-Tactin. Non-biotinylated proteins are removed during washing, and only biotinylated proteins are eluted from the affinity purification resin. The proteins are then digested with trypsin and the resulting peptides are analyzed with LC–MS. Significantly enriched proteins are determined by relative quantification in comparison to the control (e.g., expressing an unfused biotin ligase). Input (total proteome) controls were taken prior to biotin affinity capture and analyzed by LC–MS to account for protein abundances within the different strains.
(TIFF)

S2 Fig Sequence alignments of *S. macrospora* SmDOC1/2 with *N. crassa* DOC-1/2. **(A, B)** Pairwise amino acid sequence alignments of **(A)** SmDOC1 (SMAC_06903) with *N. crassa* DOC-1 (NCU07191) and **(B)** SmDOC2 (SMAC_06902) with *N. crassa* DOC-2 (NCU07192). Amino acids are colored according to their conservation: regions with low conservation are shaded in white, whereas a high degree of conservation is highlighted in black. Amino acid positions are given on the left and right side of the alignment. The DOC proteins of *S. macrospora* share high sequence identities to *N. crassa*: SmDOC1 = 91.8%; SmDOC2 = 86.3%. This alignment was created with Jalview [92]. **(C)** Genomic map showing the highly syntenic loci encoding the *doc* genes on chromosome two of *S. macrospora* and *N. crassa* strain FGSC 2,489, a member of communication group haplotype 1 (CGH1). Genes that are filled in any other color than white are homologs. The region spanning from *NCU07191* up to *NCU07187* is inverted in *S. macrospora*. The *N. crassa doc* locus of CGH1 is based on [11].
(TIFF)

S3 Fig Verification of the BioID control strain Δpro11Δsci1::sci1-TurboID^ect^. Screening of the progeny from the cross Δpro11 × Δsci1::sci1-TurboID^ect^ for the construction of a BioID control strain. Single spore isolates (1–4) of the putative Δpro11Δsci1::sci1-TurboID^ect^ strain were analyzed by PCR and Southern hybridization experiments to verify **(A)** the Δsci1 background, **(B)** Δpro11 background and **(C)** the ectopic integration of the *sci1-TurboID* fusion gene under control of the native *sci1* promoter (*sci1 5′*). Single spore isolate 4 (printed in bold) was used for further experiments.
(TIFF)

S4 Fig Phenotypic characterization of the BioID control Δpro11Δsci1::sci1-TurboID^ect^. To analyze the SmSTRIPAK-dependent protein environment of SCI1-TurboID, we crossed the Δsci1::sci1-TurboID^ect^ with the Δpro11 deletion strain. The sterile Δpro11 mutant does not produce any fruiting bodies in homokaryotic cultures. The Δpro11 knockout carries the spore color mutant background fus1-1, which results in brown ascospores in crosses. Spores were selected from recombinant perithecia (with brown and black spores) and were verified for the double deletion of *sci1* and *pro11* (S3 Fig). For the growth test, the single spore isolates were grown at 27 °C on solid *Sordaria* Westergaard’s (SWG) fructification medium. Pictures of the Petri dishes, the close-ups and the lids were taken after 14 days of incubation. Once the black ascospores are fully matured inside the fruiting bodies, they are forcefully ejected towards the light source and will stick to the lids of the Petri dishes, thereby staining them black and opaque. The BioID control strain Δpro11Δsci1::sci1-TurboID^ect^ is sterile and does not produce any fruiting bodies.
(TIFF)

S5 Fig Scatterplot analysis of the SCI1-BioID input samples. Mass spectrometry analysis of the BioID input samples before biotin affinity purification of the Δsci1::sci1-TurboID strain (fertile) and the Δpro11Δsci1::sci1-TurboID control strain (sterile). A total of 4268 proteins were identified, with 2,856 of them being quantified in both strains. The Pearson correlation of this scatterplot is 0.966. This scatterplot plots the LFQ intensity of proteins which were quantified in both strains against each other. The line shows the best fit (1.006079*x). SmDOC2 and SmDOC1 are marked with red rectangles. The protein abundances of SmDOC1 and SmDOC2 do not appear to be regulated in the proteome of the given strains. The data underlying this figure can be found in S5 and S6 Data. LFQ, label-free quantification; ect, ectopic integration.
(TIFF)

S6 Fig Generation and verification of the ΔSmdoc1 deletion strain. **(A)** Genomic situation of the *Smdoc1* locus (*SMAC_06903)* in the wild type (wt) and the ΔSmdoc1 deletion strain. The amplicons of ΔSmdoc1 PCR verification reactions are indicated by black lines. The absence of the *Smoc1* open reading frame (ORF) was verified with primer pair LH27/LH28 (2,069 bp). In single spore isolates 10.5, 10.6, 10.8 and 10.9. The integration of the *hyg*^*R*^ cassette at the desired genomic locus was verified using primer pairs LH15/tC1 (1,183 bp, *hyg*^*R*^ 5′ junction) and LH16/h3 (1,238 bp, *hyg*^*R*^ 3′ junction). **(B)** Southern hybridization verification of ΔSmdoc1. The probes are indicated by red lines and the restriction enzyme sites are marked by arrows. *hyg*^*R*^, hygromycin resistance cassette expressing the *hygromycin B phosphotransferase* gene from *E. coli* under control of the constitutive *trpC* promoter from *A. nidulans*.
(TIFF)

S7 Fig Generation and verification of the ΔSmdoc2 deletion strain. **(A)** Genomic situation of the *Smdoc2* locus (*SMAC_06902*) in the wild type (wt) and the ΔSmdoc2 deletion strain. The amplicons of ΔSmdoc2 PCR verification reactions are indicated by black lines. The integration of the *hyg*^*R*^ cassette at the desired genomic locus of single spore isolates (10.3, 13.2, 13.4, 13.5, 14.1, 14.3, 14.4 and 14.5) was verified using primer pairs LH19/tC1 (1,343 bp *hyg*^*R*^ 5′ junction) and LH20/h3 (1,202 bp, *hyg*^*R*^ 3′ junction). The absence of the *Smdoc2* open reading frame (ORF) was verified with primer pair LH30/LH33 (1,675 bp). **(B)** Southern hybridization verification of ΔSmdoc2. The probe is indicated by a red line and the *Sca*I restriction enzyme sites are marked by arrows. *hyg*^*R*^, hygromycin resistance cassette expressing the *hygromycin B phosphotransferase* gene from *E. coli* under control of the constitutive *trpC* promoter from *A. nidulans*.
(TIFF)

S8 Fig Generation and verification of the ΔSmdoc1ΔSmdoc2 double knockout. Genomic situation of the *Smdoc1* (*SMAC_06903*) and *Smdoc2* (*SMAC_06902*) locus in the wild type (wt) and the ΔSmdoc1ΔSmdoc2 double deletion strain. The amplicons of the PCR verification reactions are indicated by black lines. The integration of the *hyg*^*R*^ cassette at the desired genomic locus of the single spore isolates (2.2, 2.4, 3.4, 7.2, 8.3, 8.4, 10.1 and 11.2) was verified using primer pairs LH15/tC1 (1,182 bp, *hyg*^*R*^ 5′ junction) and LH19/h3 (1,334 bp, *hyg*^*R*^ 3′ junction). The absence of the *Smdoc1* and *Smdoc2* open reading frames (ORFs) was verified with primer pair LH18/LH21 (2,820 bp). **(B)** Southern hybridization verification of ΔSmdoc1ΔSmdoc2. The probe is indicated by a red line and the *Sca*I restriction enzyme sites are marked by arrows. The single spore isolate 11.2 appears to be a heterokaryon which carries both, the deletion of *Smdoc1* and *Smdoc2* and the wild-type locus. *hyg*^*R*^, hygromycin resistance cassette expressing the *hygromycin B phosphotransferase* gene from *E. coli* under control of the constitutive *trpC* promoter from *A. nidulans*.
(TIFF)

S9 Fig Hyphal fusion events in the *Smdoc* single knockout strains. Microscopy pictures of hyphal fusion event in the vegetative mycelium of ΔSmdoc1 and ΔSmdoc2. The black stars (*) indicate a hyphal fusion with a continuous flow of cytoplasm. Pictures were taken after one day of inoculation on solid BMM plates. A scale bar (10 µm) is indicated.
(TIFF)

S10 Fig Vegetative growth rates of *Smdoc* knockouts. The vegetative growth rate was determined in race tubes experiments using the *Sordaria* Westergaard’s (SWG) fructification medium over a time frame of 10 days. **(A)** Total mycelium length three days after incubation of the race tubes. Colony establishment of ΔSmdoc1 and ΔSmdoc2, but not the double knockout, ΔSmdoc1ΔSmdoc2, is impaired. The data underlying this figure can be found in S7 Data. *n* = 4 **(B)** Average growth rate per day calculated based on growth from day 7 to 10 after inoculation. The error bars show the standard deviation from four replicates. Asterisks indicate significant differences to the wild type according to Student *t* test (*p* < 0.005, two-tailed). *n* = 4 The data underlying this figure can be found in S8 Data.
(TIFF)

S11 Fig Phenotypic characterization of *Smdoc1/2* complementation strains. The single spore isolates were grown at 27 °C on solid *Sordaria* Westergaard’s (SWG) fructification medium. While the single knockout strains ΔSmdoc1 and ΔSmdoc2 show severe impairment in sexual development, the transformation with the complementation constructs restored fruiting body formation. The complementation constructs were ectopically integrated into the knockout strains and express the open reading frame of *Smdoc1* or *Smdoc2* flanked by 1 kb upstream and downstream regions. The ΔSmdoc1::Smdoc1^ect^ complementation shows increased fruiting body formation when compared to the wild type. The Smdoc2::Smdoc2-L-TurboID strain expresses the *Smdoc2-L-TurboID* fusion from the native *Smdoc2* locus using the native promoter. Pictures of the Petri dishes, the close-ups and the lids were taken after 14 days of incubation. Once the black ascospores are fully matured within the fruiting bodies, they are forcefully ejected towards the light source and will stick to the lids of the Petri dishes. The images of the wild type, ΔSmdoc1 and ΔSmdoc2 are the same images used in Fig 2.
(TIFF)

S12 Fig Ascus rosettes of *S. macrospora Smdoc* knockouts and complementation strains. The single spore isolates were grown at 27 °C on solid *Sordaria* Westergaard’s (SWG) fructification medium. The complementation constructs were ectopically integrated into the knockout strains and express the open reading frame of *Smdoc1* or *Smdoc2* flanked by 1 kb upstream and downstream regions. The TurboID-strains express the respective *Smdoc-TurboID* fusion from the native *Smdoc* locus using the native promoter. A scale bar (100 µm) is indicated. ect, ectopic integration.
(TIF)

S13 Fig Quantification of ejected ascospores. The single spore isolates were grown at 27 °C on solid *Sordaria* Westergaard’s (SWG) fructification medium. Ejected ascospores were washed off the lids of the petri dish after 14 days of incubation and pelleted. The volume of the pellet was used to approximate the spore count using a standard which was established by wild-type spores counted in a Neubauer counting chamber. The error bars show the standard deviation from three replicates. The complementation constructs were ectopically integrated into the knockout strains and express the open reading frame of *Smdoc1* or *Smdoc2* flanked by 1 kb upstream and downstream regions. The TurboID/egfp/TagRFP-T fusion expresses the respective *Smdoc* fusion from the native *Smdoc* locus using the native promoter. The data underlying this figure can be found in S9 Data. *n* = 3. ect, ectopic integration.
(TIF)

S14 Fig *In locus* tagging of *Smdoc1* with *TurboID* and *TagRFP-T.* **(A)** Genomic situation of the *Smdoc1* (*SMAC_06903*) locus in the wild type and after the integration of *Smdoc1-TurboID* or *Smdoc1-TagRFP-T* fusions. Expression is regulated by the native *Smdoc1* 5′ region and the *TtrpC* terminator of the *anthranilate synthase* gene of *A. nidulans* was used for transcription termination. TurboID and TagRFP-T are C-terminally fused to SmDOC1 via a GGGGSGGGGS linker. TurboID is C-terminally tagged with a triple HA-tag. The amplicons of the PCR verification reaction are indicated by black lines. The integration of the fusion at the *Smdoc1* locus was verified using the PCR primer pair LH16/LH18 (*Smdoc1-TurboID* = 4,041 bp, *Smdoc1-TagRFP-T* = 3,704 bp). **(B)** Southern hybridization verification of Smdoc1::Smdoc1-TurboID and Smdoc1::Smdoc1-TagRFP-T. The probe is indicated by a red line, and the *Xho*I restriction enzyme sites are marked by arrows. *nat*^*R*^, nourseothricin resistance cassette expressing the *nourseothricin acetyltransferase* gene from *S. noursei* under control of the constitutive *trpC* promoter from *A. nidulans*.
(TIFF)

S15 Fig *In locus* tagging of *Smdoc2* with *TurboID.* **(A)** Genomic situation of the *Smdoc2* (*SMAC_06902*) locus in the wild type and after the integration of *Smdoc2-TurboID*. Expression is regulated by the native *Smdoc2* 5′ region and the *TtrpC* terminator of the *anthranilate synthase* gene of *A. nidulans* was used for transcription termination. *TurboID* is fused to the C-terminus of *SmDOC2* via a GGGSGGGS linker. Moreover, *TurboID* is C-terminally tagged with a triple HA-tag. The amplicons of the PCR verification reaction are indicated by black lines. The integration of the fusion at the *Smdoc2* locus was verified using the PCR primer pairs LH20/LH21 (4,108 bp) and LH19/Seq_tBioID_rev (4,079 bp). **(B)** Southern hybridization verification of Smdoc2::Smdoc2-TurboID. The probe is indicated by a red line and the *EcoR*I restriction enzyme sites are marked by arrows. The primary transformant (PT) 1 is a heterokaryon and exhibits signals for the wild-type locus and the transformed locus. *nat*^*R*^, nourseothricin resistance cassette expressing the *nourseothricin acetyltransferase* gene from *S. noursei* under control of the constitutive *trpC* promoter from *A. nidulans*. PT, primary transformant.
(TIFF)

S16 Fig Biotinylation activity of the EGFP-TurboID control and the SmDOC1-TurboID fusion protein. As control in SmDOC BioID experiments, the *egfp-TurboID* fusion gene under control of the *Smdoc1* 1 kb 5′ region was ectopically integrated into the *S. macrospora* wild type (wt), resulting in strain wt::egfp-TurboID^ect^. For SmDOC1 BioID experiments, the TurboID ligase was fused to the C-terminus of the SmDOC1 protein via a GGGGSGGGGS linker. The *Smdoc1-TurboID* fusion construct was integrated at the native *Smdoc1* locus (Smdoc1::Smdoc1-TurboID, ssi 9.2). To show the catalytic activity of the ligase, the strains were supplemented with exogenous biotin before harvest of the mycelium for protein extraction. Roughly 50 µg of protein were loaded onto the polyacrylamide gel. Signals were detected with a Streptavidin-HRP conjugate to visualize biotinylated protein. Ponceau S protein staining was used for loading control. ssi, single spore isolate.
(TIFF)

S17 Fig Biotinylation activity of the SmDOC2-TurboID fusion protein. For SmDOC2 BioID experiments, the TurboID ligase was fused to the C-terminus of the SmDOC2 protein via a GGGGSGGGGS linker. This *Smdoc2-TurboID* fusion construct was either ectopically integrated into the ΔSmdoc2 mutant (ΔSmdoc2::Smdoc2-TurboID^ect^ ssi 13.5-2) or it was integrated at the native *Smdoc2* locus using the non-homologous end joining-deficient strain Δku80 (ku80::Smdoc2-TurboID ssi 4.1).To show the catalytic activity of the ligase, the strains were supplemented with exogenous biotin before harvest of the mycelium for protein extraction. Roughly 50 µg of protein were loaded onto the polyacrylamide gel. Signals were detected with a streptavidin-HRP conjugate to visualize biotinylated protein. Ponceau S protein staining was used for loading control. The first lane (wild type without biotin supplementation) shows a slightly increased signal in the loading control. The ectopic integration of *Smdoc2-TurboID* in ΔSmdoc2, the *in locus* integration of *Smdoc2-TurboID* at the native *Smdoc2* locus and the ectopic integration of *sci1-TurboID* into Δsci1 exhibit a substantial increase in overall biotinylation upon biotin supplementation, reaching stronger signals than the wild type. ect, ectopic integration.
(TIFF)

S18 Fig Fluorescence microscopy of the BioID control wt::egfp-TurboID^ect^. Fluorescence microscopy of *S. macrospora* wild type (wt) and wt with ectopically integrated *egfp-TurboID* under control of the *Smdoc1* 1 kb 5′ region (wt::egfp-TurboID^ect^). The weak autofluorescence in the untagged wild type, especially in the green channel, results in high noise levels in the background. The pronounced cytoplasmic signal of the EGFP-TurboID fusion protein in the green channel results in a less noisy and darker background. A scale bar (5 µm) is indicated.
(TIFF)

S19 Fig PCR verification of the *mak2* deletion in the BioID control strain Δmak2; Smdoc2::Smdoc2-TurboID. In order to map the MAK2-dependent proteinaceous environment of SmDOC2, we crossed the Smdoc2::Smdoc2-TurboID (*nat*^*R*^) strain with the sterile Δmak2 deletion strain [8]. The Δmak2 deletion strain was generated by replacement of the *mak2* ORF with a resistance cassette (*hyg*^*R*^), which was removed using the FLP/*FRT* recombination to create a marker-less Δmak2 deletion strain [8,93]. Spores were isolated from recombinant perithecia of the cross and the sterile single spore isolates (2, 7, 14, 15, 18 and 20) were tested for the deletion of the *mak2* gene using the primer pair MAK2KO1 and MAK2KO2 from [8]. *nat*^*R*^, nourseothricin resistance cassette expressing the *nourseothricin acetyltransferase* gene from *S. noursei* under control of the constitutive *trpC* promoter from *A. nidulans*. wt, wild type.
(TIFF)

S20 Fig SmDOC2-TurboID biotinylation activity. For SmDOC2 BioID experiments, the TurboID ligase was fused to the C-terminus of the SmDOC2 protein via a GGGGSGGGGS linker. The *Smdoc2-TurboID* fusion construct was integrated at the native *Smdoc2* locus (Smdoc2::Smdoc2-TurboID ssi 4 and ssi 7). To demonstrate the catalytic activity of the ligase, the strains were supplemented with exogenous biotin before harvest of the mycelium for protein extraction. Roughly 50 µg of protein was loaded onto the polyacrylamide gel. Signals were detected with a Streptavidin-HRP conjugate to visualize biotinylated protein. Ponceau S protein staining was used for loading control.
(TIFF)

S21 Fig Full plates and controls for the SmDOC1/2 Y2H experiment. **(A, B)** Negative controls for the interaction studies of SmDOC1 with MEK2-t1, MEK2-t2, HAM5 and MAK2. **(A)** Y2H analysis with empty pGADT7 vectors and **(B)** Y2H analysis with empty pGBKT7 vectors. **(C)** Positive controls using the AD-ranBPM control [56], which directly binds to the Gal4 binding domain of the BD-bait fusion, thereby confirming expression and competency of the BD-SmDOC1/BD-MEK2-t1/BD-MEK2-t2/BD-HAM5/BD-MAK2 fusions. **(D)** Positive controls of the AD-ranBPM × BD-SmDOC2 mating, testing different clones All four clones with BD-SmDOC2 showing drastically reduced viability when compared to the AD-ranBPM × BD-SmDOC1 interaction.
(TIFF)

S22 Fig SmDOC1-TagRFP-T localizes to multiple septa. Fluorescence microscopy of SmDOC1 tagged with TagRFP-T. **(A)** *In locus* integration of *Smdoc1-TagRFP-T* at the *Smdoc1* locus using the native 5′ region for expression regulation. Every septum in the focal plane shows a weak red fluorescent signal at the central region, which was not observed in the untagged wild-type control. **(B)** Fluorescent signal of the untagged *S. macrospora* wild type, which does not express any fluorescent marker protein. Arrows indicate septa within the focal plane. DIC, differential interference contrast, scale bars are indicated.
(TIFF)

S23 Fig Localization studies of SmDOC1-TagRFP-T. Fluorescence microscopy of SmDOC1 tagged with TagRFP-T. **(A)** Fluorescent signal of the untagged *S. macrospora* wild type, which does not express any fluorescent marker protein. **(B)** *In locus* integration of *Smdoc1-TagRFP-T* at the *Smdoc1* locus using the native 5′ region for expression regulation. **(C)** Expression of *Smdoc1-TagRFP-T* under control of the constitutive promoter of the *clock-controlled gene 1* (*ccg1*) from *N. crassa* integrated into the *S. macrospora* Δku80 strain, which is used for homologous recombination due to its impaired non-homologous end joining pathway. The fluorescent signal in the red channel did not increase after swapping the native *Smdoc1* 5′ region with the constitutive *ccg1* promoter. White arrows indicate septa. A scale bar (5 µm) is indicated. ect, ectopic integration.
(TIFF)

S24 Fig *In locus* tagging of *Smdoc2* with *egfp* and *TagRFP-T.* **(A)** Genomic situation of the *Smdoc2* (*SMAC_06902*) locus in the wild type and after the integration of *Smdoc2-TurboID*. Expression is regulated by the native *Smdoc2* 5′ region and the *TtrpC* terminator of the *anthranilate synthase* gene of *A. nidulans* was used for transcription termination. EGFP is fused to the C-terminus of SmDOC2 via a GGGGS linker. The amplicons of the PCR verification reaction are indicated by black lines. The integration of the fusion at the *Smdoc2* locus was verified using the PCR primer pair LH20/LH21 (3,782 bp) in the single spore isolates 2.1, 2.2, 2.3. **(B)** Southern hybridization verification of Smdoc2::Smdoc2-EGFP. The probe is indicated by a red line and the *EcoR*I restriction enzyme sites are marked by arrows. The primary transformant (PT) 2 is a heterokaryon and exhibits signals for the wild-type locus and the transformed locus. **(C)** *TagRFP-T* tagging of the *Smdoc2* gene was performed analogously to the strain construction of Smdoc2::Smdoc2-EGFP. Primer pair LH20/tRFP-f (2,910 bp) was used to verify the *in locus* integration in the single spore isolate 1.1, 1.2 and 2.1. *nat*^*R*^, nourseothricin resistance cassette expressing the *nourseothricin acetyltransferase* gene from *S. noursei* under control of the constitutive *trpC* promoter from *A. nidulans*. PT, primary transformant.
(TIFF)

S25 Fig Fluorescence microscopy of SmDOC2-EGFP and SmDOC2-TagRFP-T. Fluorescence microscopy of *Smdoc2* tagged with **(A)** *egfp* and **(B)** *TagRFP-T*. **(A)** *In locus* integration of *Smdoc2-egfp* at the *Smdoc2* locus using the native 5′ region for expression regulation. The construct was transformed into the *S. macrospora* Δku80 strain, which is used for homologous recombination due to its impaired non-homologous end joining pathway. The strain was fully verified for the *in locus* integration of *Smdoc2-egfp* and the absence of the wild-type *Smdoc2* gene by PCR and Southern hybridization. The wild-type *ku80* gene was restored by crossing with the color spore mutant fus 1-1 expressing the wild-type *ku80* gene. The fluorescent signal of SmDOC2-EGFP did not surpass the intensity of the autofluorescence observed in the untagged *S. macrospora* wild type. The exchange of the 5′ region with the constitutive promoter of the *clock-controlled gene 1* (*ccg1*) from *N. crassa* did not result in any changes of fluorescent signal output. **(B)** Since the autofluorescence of the untagged *S. macrospora* wild type is less pronounced in the red channel during fluorescence microscopy, we fused *Smdoc2* to the red fluorescent protein *TagRFP-T*. Expression of this *Smdoc2-TagRFP-T* fusion is either controlled by the 1 kb 5′ *Smdoc2* region or the constitutive *ccg1* promoter. However, no fluorescent signal was observed for SmDOC2-TagRFP-T using either promoter for expression regulation. A scale bar (5 µm) is indicated. ect, ectopic integration.
(TIFF)

S1 Data Significantly enriched proteins in SCI1-TurboID samples compared to the free TurboID controls. (XLSX)

S2 Data All proteins from the SCI1-BioID volcano plot (Fig 1B) including their imputed intensities. (XLSX)

S3 Data Significantly enriched proteins in SCI1-TurboID samples compared to the Δpro11 controls. (XLSX)

S4 Data All proteins from the SCI1-BIoID (Δpro11 control) volcano plot (Fig 1C) including their imputed intensities. (XLSX)

S5 Data Whole proteome analysis (input control) of Δpro11Δsci1::SCI1-TurboID^ect^ and Δsci1::SCI1-TurboID^ect^. (XLSX)

S6 Data Selected protein intensities of the proteome analysis of Δpro11Δsci1::sci1-TurboID^ect^ and Δsci1::sci1-TurboID^ect^. (XLSX)

S7 Data Total vegetative growth of *Smdoc* knockouts 72 h after inoculation. (XLSX)

S8 Data Vegetative growth rates of *Smdoc* knockouts after colony establishment. (XLSX)

S9 Data Quantification of ejected ascospores of Smdoc knockouts. (XLSX)

S10 Data Significantly enriched proteins in SmDOC1-TurboID samples compared to the free TurboID controls. (XLSX)

S11 Data All proteins from the SmDOC1-BIoID volcano plot (Fig 4B) including their imputed intensities. (XLSX)

S12 Data Significantly enriched proteins in SmDOC2-TurboID samples compared to the free TurboID controls. (XLSX)

S13 Data All proteins from the SmDOC2-BioID volcano plot (Fig 4C) including their imputed intensities. (XLSX)

S14 Data Significantly enriched proteins in Δmak2;Smdoc2::Smdoc2-TurboID samples compared to the free TurboID controls. (XLSX)

S15 Data All proteins from theΔmak2-SmDOC2-BioID volcano plot (Fig 5) including their imputed intensities. (XLSX)

S16 Data MEK2 peptides (SMAC_06526) found in SmDOC1-BioID samples. (XLSX)

S17 Data MEK2 peptides (SMAC_06526) found in SmDOC2-BioID samples. (XLSX)

S18 Data Raw file naming scheme. (XLSX)

## Abbreviations

AD: activation domain
BD: binding domain
BioID: Biotin Identification
BMM: biomalt maize medium
DOC: determinant of communication
ERKs: extracellular signal-regulated kinases
GZMB: Göttingen Center for Molecular Biosciences
LFQ: Label-Free Quantification
MAP: mitogen-activated protein
OD: optical density
ORFs: open reading frames
PAGE: polyacrylamide gel electrophoresis
PR: pheromone response
SCI1: STRIPAK complex interactor 1
SIN: septation initiation network
SWG: *Sordaria* Westergaard’s
STRIPAK: striatin-interacting phosphatase and kinase
TAP: tandem affinity purification
