# Supplementary material for: STRIPAK-associated greenbeard proteins DOC1 and DOC2 regulate MAK2 signaling and sexual development in Sordaria macrospora
Source: PLoS Biol. 2026 Aug 27;24(8):e3003969. doi: 10.1371/journal.pbio.3003969 (PMC13557503; doi:10.1371/journal.pbio.3003969)

S3 Fig. Verification of the BioID control strain Δpro11Δsci1::sci1-TurboID^ect.

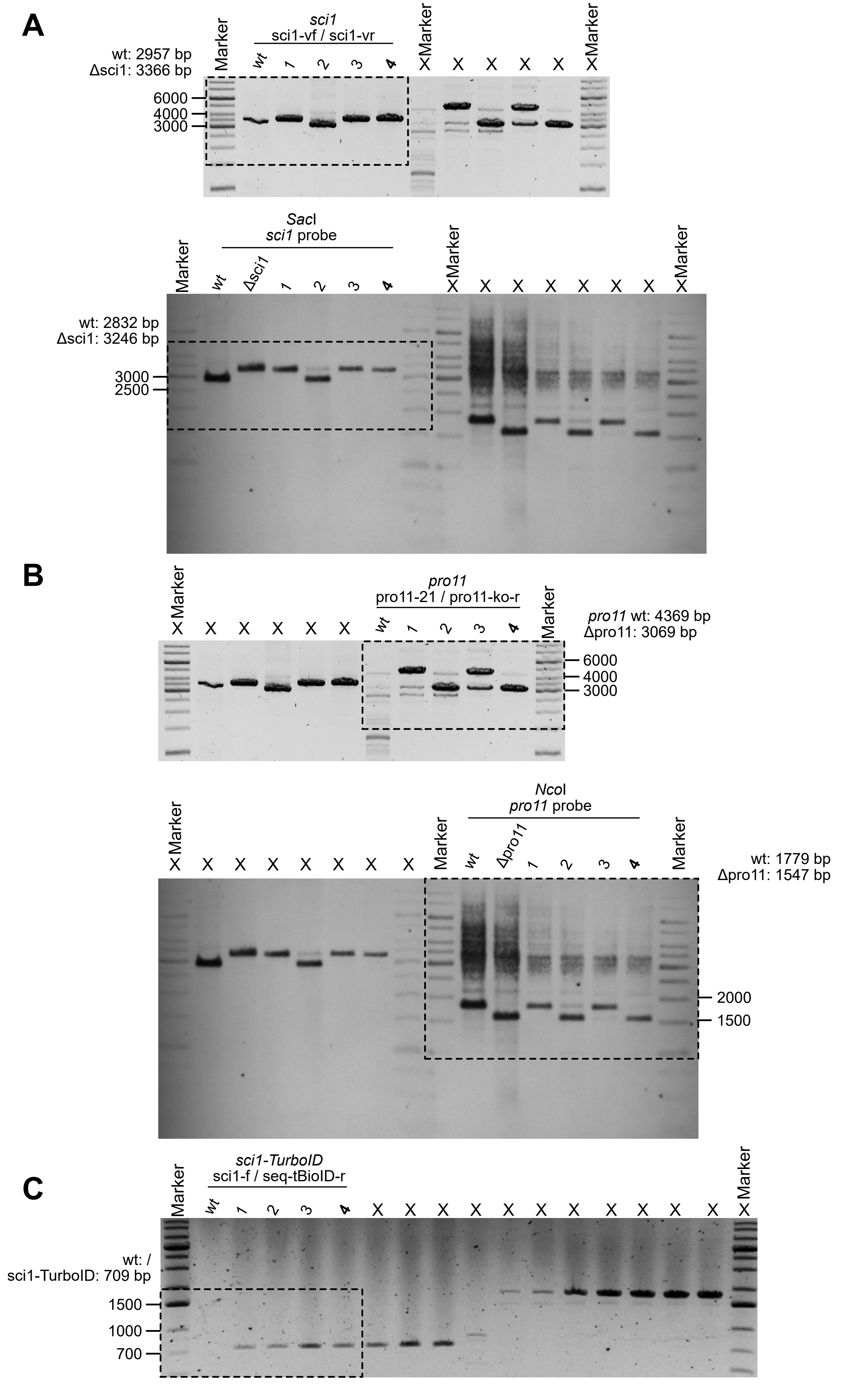

S6A Fig. Generation and verification of the ΔSmdoc1 deletion strain.
raw data panel A)

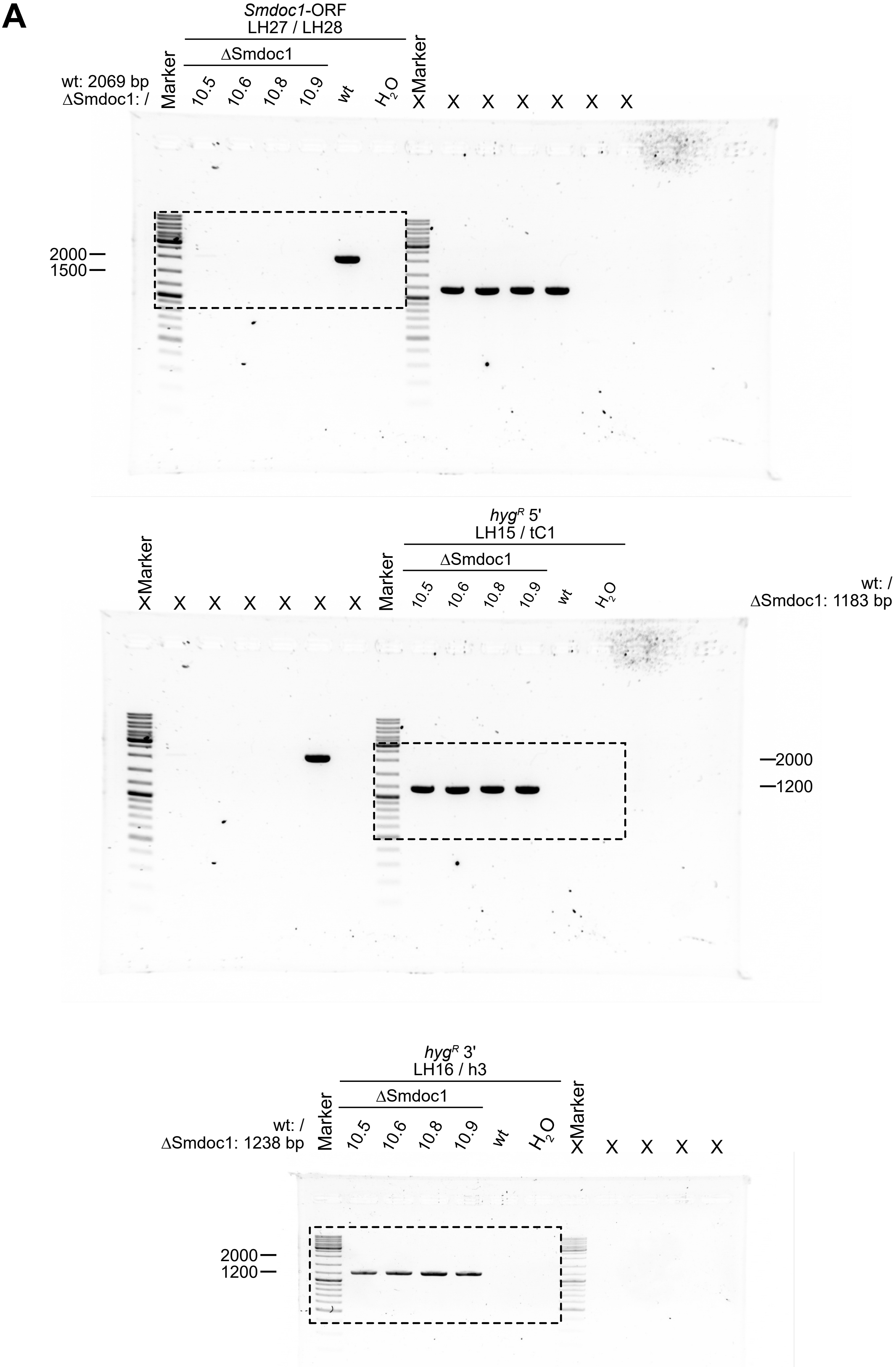

S6 Fig. Generation and verification of the ΔSmdoc1 deletion strain. raw data panel B)

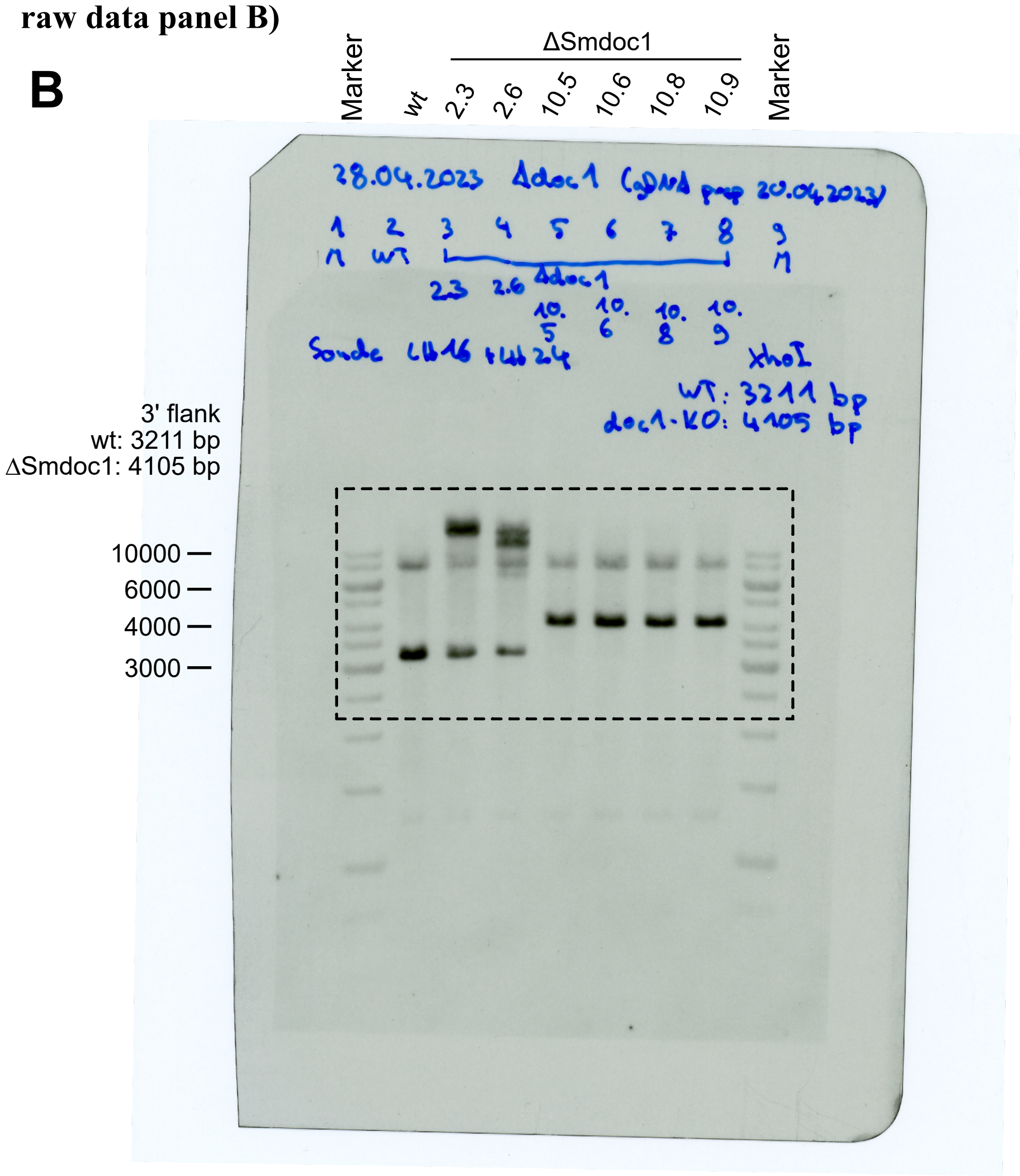

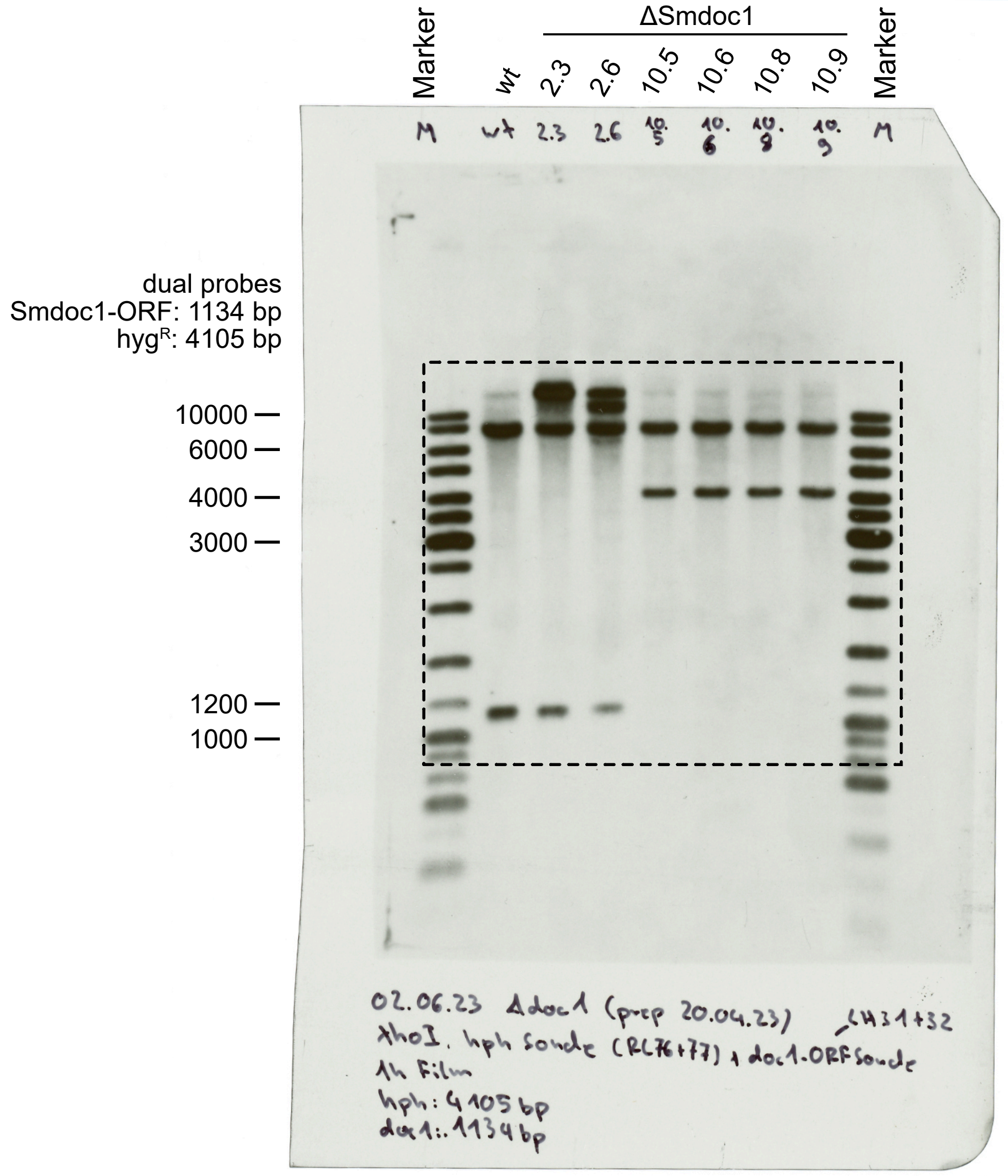

S7 Fig. Generation and verification of the ΔSmdoc2 deletion strain.
raw data panel A)

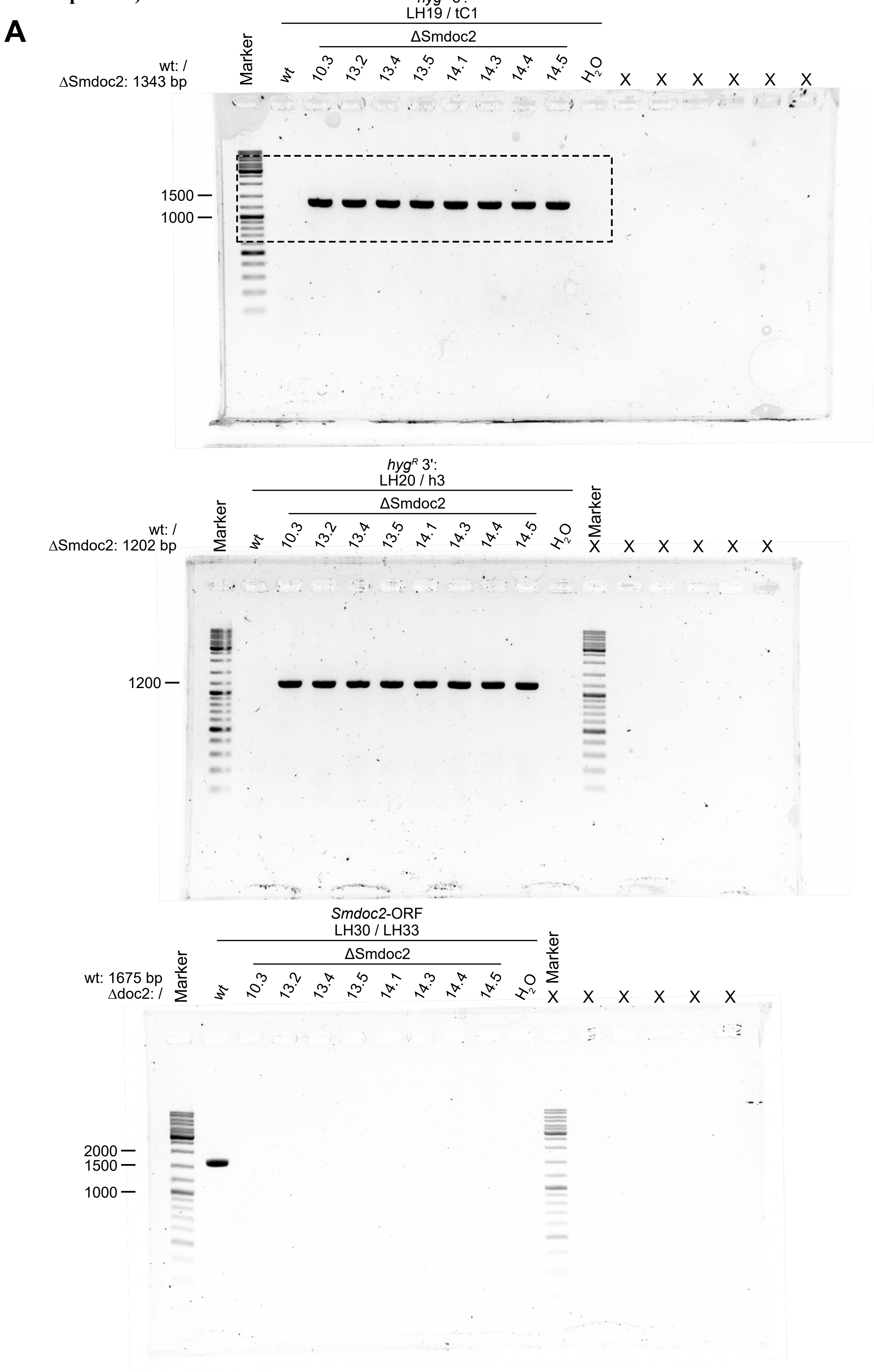

S7 Fig. Generation and verification of the ΔSmdoc2 deletion strain.
raw data panel B)

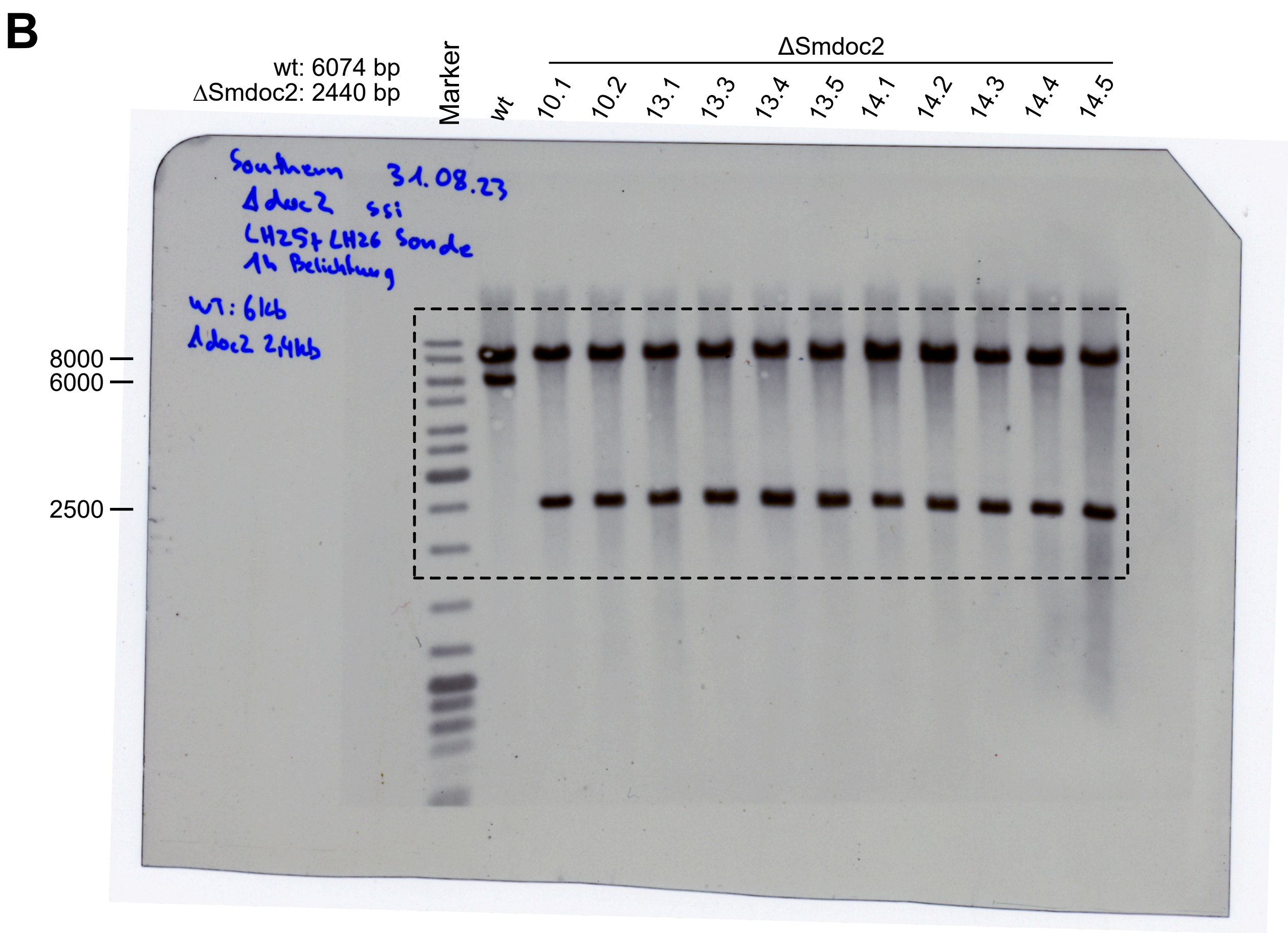

S8 Fig. Generation and verification of the ΔSmdoc1ΔSmdoc2 double knockout.
raw data panel A)

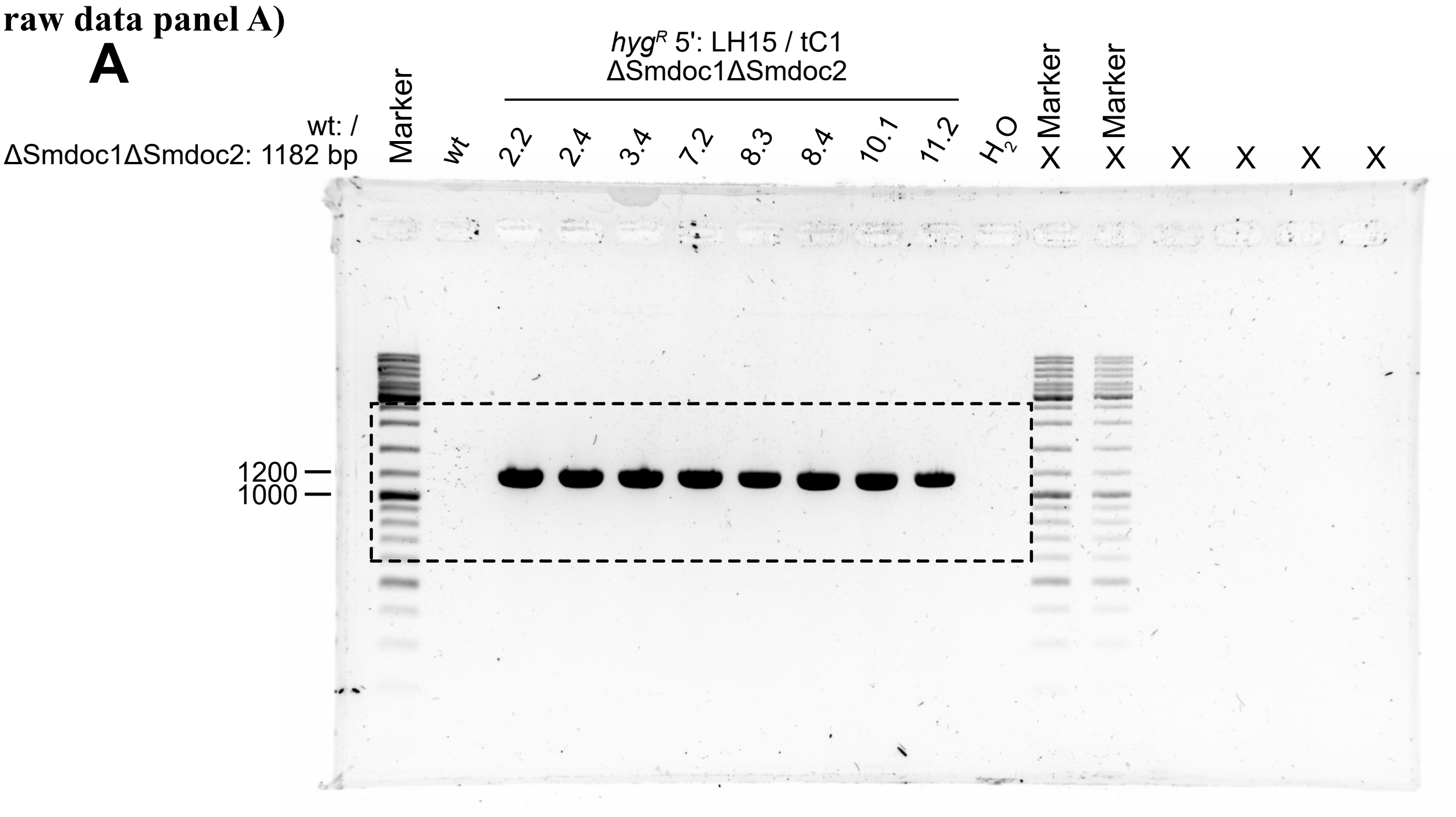

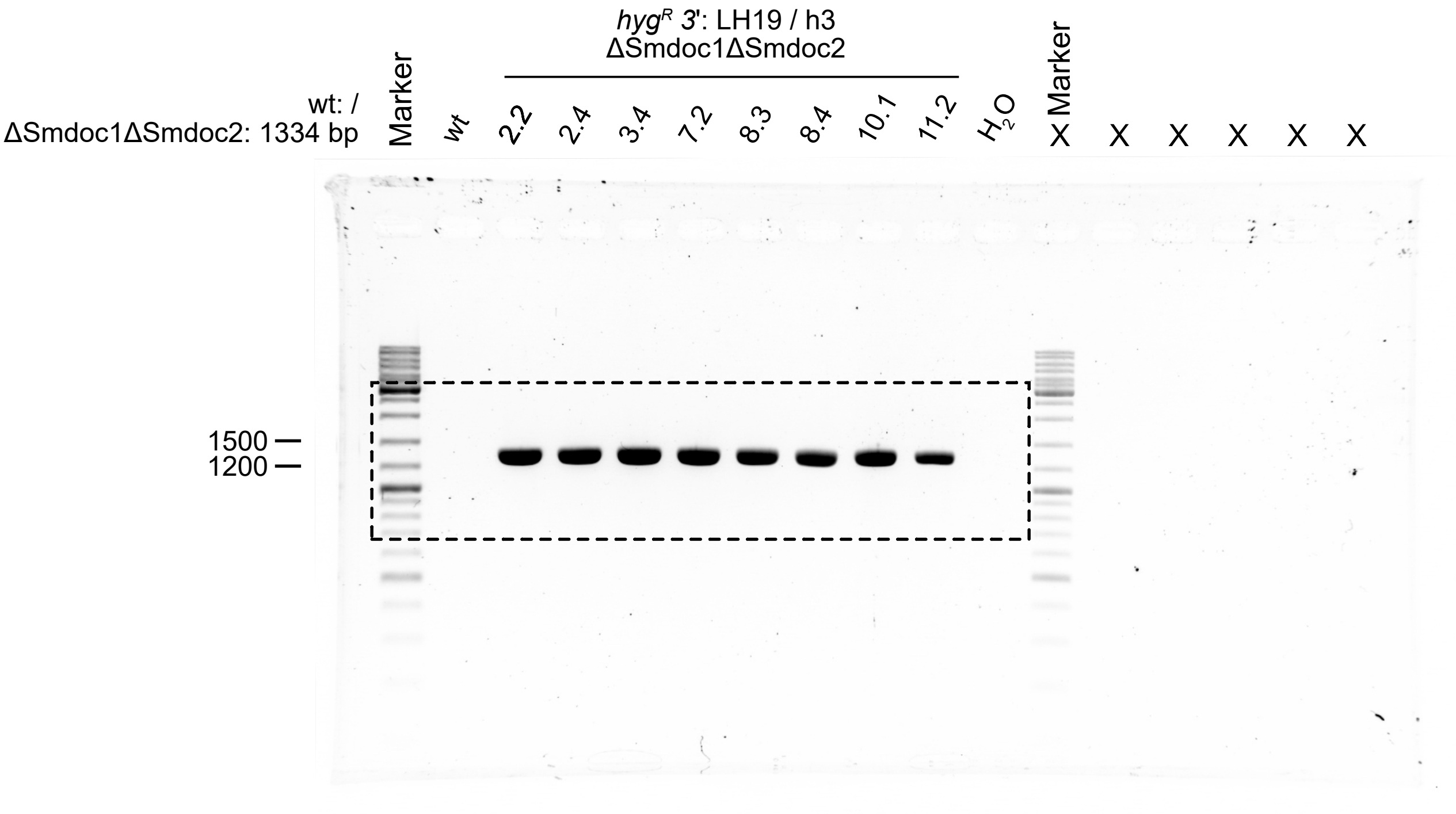

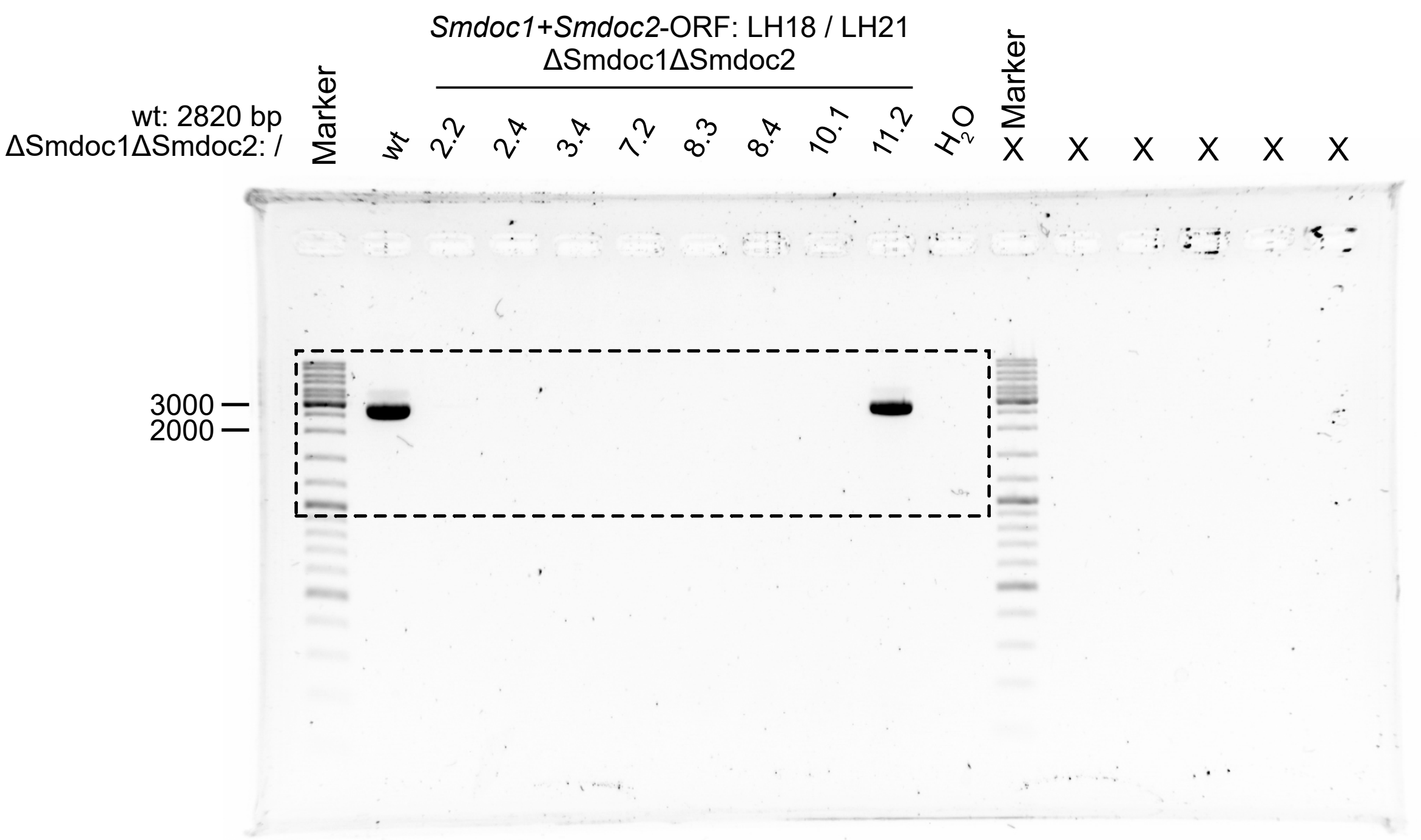

S8 Fig. Generation and verification of the ΔSmdoc1ΔSmdoc2 double knockout. raw data panel A)

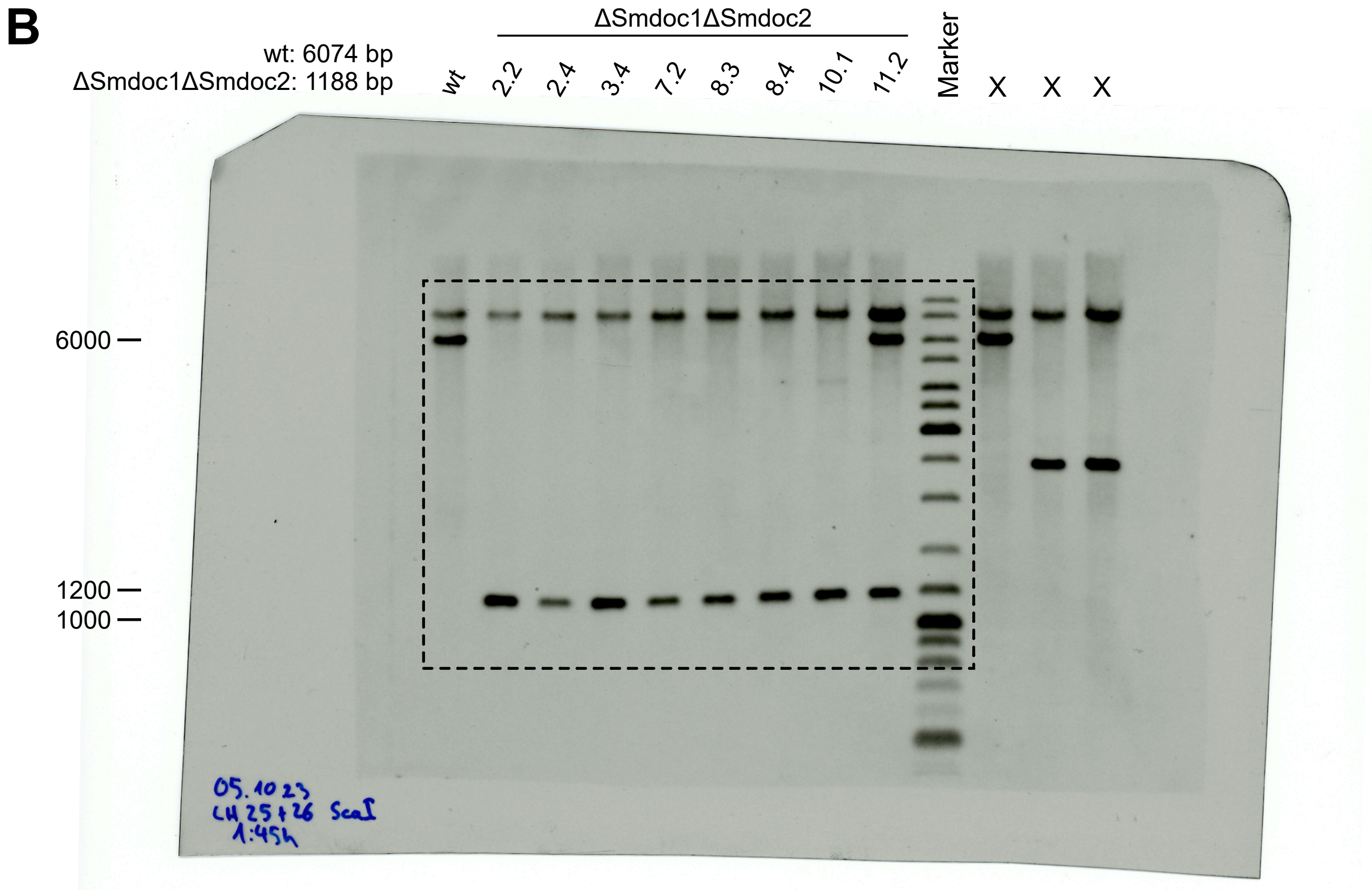

S14 Fig. In locus tagging of Smdoc1 with TurboID and TagRFP-T.

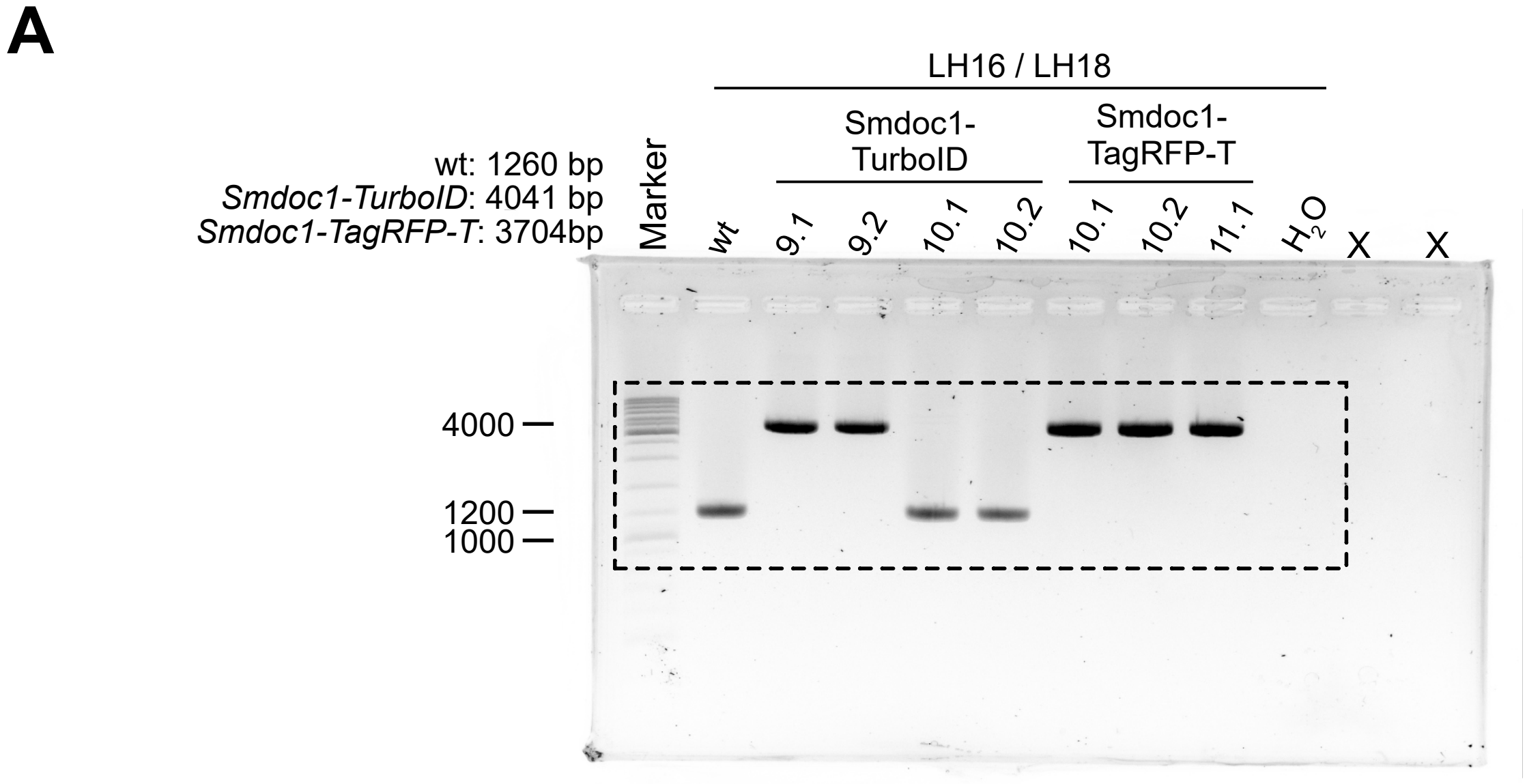

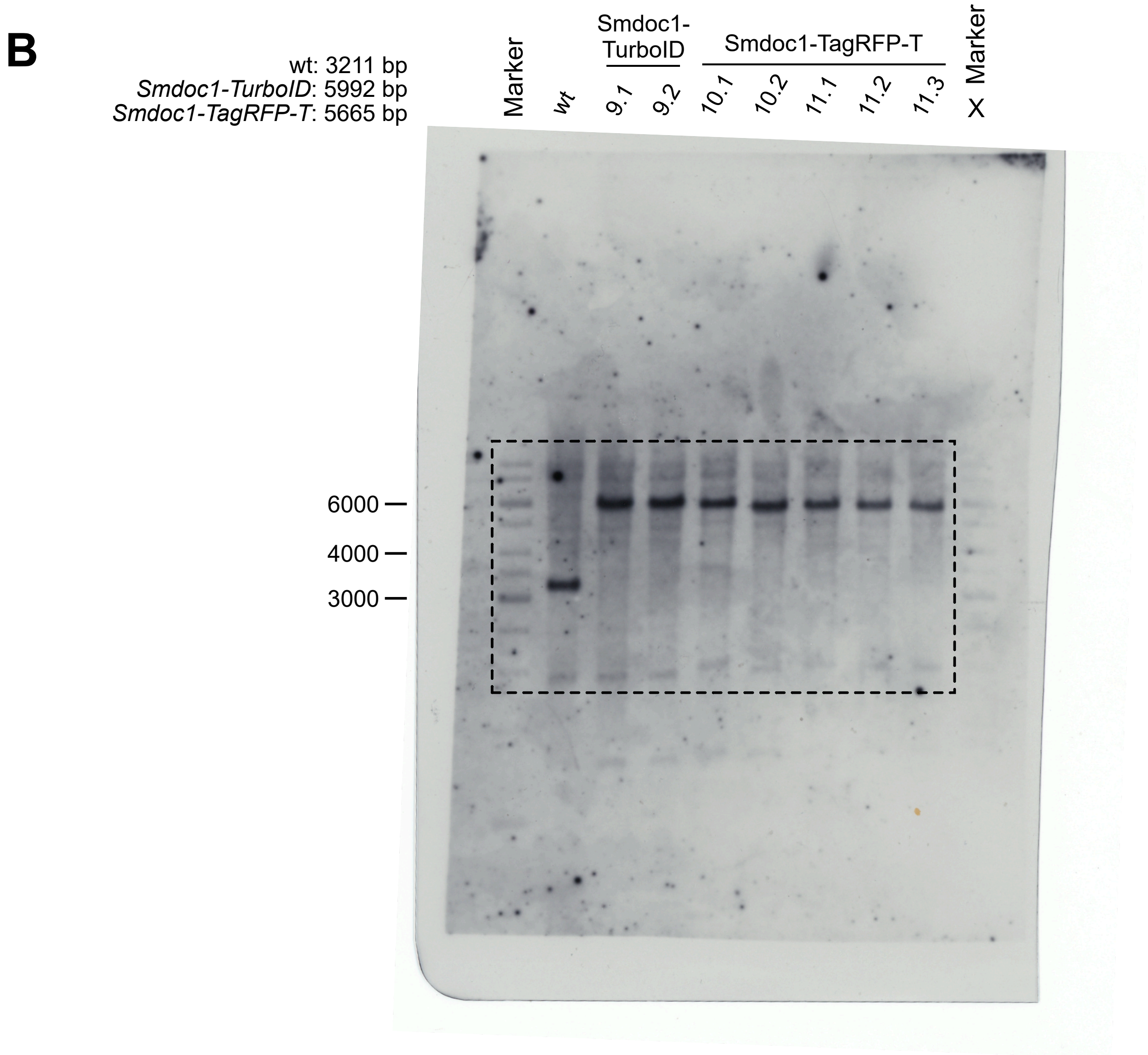

S15 Fig. In locus tagging of Smdoc2 with TurboID.

A

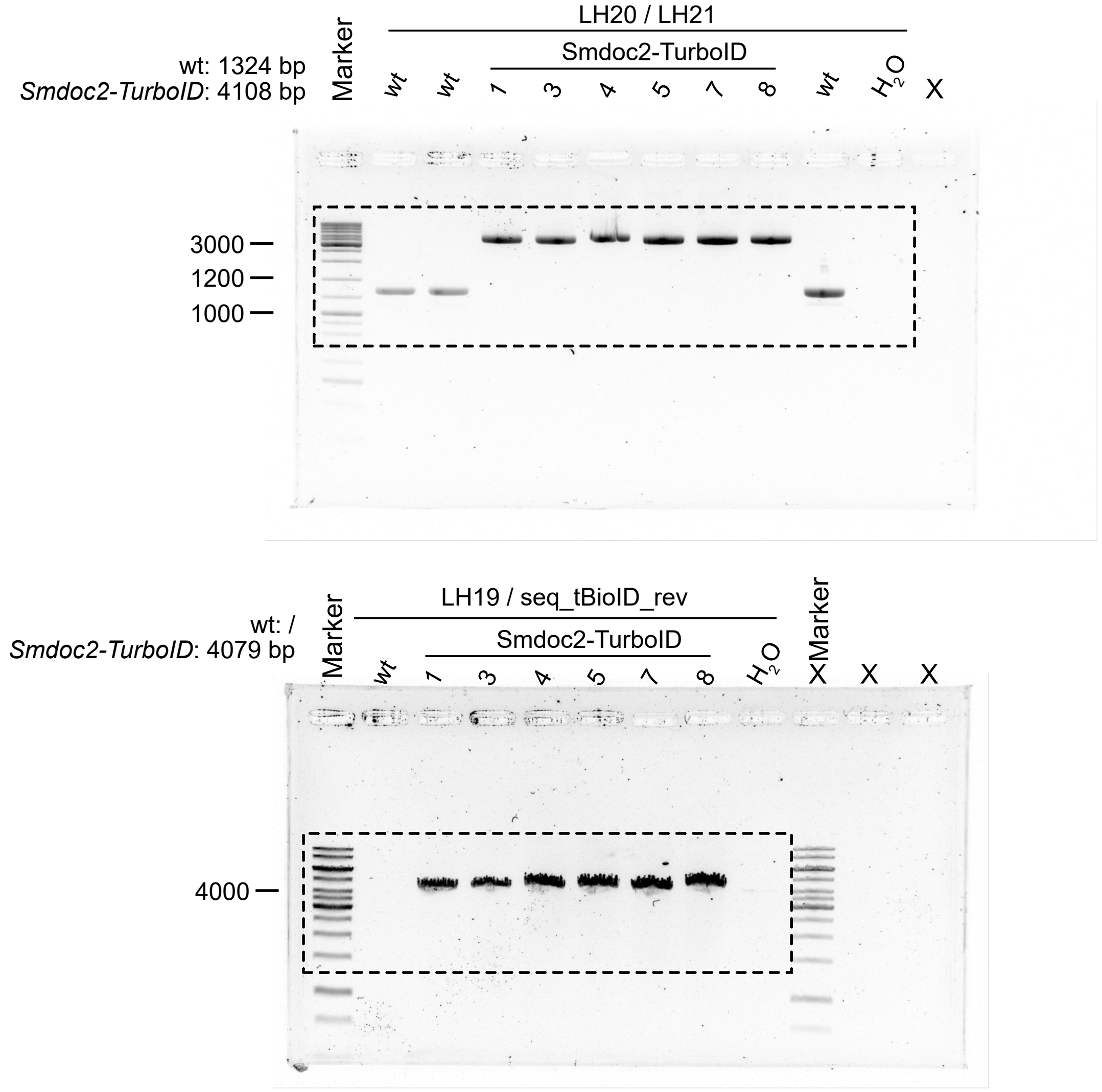

B

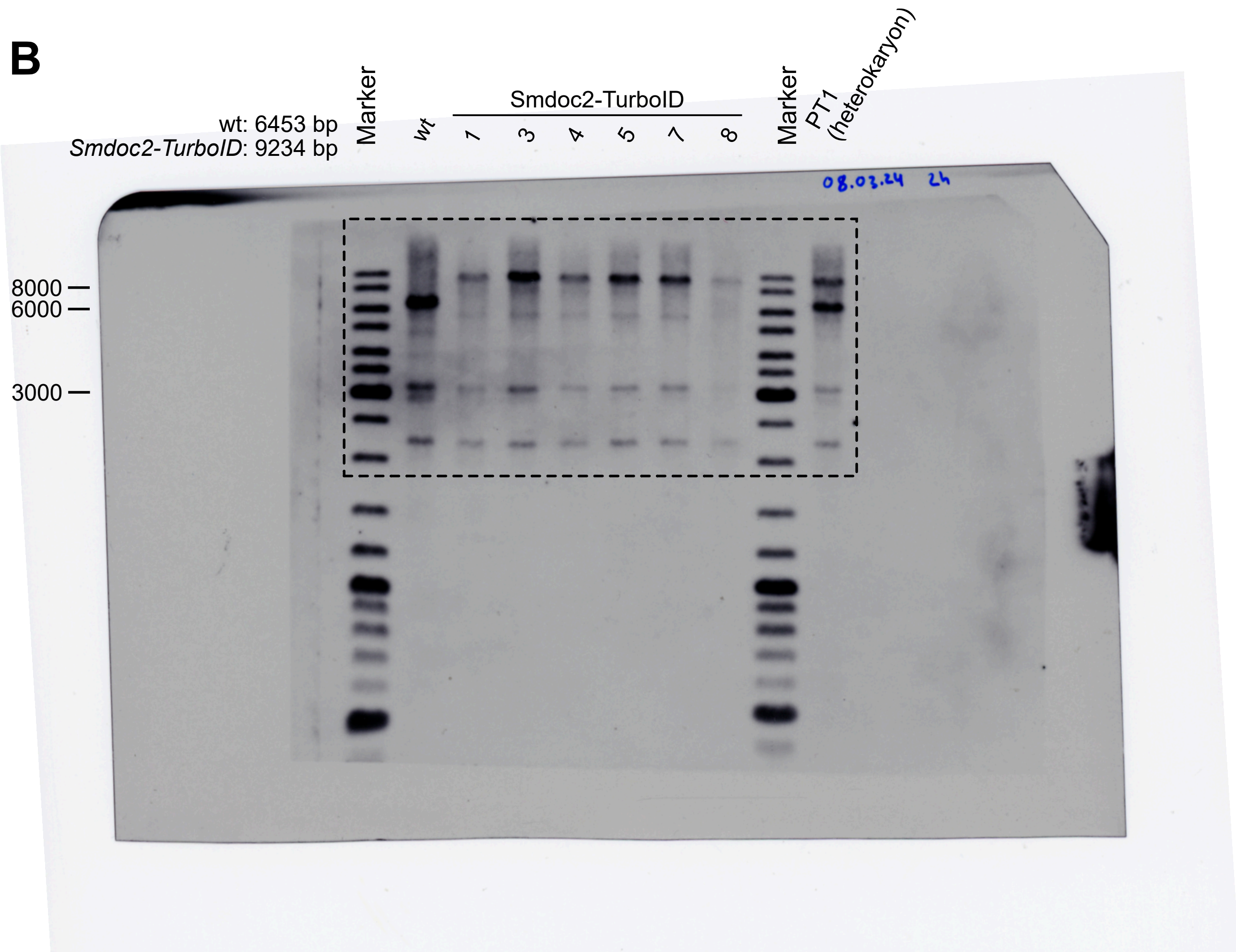

S16 Fig. Biotinylation activity of the EGFP-TurboID control and the SmDOC1-TurboID fusion protein.

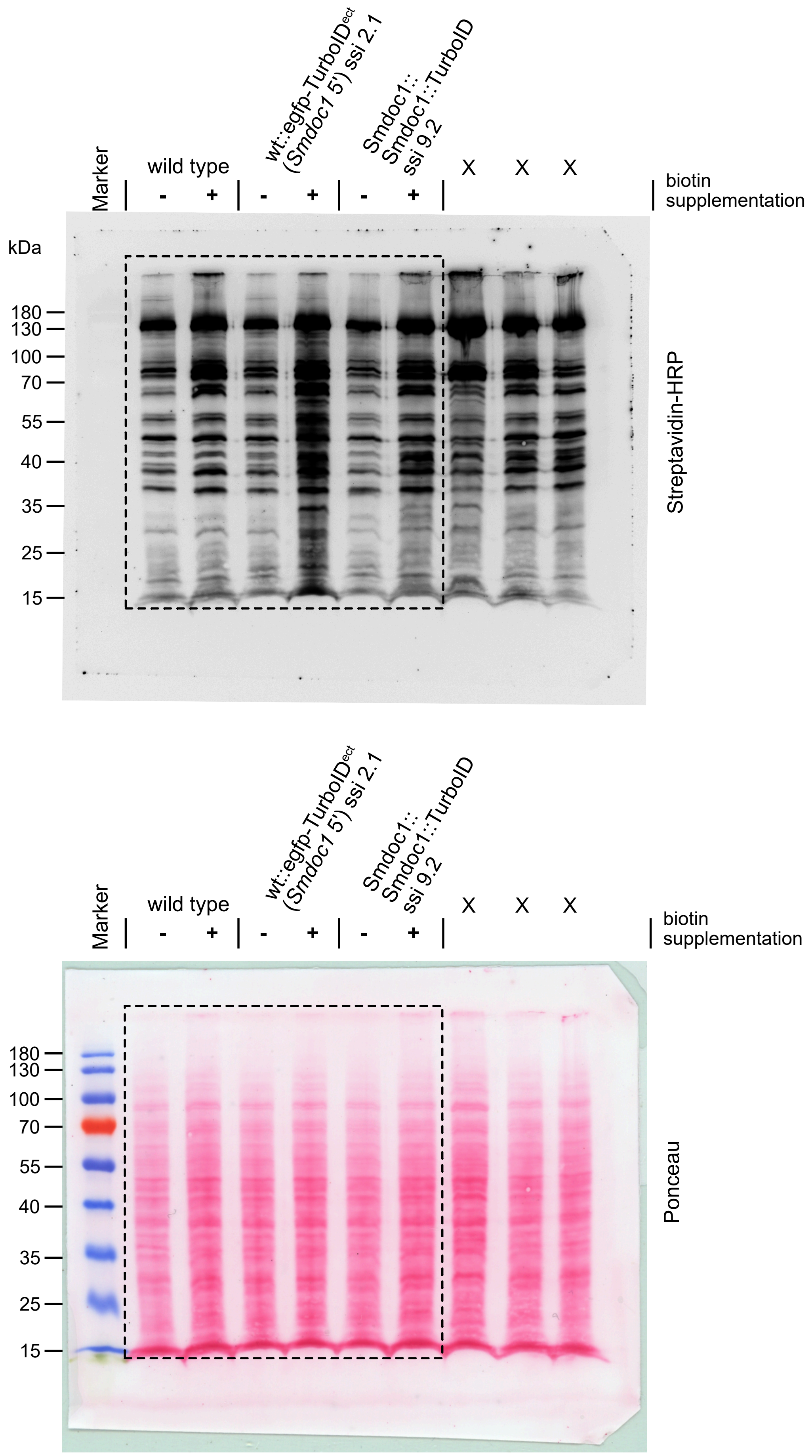

S17 Fig. Biotinylation activity of the SmDOC2-TurboID fusion protein.

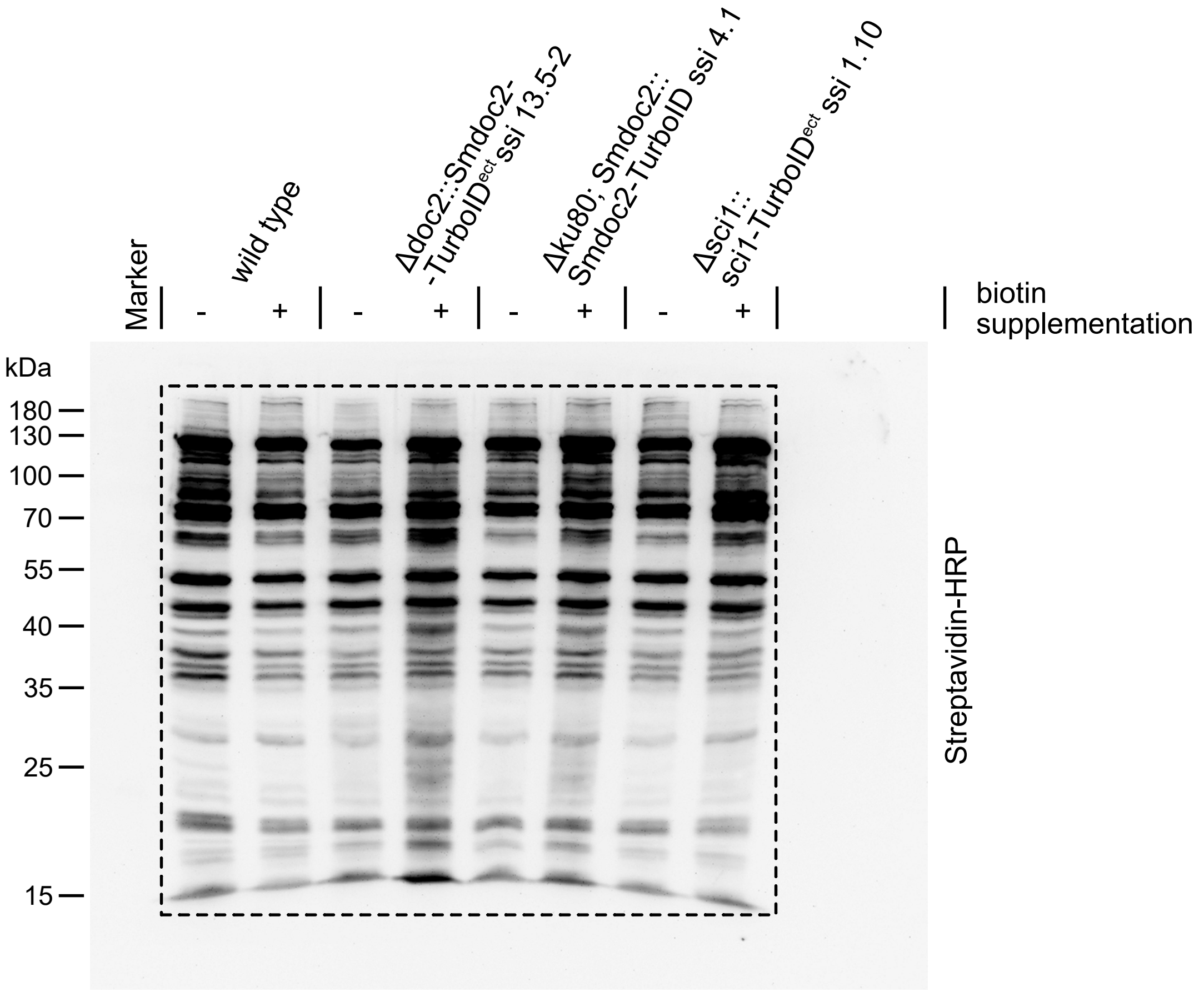

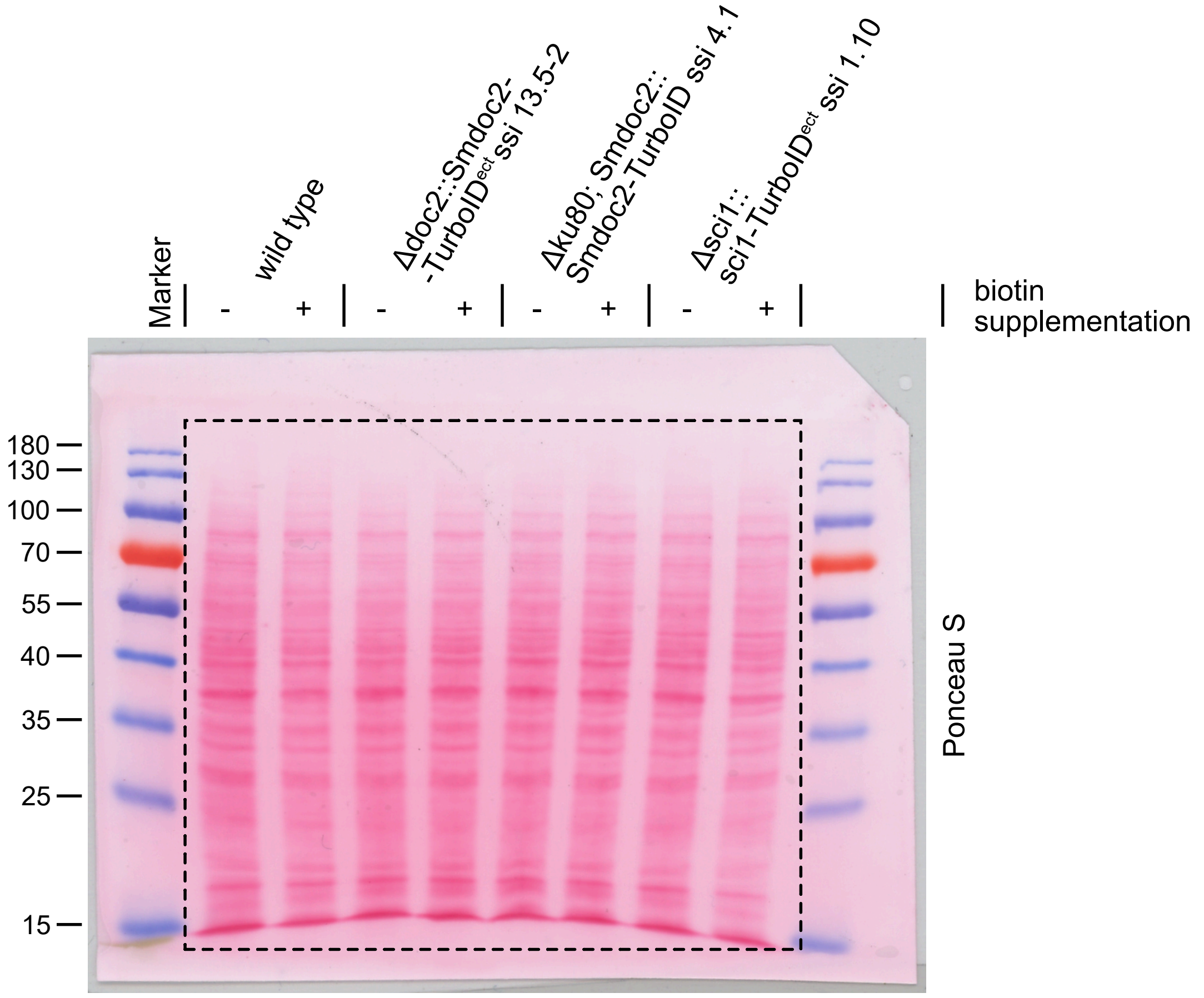

S19 Fig. PCR verification of the mak2 deletion in the BioID control strain Δmak2; Smdoc2::Smdoc2-TurboID.

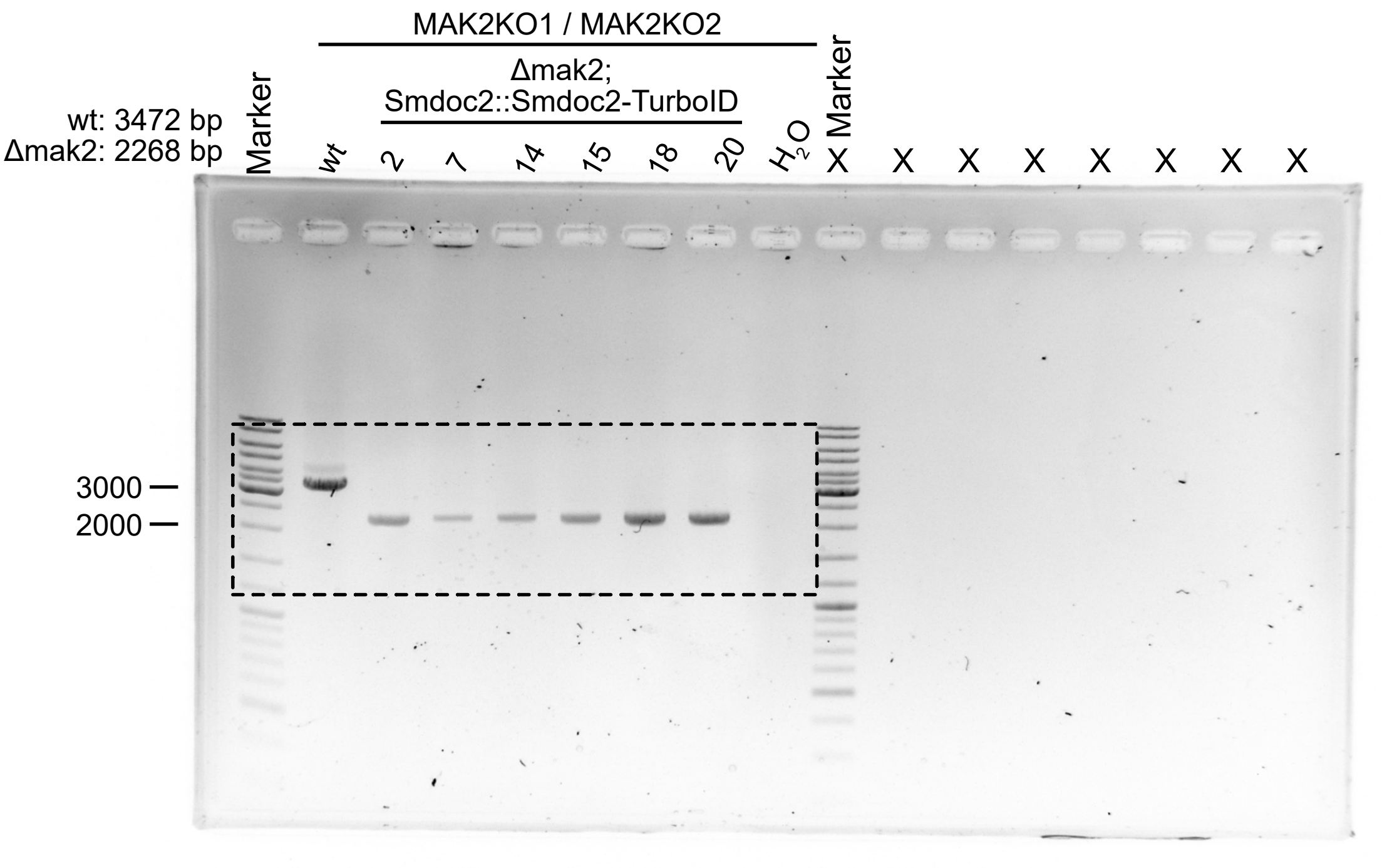

S20 Fig. SmDOC2-TurboID biotinylation activity.

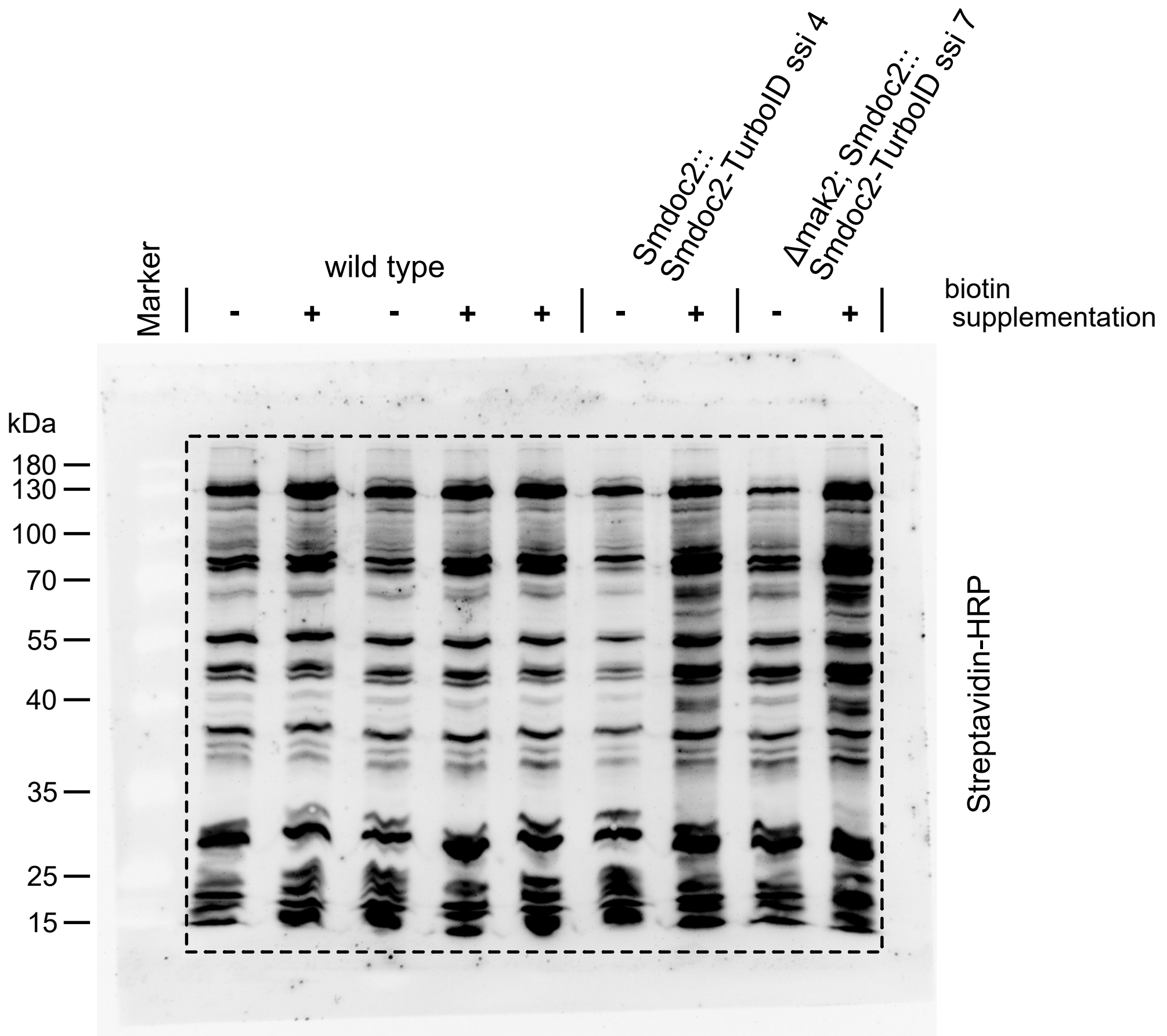

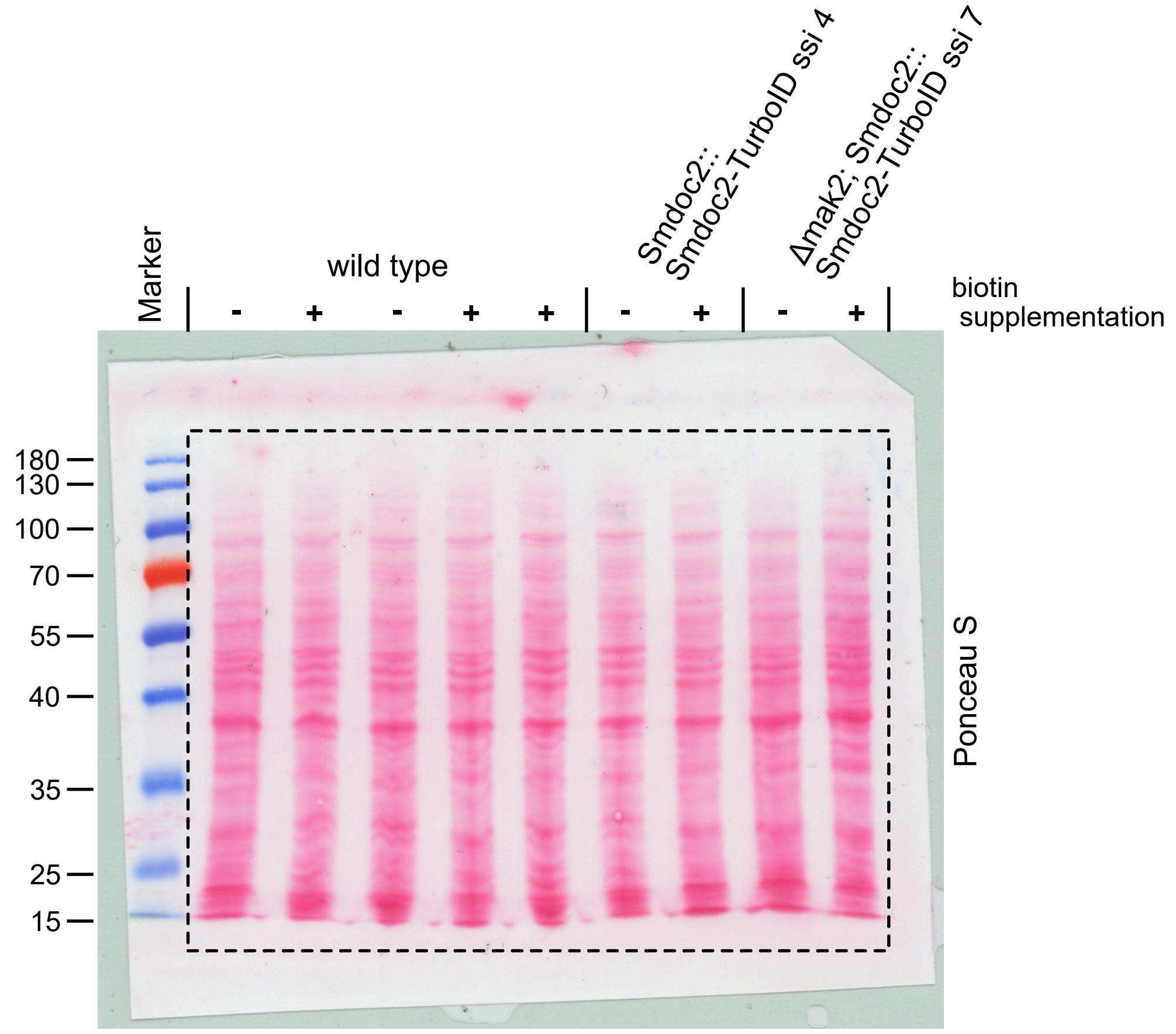

S24 Fig. In locus tagging of Smdoc2 with egfp and TagRFP-T.
raw data panels A) + B)

A

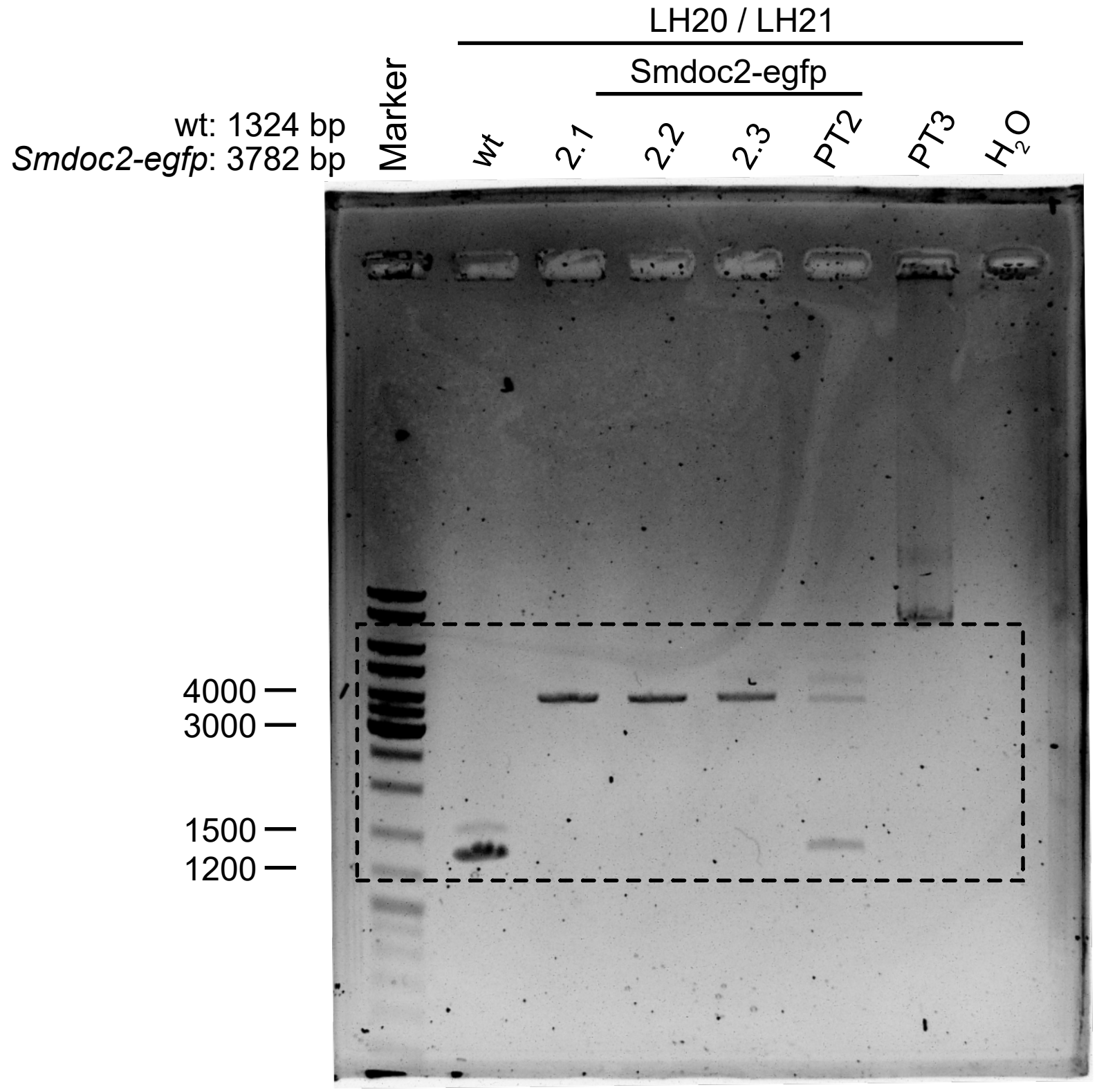

B

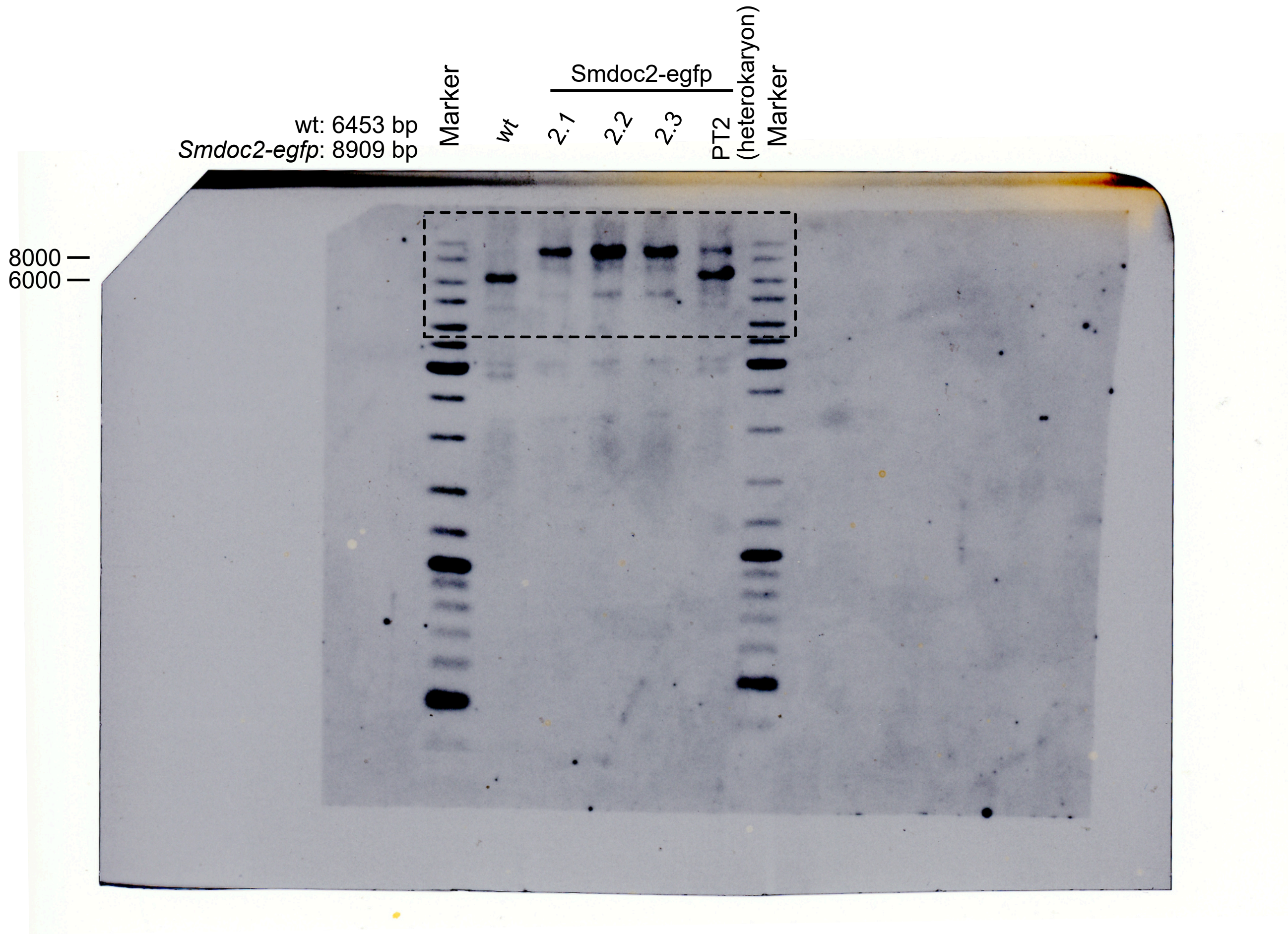

S24 Fig. In locus tagging of Smdoc2 with egfp and TagRFP-T.
raw data panel C

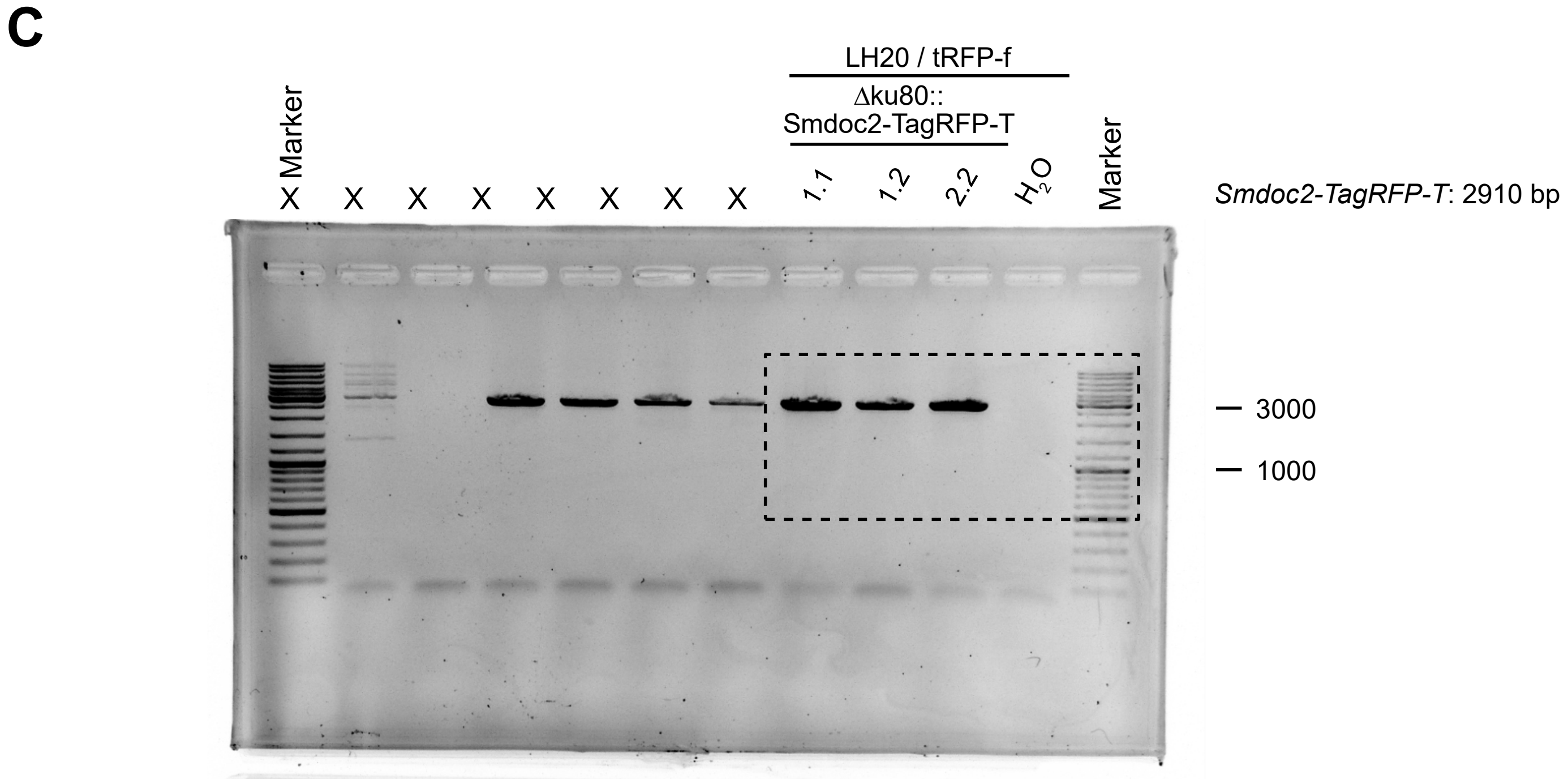

Supplement: Supplementary file 1 [file pbio.3003969.s001.pdf]
