# Supplementary material for: STRIPAK-associated greenbeard proteins DOC1 and DOC2 regulate MAK2 signaling and sexual development in Sordaria macrospora
Source: PLoS Biol. 2026 Aug 27;24(8):e3003969. doi: 10.1371/journal.pbio.3003969 (PMC13557503; doi:10.1371/journal.pbio.3003969)
Supplement: Supplementary file 3 [file pbio.3003969.s050.pdf]

Point by Point Responses to the reviewers

Editor:

As you will see in the reports, reviewers #2 and #3 are now broadly satisfied with the revised manuscript, and reviewer #1 also acknowledges the substantial improvements in clarity, balance, controls, and quantification. However, reviewer #1 remains concerned that key aspects of the proposed mechanistic model are still speculative and that the localization data do not yet provide sufficiently strong support for the conclusions drawn. In addition, reviewer #2 raises related concerns about the interpretation of SmDOC1 localization, particularly the description of a “ring-like” structure based on 2D images, and asks that the apparent septal-pore localization be discussed more directly in relation to its possible biological function in sexual development. After discussion with the Academic Editor, we are therefore inviting a light major revision focused on these remaining issues. While we will not require further experiments for mechanistic insights, we do ask that you strengthen and further qualify the SmDOC1 localization data, and ensure that any mechanistic conclusions are appropriately supported by the data or explicitly framed as speculative. Please also address reviewer #2’s remaining textual, citation, and clarification points.

Response: We sincerely thank the Editor and the Academic Editor for their careful evaluation of our revised manuscript and for the opportunity to address the remaining concerns. We are grateful that the reviewers recognize the substantial improvements made in the previous revision and appreciate their constructive suggestions for further strengthening the manuscript. We are encouraged by the reviewers’ overall positive assessment of the relevance and novelty of our study. Accordingly, we have revised the relevant sections throughout the manuscript to clearly distinguish observations supported by our data from mechanistic interpretations. Specifically, we have softened the language describing the proposed model by explicitly stating that it represents a working hypothesis based on our findings and current knowledge of fungal developmental regulation. Statements that previously implied mechanistic certainty have been revised to emphasize their speculative nature, and the Discussion now more clearly outlines the limitations of our study and identifies future experiments that will be required to test these hypotheses.

Following the Editor's recommendation, we have substantially revised the description of the SmDOC1 localization results. The presentation now focuses on the experimentally observed fluorescence distribution without overinterpretation. We have clarified the limitations inherent to the imaging approach and carefully qualified our conclusions throughout the manuscript. These revisions provide a more balanced interpretation of the localization data while preserving the primary observations supported by the experiments.

Reviewer #1:

I appreciate the effort the authors put into revising the manuscript. They have addressed the specific concerns raised by the reviewers, added important controls and quantification, clarified several aspects of the study, and generally made the manuscript more balanced and easier to follow. I also appreciate that the authors have substantially tempered a number of their conclusions and now more clearly distinguish between observations supported by the data and the more speculative aspects of the proposed model.

Response: We sincerely thank the reviewer for their thoughtful reassessment of our manuscript and for recognizing the substantial improvements made during the previous revision. We greatly appreciate the positive evaluation of the additional controls, quantitative analyses, and clarifications, as well as the acknowledgment that the manuscript now presents a more balanced interpretation of the data. We are pleased that our efforts to clearly distinguish experimentally supported observations from speculative aspects of the proposed model have addressed many of the reviewer's previous concerns. We have carefully considered the remaining comments and have further revised the manuscript to strengthen the presentation of the localization data, appropriately qualify mechanistic interpretations, and improve the clarity of the discussion, as detailed in our responses below.

With that said, my overall assessment regarding the suitability for PLOS Biology is unchanged. While the revised manuscript presents an interesting set of genetic, proteomic, and interaction data linking DOC proteins, STRIPAK, and the pheromone response MAPK pathway, the central mechanistic model is still largely speculative. The revisions mainly improve interpretation of the existing data rather than providing new evidence that directly establishes how these components function together. Although the authors now appropriately acknowledge that key aspects of the proposed model remain hypothetical and are supported largely by proximity and genetic data, the revision does not substantially advance the mechanistic understanding of how DOC1/2 regulate MAPK signaling or explain the suppression phenotype observed in the double mutant.

Response: We thank the reviewer for the recognition that the revised manuscript presents an interesting genetic, proteomic, and interaction dataset and that our previous revision appropriately distinguished experimentally supported observations from speculative interpretations. We agree that our current data do not directly establish the molecular mechanism by which DOC1/2 regulate MAPK signaling or explain the suppression phenotype observed in the double mutant. As also reflected in the Editor's decision letter, the present revision was intended to focus on clarifying the interpretation of the existing data rather than providing additional mechanistic experiments. In response to this remaining concern, we have further revised the manuscript to ensure that all mechanistic interpretations are explicitly presented as working hypotheses rather than established conclusions. We have carefully moderated the wording throughout the Results, and Discussion, emphasizing that the proposed model is based on the combined genetic, proteomic, interaction, and localization data, while acknowledging that direct experimental validation of the underlying molecular mechanisms will require future studies. We have also included a dotted line in Fig. 8; this dotted line indicates that the relationship between STRIPAK and SmDOC1/2 is based on a hypothesis

We believe that these revisions provide a more accurate representation of the scope of our findings while preserving the principal conclusions supported by the experimental evidence. We hope that the reviewer agrees that the manuscript now clearly distinguishes between data-driven conclusions and hypotheses intended to guide future mechanistic investigations.

I also remain unconvinced by the localization data. The authors appropriately softened their interpretation, but the signal is weak and difficult to separate from background fluorescence, limiting the conclusions that can be drawn from these experiments. I believe the study presents a valuable dataset and a useful framework for future investigation, but in its current form it remains largely descriptive and hypothesis-generating rather than mechanistic. For this reason, while I consider the work scientifically sound and potentially suitable for a strong journal such as PLOS Genetics, I do not feel that it provides the level of mechanistic insight expected for publication in PLOS Biology.

Response: We agree that the fluorescence signal is relatively weak and that the localization data have inherent limitations. In response to this concern and the Editor's guidance, we have further revised the manuscript to ensure that the localization results are described strictly according to the experimental observations, avoiding interpretations that extend beyond the resolution and quality of the imaging data. We have also further qualified the Discussion to explicitly acknowledge these limitations and to emphasize that the observed localization patterns should be regarded as consistent with, but not definitive evidence for, the proposed functional model.

Regarding the broader concern about mechanistic insight, we have further refined the Discussion to clearly distinguish experimentally supported findings from mechanistic hypotheses. We believe the principal strength of this study lies in integrating complementary genetic, proteomic, interaction, and localization data to identify previously unrecognized connections between DOC proteins, STRIPAK, and the pheromone response MAPK pathway. We hope that, with the additional revisions, the manuscript now presents these findings in a balanced manner that accurately reflects both their significance and their current limitations while providing a solid foundation for future mechanistic studies.

Reviewer #2:

This reviewer appreciates the thoughtful responses by the authors to reviewer's questions and suggestions and the thorough revision of the manuscript. My comments below are minor, with a couple of important questions that can be addressed without additional experimentation.

Response: We sincerely thank the reviewer for the positive assessment of our revised manuscript and for recognizing the thoroughness of our revisions and responses to the previous comments. We greatly appreciate the reviewer's constructive feedback and are pleased that the remaining concerns are primarily minor and can be addressed without additional experimentation. We have carefully considered each of the comments and questions below.

Line 137: hardly to hard.

Response: was changed accordingly

Line 147: reference for ccg-1 and use as a promoter.

Response: we included the reference McNally & Free (1988) and Freitag et al.2004 for the ccg-1 promoter

Line 279: reference for the trpC promoter.

Response: we included the reference Hamer & Timberlake (1987) for the trpC promoter

Line 309: reference for anthranilate synthase terminator in A. nidulans.

Response: we included the reference Mullaney et al. 1985 for the anthranilate synthase terminator of *A. nidulans.*

Line 376: spell out Streptomyces first time it is used

Response: Streptomyces spelled out the first time it is used

Line 428: Not clear why this control sample data was "interesting".

Response: we revised the text and no longer state that the control is interesting

Line 467, plus other sections of the manuscript (Fig. 7 legend; line 583) where localization of SmDOC1 is discussed. Localization of SmDOC1 to a "ring-like structure" is not apparent from the localization data presented, as the images are in 2D.

Response: We thank the reviewer for this important comment and agree that the term "ring-like structure" may overinterpret the localization pattern observed in the two-dimensional fluorescence images. Our intention was to describe the apparent distribution pattern of SmDOC1 fluorescence in the septal region; however, we acknowledge that the available imaging data do not allow us to conclude that SmDOC1 forms a true three-dimensional ring structure.

To address this concern, we have revised the manuscript by replacing the term "ring-like structure" with a more descriptive and cautious interpretation of the observed fluorescence pattern. Specifically, we now refer to the signal as localized enrichment at/near the septal region rather than a ring-like structure. We have also revised the corresponding descriptions in the Results section, Figure 7 legend, and Discussion to ensure that the interpretation is consistent with the limitations of the imaging approach. These changes help clarify that our data demonstrate a specific localization pattern of SmDOC1 at the septal region but do not by themselves establish the three-dimensional organization or precise molecular arrangement of the protein.

Line 510: define "SIN" and its function.

Response: We have now defined SIN at its first occurrence in the manuscript as the Septation Initiation Network (SIN) and added a brief description of its function. Specifically, we explain that SIN is a conserved fungal signaling pathway that coordinates cytokinesis and septum formation by regulating the timing and assembly of the division machinery. In filamentous fungi, SIN activity is particularly important for proper septation and cellular compartmentalization during growth and development.

Line 519: delete "the".

Response: was revised accordingly

Line 527 "While these data…"

Response: was revised accordingly

Line 570: during vegetative growth....

Response: was revised accordingly

Line 582-595: I was surprised that the localization of SmDOC1 to the septal pore (and specifically to the pore, not the entire septum), was not discussed in relation to its biological function in sexual development. This aspect needs some discussion, not just a list of localization of other proteins to septa in this paragraph.

Response: We thank the reviewer for this important suggestion. We agree that the specific localization of SmDOC1 to the septal pore, rather than the septum in general, warrants further discussion in the context of its potential biological function. We have expanded the Discussion to address that septal pores serve as regulated sites of intercompartmental communication in filamentous fungi and may contribute to cellular coordination during developmental processes, including sexual development. We now discuss that SmDOC1 localization at the septal pore may indicate a potential role in regulating communication or signaling events during development, while

emphasizing that this interpretation remains hypothetical and requires further experimental validation. We have added relevant references to support this discussion.

Reviewer #3

The authors have significantly improved the clarity and rigor of the manuscript and sufficiently addressed the concerns and comments raised by the reviewers.

Response: We sincerely thank the reviewer for the positive assessment of our revised manuscript. We appreciate the recognition that our revisions have improved the clarity, rigor, and overall presentation of the study. The constructive comments and suggestions provided during the review process have greatly helped us refine the manuscript, strengthen the interpretation of our results, and present our conclusions in a more balanced manner.
