# Supplementary material for: STRIPAK-associated greenbeard proteins DOC1 and DOC2 regulate MAK2 signaling and sexual development in Sordaria macrospora
Source: PLoS Biol. 2026 Aug 27;24(8):e3003969. doi: 10.1371/journal.pbio.3003969 (PMC13557503; doi:10.1371/journal.pbio.3003969)
Supplement: Supplementary file 4 [file pbio.3003969.s002.docx]

**S1 Table.** **Plasmids used in this study.**

| Plasmid | Characteristics | Reference |
| --- | --- | --- |
| p1783-1 | *amp^R^, ura3, hyg^R^*  *gpd::egfp:TtrpC* | [1] |
| p5’vac14-TagRFP-T | *amp^R^, ura3, nat^R^*  *5’vac14::vac14::TagRFP-T::TtrpC* | [2] |
| p5'-sci1-L-TurboID | *5’sci1*::sci1-L-TurboID::3xHA::TtrpC | [3] |
| p5'Smdoc2-L-TurboID | *amp^R^, ura3, nat^R^*  *5’Smdoc2::Smdoc2::L-TurboID::3xHA::TtrpC* | This study |
| pc-sci1-TurboID | *amp^R^, ura3, hyg^R^*  *ccg1::sci1-TurboID::3xHA::TtrpC* | [3] |
| pHRccg1Smdoc1-TagRFP-T | *amp^R^, ura3, nat^R^*  *5’Smdoc1::ccg1::Smdoc1::L-TagRFP-T::TtrpC::nat^R^::3’Smdoc1* | This study |
| pHRccg1Smdoc2-egfp | *amp^R^, ura3, nat^R^*  *5’Smdoc2::ccg1::Smdoc2::L-egfp::TtrpC::nat^R^::3’Smdoc2*  (GGGGS linker) | This study |
| pHRccg1Smdoc2-TagRFP-T | *amp^R^, ura3, nat^R^*  *5’Smdoc2::ccg1::Smdoc2::L-TagRFP-T::TtrpC::nat^R^::3’Smdoc2* | This study |
| pHRSmdoc1-TagRFP-T | *amp^R^, ura3, nat^R^*  *5’Smdoc1::Smdoc1::L-TagRFP-T::TtrpC::nat^R^::3’Smdoc1* | This study |
| pHRSmdoc1-TurboID | *amp^R^, ura3, nat^R^*  *5’Smdoc1::Smdoc1::L-TurboID::3xHA::TtrpC::nat^R^::3’Smdoc1* | This study |
| pHRSmdoc2-egfp | *amp^R^, ura3, nat^R^*  *5’Smdoc2::Smdoc2::L-egfp::TtrpC::nat^R^::3’Smdoc2*  (GGGS linker) | This study |
| pHRSmdoc2-TagRFP-T | *amp^R^, ura3, nat^R^*  *5’Smdoc2::Smdoc2::L-TagRFP-T::TtrpC::nat^R^::3’Smdoc2* | This study |
| pHRSmdoc2-TurboID | *amp^R^, ura3, nat^R^*  *5’Smdoc2::Smdoc2::L-TurboID::3xHA::TtrpC::nat^R^::3’Smdoc2* | This study |
| pRS_hyg | *amp^R^, ura3, hyg^R^* | [4] |
| pRS_nat | *amp^R^, ura3, nat^R^* | [5] |
| pRS426 | *amp^R^, ura3* | [6] |
| pSmdoc1+Smdoc2-KO | *amp^R^, ura3, hyg^R^*  *5’Smdoc1::hyg^R^::5’Smdoc2* | This study |
| pSmdoc1-KO | *amp^R^, ura3, hyg^R^*  *5’Smdoc1::hyg^R^::3’Smdoc1* | This study |
| pSmdoc2-KO | *amp^R^, ura3, hyg^R^*  *5’Smdoc2::hyg^R^::3’Smdoc2* | This study |
|  |  |  |
| **Yeast Two-Hybrid plasmids** |  |  |
| pAD-HAM5 | *HAM5* (*SMAC_02471*) cDNA in pGADT7 | [7] |
| pAD-MAK2 | *MAK2* (*SMAC_03492*) cDNA in pGADT7 | [7] |
| pAD-MEK2-t1 | *MEK2-t1* (*SMAC_06526-t1*) cDNA in pGADT7 | [7] |
| pAD-MEK2-t2 | *MEK2-t2* (*SMAC_06526-t2*) cDNA in pGADT7 | This study |
| pAD-ranBPM | *ranBPM* cDNA in pGADT7 | [8] |
| pAD-Smdoc1 | *Smdoc1* (*SMAC_06903*) cDNA in pGADT7 | This study |
| pAD-Smdoc2 | *Smdoc2* (*SMAC_06902*) cDNA in pGADT7 | This study |
| pBD-HAM5 | *HAM5* (*SMAC_02471*) cDNA in pGBKT7 | [7] |
| pBD-MAK2 | *MAK2* (*SMAC_03492*) cDNA in pGBKT7 | [7] |
| pBD-MEK2-t1 | *MEK2-t1* (*SMAC_06526-t1*) cDNA in pGBKT7 | [7] |
| pBD-MEK2-t2 | *MEK2-t2* (*SMAC_06526-t2*) cDNA in pGBKT7 | This study |
| pBD-Smdoc1 | *Smdoc1* (*SMAC_06903*) cDNA in pGBKT7 | This study |
| pBD-Smdoc2 | *Smdoc2* (*SMAC_06902*) cDNA in pGBKT7 | This study |
| pGADT7 | *LEU2, GAL4-AD, amp^R^, PADH1::GAL4AD::TADH1* | Takara Bio USA |
| pGBKT7 | *TRP1, GAL4-BD, kan^R^*  *PADH1::GAL4BD::TADH1* | Takara Bio USA |

*amp^R^,* ampicillin resistance; ura3, *orotidine-5’-phosphate decarboxylase* gene of *Saccharomyces cerevisiae*; *hyg^R^*, hygromycin resistance cassette expressing the *hygromycin B phosphotransferase* gene from *Escherichia coli* under control of the constitutive *trpC* promoter from *Aspergillus nidulans;* *nat^R^,* nourseothricin resistance cassette expressing the *nourseothricin acetyltransferase* gene from *Streptomyces noursei* under control of the constitutive *trpC* promoter from *A. nidulans*; *ccg1*, constitutive promoter of the *clock controlled gene 1* of *Neurospora crassa;* *gpd,* constitutive promoter of the *glyceraldehyde-3-phosphate dehydrogenase* gene of *A. nidulans;* *TtrpC,* terminator of the *anthranilate synthase* gene of *A. nidulans*; *egfp*, gene for the enhanced green fluorescent protein of *Aequorea victoria*; *TagRFP-T*, gene for the red fluorescent protein TagRFP-T of *Entacmaea quadricolor*; P, promoter; T, terminator; L, linker with the sequence GGGGSGGGGS if not indicated otherwise.

**References**

1.	Pöggeler S., Masloff S., Hoff B., Mayrhofer S., Kück U. Versatile EGFP reporter plasmids for cellular localization of recombinant gene products in filamentous fungi. Curr Genet. 2003;43(1):54–61. doi: 10.1007/s00294-003-0370-y.

2.	Groth A., Schmitt K., Valerius O., Herzog B., Pöggeler S. Analysis of the Putative Nucleoporin POM33 in the Filamentous Fungus *Sordaria macrospora*. J Fungi. 2021;7(9):682. doi: 10.3390/jof7090682.

3.	Hollstein L. S, Schmitt K., Valerius O., Stahlhut G., Pöggeler S. Establishment of *in vivo* proximity labeling with biotin using TurboID in the filamentous fungus *Sordaria macrospora*. Sci Rep. 2022;12(1):17727. doi: 10.1038/s41598-022-22545-x.

4.	Bloemendal S., Bernhards Y., Bartho K., Dettmann A., Voigt O., Teichert I., et al. A homologue of the human STRIPAK complex controls sexual development in fungi. Mol Microbiol. 2012;84(2):310–23. doi: 10.1111/j.1365-2958.2012.08024.x.

5.	Klix V., Nowrousian M., Ringelberg C., Loros J. J, Dunlap J. C, Pöggeler S. Functional characterization of MAT1-1-specific mating-type genes in the homothallic ascomycete *Sordaria macrospora* provides new insights into essential and nonessential sexual regulators. Eukaryot Cell. 2010;9(6):894–905. doi: 10.1128/EC.00019-10.

6.	Christianson T. W, Sikorski R. S, Dante M., Shero J. H, Hieter P. Multifunctional yeast high-copy-number shuttle vectors. Gene. 1992;110(1):119–22. doi: 10.1016/0378-1119(92)90454-w.

7.	Schmidt S., Märker R., Ramšak B., Beier-Rosberger A. M, Teichert I., Kück U. Crosstalk Between Pheromone Signaling and NADPH Oxidase Complexes Coordinates Fungal Developmental Processes. Front Microbiol. 2020;11:1722. doi: 10.3389/fmicb.2020.01722.

8.	Tucker C. L, Peteya L. A, Pittman A. MC, Zhong J. A genetic test for yeast two-hybrid bait competency using RanBPM. Genetics. 2009;182(4):1377–9. doi: 10.1534/genetics.109.103069.
