# Supplementary material for: STRIPAK-associated greenbeard proteins DOC1 and DOC2 regulate MAK2 signaling and sexual development in Sordaria macrospora
Source: PLoS Biol. 2026 Aug 27;24(8):e3003969. doi: 10.1371/journal.pbio.3003969 (PMC13557503; doi:10.1371/journal.pbio.3003969)
Supplement: Supplementary file 5 [file pbio.3003969.s003.docx]

**S2 Table. Primers used in this study.**

| Name | Sequence |
| --- | --- |
| h3 | gtactcgccgatagtggaaac |
| hph-f | gttaactgatattgaaggagcatttttgg |
| hph-f (RL76) | ccgcgacgtctgtcgagaag |
| hph-r | gttaactggttcccggtcggcatctactc |
| hph-r (RL77) | cattgtccgtcaggacattg |
| LH01-doc1-ko-5f | gtaacgccagggttttcccagtcacgacgacatcactccgttaaccacc |
| LH02-doc1-ko-5r | ccaaaaatgctccttcaatatcagttaactgtttcccgttccacaaagg |
| LH03-doc1-ko-3f | agagtagatgccgaccgggaaccagttaacaaactcatttcgagcgatt |
| LH04-doc1-ko-3r | gcggataacaatttcacacaggaaacagctcagacgcgctgcaattgca |
| LH05-doc2-ko-5f | gtaacgccagggttttcccagtcacgacgttcatacctcagatcggcac |
| LH06-doc2-ko-5r | ccaaaaatgctccttcaatatcagttaacgatggtggtaataaaacaat |
| LH07-doc2-ko-3f | agagtagatgccgaccgggaaccagttaatgacgatggaatgggaaagg |
| LH08-doc2-ko-3r | gcggataacaatttcacacaggaaacagcccgggcgacgggtgtcaatt |
| LH09-doc2-L-TurboID-r | ccgccgccgccgctgccgccgccgcccattgaaccaagctcaagagtca |
| LH101-d1HR-f | acatcactccgttaaccaccc |
| LH102-d1HR-r | tgcaattgcacaatgcctgatc |
| LH103-d2HR-f | tcatacctcagatcggcacatg |
| LH104-d2HR-r | acgggtgtcaattccttccag |
| LH109-d1-ccg1-f | aacacacaacctttgtggaacgggaaacagaattctagaaggagcagtccatctg |
| LH110-d1-ccg1-r | cgggagagcccttgccgctgctacccatgaattctttggttgatgtgaggggttgtg |
| LH119-AD-MEK2-t1f | tatggccatggaggccagtgaattcatggccgacccattcgcc |
| LH120-MEK2-t1-intr | tttggtacgctgaagtgggtgtaggttcatcgc |
| LH121-MEK2-t1-intf | acccacttcagcgtaccaaaccgacccc |
| LH122-AD-MEK2-t1r | tatcgatgcccacccgggtgctactcgcgctcccagtc |
| LH123-BD-MEK2-t1f | gcatatggccatggaggccgaattcatggccgacccattcgcc |
| LH124-BD-MEK2-t1r | gcaggtcgacggatccccggctactcgcgctcccagtc |
| LH125-AD-SmDOC1-f | tatggccatggaggccagtgaattcatgggtagcagcggcaag |
| LH126-AD-SmDOC1-r | tatcgatgcccacccgggtgtcaagcaataggcaaatccatacc |
| LH127-BD-SmDOC1-f | gcatatggccatggaggccgaattcatgggtagcagcggcaag |
| LH128-BD-SmDOC1-r | gcaggtcgacggatccccggtcaagcaataggcaaatccatacc |
| LH129-AD-SmDOC2-f | tatggccatggaggccagtgaattcatgatggcctccgcgacc |
| LH12-doc2-ko-5f2 | gagtagatgccgaccgggaaccagttaacgatggtggtaataaaacaat |
| LH130-AD-SmDOC2-r | tatcgatgcccacccgggtgtcatgaaccaagctcaagagtcaac |
| LH131-BD-SmDOC2-f | gcatatggccatggaggccgaattcatgatggcctccgcgacc |
| LH132-BD-SmDOC2-r | gcaggtcgacggatccccggtcatgaaccaagctcaagagtcaac |
| LH133-AD-HAM5-f | tatggccatggaggccagtgaattcatgtcggtccccggacac |
| LH134-AD-HAM5-r | tatcgatgcccacccgggtgtcagatcatctcactatgatgcaatcc |
| LH135-BD-HAM5-f | gcatatggccatggaggccgaattcatgtcggtccccggacac |
| LH136-BD-HAM5-r | gcaggtcgacggatccccggtcagatcatctcactatgatgcaatcc |
| LH137-MEK2-t2-int-r | gaatctcacctgaagtgggtgtaggttcatcgc |
| LH138-MEK2-t2-int-f | acccacttcaggtgagattcccattctg |
| LH139-AD-MEK2-t2-r | tatcgatgcccacccgggtgttaaaatcggccggttgg |
| LH13-doc2-ko-5r2 | cggataacaatttcacacaggaaacagctcatacctcagatcggcacat |
| LH140-BD-MEK2-t2-r | gcaggtcgacggatccccggttaaaatcggccggttgg |
| LH15-doc1-v5f | gcgatgtactccaagggtgg |
| LH16-doc1-v3r | cgatggatcttgccgttcct |
| LH18-doc1-vORF-3f | agcaggtcgtgtcaccattt |
| LH19-doc2-v5f | gcgacggcttcttcagtgat |
| LH20-doc2-v3r | gtgtacttgccacggacctt |
| LH21-doc2-vORF-3f | tgttgatggggagagtgtgc |
| LH24-so-doc1-3f | aagaaggcggggaaaacagg |
| LH25-so-doc2-5r2 | ctgcaccaagctgatggatg |
| LH26-so-doc2-5f2 | aggcgaaaagagtccacgg |
| LH27-doc1-vORF-f | tcagggtcatcgtcctccaa |
| LH28-doc1-vORF-r | tggtaggagcggaattgtgg |
| LH30-doc2-vORF-r | accaagctcaagagtcaacg |
| LH31-so-doc1-ORF-f | cggtgatacggacaggaacc |
| LH32-so-doc1-ORF-r | ataggtgccactctcgttgg |
| LH33-doc2-vORF-f2 | acgaagccaaggtcaaggag |
| LH36-HR-doc2TbID3f | cgctctacatgagcatgccctgcccctgatgacgatggaatgggaaagg |
| LH37-HR-T-pTrpC-r | agcccaaaaaatgctccttcaatatcagttcgagtggagatgtggagtg |
| LH38-HR-doc2-nat-f | actgatattgaaggagcattttttgggcttg |
| LH41-doc2-HR-TurboID | acagtccaaacaactccgct |
| LH85-doc1-5r-BglII-2 | tgcttaagatcttgtttcccgttccacaaaggt |
| LH86-ccg1-f-EcoRI-2 | taagcagaattctagaaggagcagtccatctgc |
| LH88-HR5'doc1-f | cgacggtatcgataagcttgatatcgcggccgcacatcactccgttaaccaccc |
| LH89-HR5'doc1-r | acccatgaattctgtttcccgttccacaaaggttg |
| LH90-HRdoc1-ORF-f | ggaacgggaaacagaattcatgggtagcagcggcaagggc |
| LH91-HRdoc1-ORF-r | gcttcctcctcctccggatcctccgccgccagcaataggcaaatccatacccccc |
| LH92-HRd1-tRFP-1 | ggcggcggaggatccggaggaggaggaagcgtgtctaagggcgaagagctg |
| LH93-HRTtrpC-nat | ttcaatatcagttcgagtggagatgtggagtgg |
| LH94-HRnat-TtrpC | catctccactcgaactgatattgaaggagcattttttgggcttg |
| LH95-HRd1-nat-3' | cgaaatgagtttgtcaggggcagggcatgctcatg |
| LH96-HRd13'-f | ccctgcccctgacaaactcatttcgagcgattcatgaac |
| LH97-HRd13'-r | caaaagctggagctccaccgcggtgtcagacgcgctgcaattgcac |
| LH98-HRd1-Tb | ggcggcggaggatccggaggaggaggaagcaaggacaacaccgtccccctc |
| MAK2KO1 | ctcctgtttattcctccatcagct |
| MAK2KO2 | cctcctcgagagcgaacacatcat |
| nat-1r | tcaggggcagggcatgctca |
| pro11-21 | aagcgcgcttgccagtcgctgc |
| Pro115r_wohph | agttgtgcggttgtcgttggtcgaagc |
| pro11-KOr | acgatcagcctcggaaagaccgc |
| pRS_seq_F | ggcctcttcgctattacgccag |
| pRS_seq_R | cactcattaggcaccccagg |
| sci_f | tcgcaagtcatgtacaccat |
| sci1_vf | aggtcagctcccaacagcaagt |
| sci1_vr | ttgggatcggttcgtggaga |
| Seq_tBioID_rev | gtctggatgtgcttgttgatg |
| SmtBioID-L-f-2 | atgggcggcggcggcagcggcggc |
| sosci1_f | ctggtaggcacctagcccac |
| sosci1_r | gccgtggatgaatggagatc |
| tC1 | caccgcctggacgactaaacc |
| tRFP-f | acgtcgagcagcacgaggtg |
| TtrpC_pRS_r | gcggataacaatttcacacaggaaacagctcgagtggagatgtggagtgg |
