# Supplementary material for: STRIPAK-associated greenbeard proteins DOC1 and DOC2 regulate MAK2 signaling and sexual development in Sordaria macrospora
Source: PLoS Biol. 2026 Aug 27;24(8):e3003969. doi: 10.1371/journal.pbio.3003969 (PMC13557503; doi:10.1371/journal.pbio.3003969)
Supplement: Supplementary file 6 [file pbio.3003969.s004.docx]

**S3 Table. Strains used in this study.**

| Strain | Genotype | Reference |
| --- | --- | --- |
| ***Sordaria macrospora*** |  |  |
| ∆pro11 | *Δpro11:hyg^R^*, ssi, sterile | [1] |
| ∆pro11∆sci1::sci1-TurboID^ect^ | cross of Δsci1::sci1-TurboID^ect^ with ∆pro11, ssi, sterile, *hyg^R^, nat^R^* | This study |
| ∆sci1 | *Δsci1::hyg^R^*, ssi, sterile | [2] |
| ∆sci1::sci1-TurboID^ect^ | ectopic integration of *sci1-TurboID* (native *sci1*-promoter) into Δsci1, ssi, fertile, *hyg^R^, nat^R^* | [3] |
| fus1-1 | mutation in the *Trihydroxynaphthalene reductase* gene (*SMAC_05650*), light brown ascospores, ssi, fertile | [4] |
| Smdoc1::Smdoc1-TagRFP-T | integration of *Smdoc1-TagRFP-T* at the native *Smdoc1* locus, ssi, fertile, *Smdoc1-TagRFP-T::nat^R^* | This study |
| Smdoc1::Smdoc1-TurboID | integration of *Smdoc1-TurboID* at the native *Smdoc1* locus, ssi, fertile, *Smdoc1-TurboID::nat^R^* | This study |
| Smdoc2::Smdoc2-egfp | integration of *Smdoc2-egfp* at the native *Smdoc2* locus, ssi, fertile, *Smdoc2-egfp::nat^R^* | This study |
| Smdoc2::Smdoc2-TurboID | integration of *Smdoc2-TurboID* at the native *Smdoc2* locus, ssi, fertile, *Smdoc2-TurboID::nat^R^* | This study |
| wild type (wt) | wild type strain, black ascospores, fertile | DSM997, DSMZ |
| wt::egfp-TurboID^ect^ | ectopic integration of *egfp-TurboID* into the *S. macrospora* wild type, ssi, fertile, *nat^R^* | This study |
| wt:free-TurboID^ect^ | ectopic integration of free unfused *TurboID* (*ccg1* promoter) into the *S. macrospora* wild type, *hyg^R^* | [3] |
| Δku70 | *Δku70::nat^R^*, ssi, fertile | [5] |
| Δku80 | *Δku80::hyg^R^*, ssi, fertile | [6] |
| Δku80::pccg1-Smdoc1-TagRFP-T^ect^ | ectopic integration of *ccg1::Smdoc1-TagRFP-T::nat^R^* into Δku80, ssi, fertile, *hyg^R^, nat^R^* | This study |
| Δku80::pccg1-Smdoc2-egfp^ect^ | ectopic integration of *ccg1::Smdoc2-egf*p::natR into Δku80, ssi, fertile, *hyg^R^, nat^R^* | This study |
| Δku80::pccg1-Smdoc2-TagRFP-T^ect^ | ectopic integration of *ccg1:Smdoc2-TagRFP-T::natR* into Δku80, ssi, fertile, *hyg^R^, nat^R^* | This study |
| Δku80::Smdoc2-TagRFP-T^ect^ | ectopic integration of *Smdoc2-TagRFP-T::nat^R^* (native 1 kb 5' region) into Δku80, ssi, fertile, *hyg^R^, nat^R^* | This study |
| Δmak2 | marker-less deletion of *mak2*, Δmak2, fus 1-1, brown ascospores, ssi, sterile | [7] |
| Δmak2;Smdoc2::Smdoc2-TurboID | cross of Δmak2 with Smdoc2::Smdoc2-TurboID, expression of *Smdoc2-TurboID* at the native *Smdoc2* locus in the Δmak2 deletion strain background, ssi, sterile, *Smdoc2-TurboID::nat^R^* | This study |
| ΔSmdoc1 | deletion of *SMAC_06903*, *ΔSmdoc1::hyg^R^*, ssi, impaired sexual development | This study |
| ΔSmdoc1::Smdoc1^ect^ | ectopic integration of the *Smdoc1* ORF with 1 kb 5' and 3' flanks, fertile, *hyg^R^, nat^R^* | This study |
| ΔSmdoc1ΔSmdoc2 | deletion of the genomic region expressing *SMAC_0690*3 and *SMAC_06902*, *ΔSmdoc1ΔSmdoc2::hygR*, ssi, fertile | This study |
| ΔSmdoc2 | deletion of *SMAC_06902*, *ΔSmdoc2::hyg^R^*, ssi, impaired sexual development | This study |
| ΔSmdoc2::Smdoc2^ect^ | ectopic integration of the *Smdoc2* ORF with 1 kb 5' and 3' flanks, fertile, *hyg^R^, nat^R^* | This study |
|  |  |  |
| ***Neurospora crassa*** |  |  |
| GN18-16 | *mat a,* Δ*doc-1::hyg^R^* | [8] and this study |
| GN18-17 | *mat A,* Δ*doc-1::hyg^R^* | [8] and this study |
| GN18-19 | *mat A,* Δ*doc-1*Δ*doc-2::hyg^R^* | [8] and this study |
| GN18-26 | *mat a,* Δ*doc-1*Δ*doc-2::hyg^R^* | [8] and this study |
| GN18-34 | *mat a,* Δ*doc-2::hyg^R^* | [8] and this study |
| GN18-35 | *mat A,* Δ*doc-2::hyg^R^* | [8] and this study |
| GN18-36 | *mat a,* Δ*doc-2::hyg^R^* | [8] and this study |
| FGSC 14643 | *mat a,* Δ*doc-2::hyg^R^* | FGSC |
| FGSC 14644 | *mat A,* Δ*doc-2::hyg^R^* | FGSC |
|  |  |  |
| ***Saccharomyces cerevisiae*** |  |  |
| PJ69-4A | *MATa, trp1-901 leu2-3_112 ura3-52 his3_200 gal4Δ gal8O Δ LYS2::GAL1-HIS3 GAL2-ADE2 met2::GAL7-lacZ* | [9] |
| Y187 | *MATα, ura3-52, his3-200,ade2-101, trp1-901, leu2-3, 112, gal4Δ, gal80Δ, met–, URA3::GAL1_UAS_–Gal1_TATA_–LacZ, MEL1* | [10] |
| Y2H Gold | *MATa, trp1-901, leu2-3, 112, ura3-52, his3-200, gal4Δ, gal80Δ, LYS2::GAL1_UAS_–Gal1_TATA_–His3, GAL2_UAS_–Gal2_TATA_–Ade2, URA3::MEL1_UAS_–Mel1_TATA_, AUR1-C MEL1* | Nguyen, unpublished obtained from Takara Bio USA (Matchmaker® Gold Yeast Two-Hybrid System) |
|  |  |  |
| ***Escherichia coli*** |  |  |
| MACH1^TM^ | F- φ80(lacZ)∆M15 ∆lacX74 hsdR(r_K_^-^m_K_^+^) ∆recA1398 endA1 tonA | Invitrogen |

ect, ectopic integration; ssi, single spore isolate; *hyg^R^*, hygromycin resistance cassette expressing the *hygromycin B phosphotransferase* gene from *E. coli* under control of the constitutive *trpC* promoter from *A. nidulans;* *nat^R^,* nourseothricin resistance cassette expressing the *nourseothricin acetyltransferase* gene from *S. noursei* under control of the constitutive *trpC* promoter from *A. nidulans;* *ccg1,* promoter of the *clock controlled gene 1* of *N. crassa*; *egfp*, gene for green fluorescent protein enhanced green fluorescent protein of *Aequorea victoria*; *TagRFP-T,* gene for red fluorescent protein TagRFP-T of *Entacmaea quadricolor;* ORF, open reading frame; DSMZ, Deutsche Sammlung von Mikroorganismen und Zellkulturen (Leibniz Institut, Braunschweig).

**References**

1.	Bloemendal S., Bernhards Y., Bartho K., Dettmann A., Voigt O., Teichert I., et al. A homologue of the human STRIPAK complex controls sexual development in fungi. Mol Microbiol. 2012;84(2):310–23. doi: 10.1111/j.1365-2958.2012.08024.x.

2.	Reschka E. J, Nordzieke S., Valerius O., Braus G. H, Pöggeler S. A novel STRIPAK complex component mediates hyphal fusion and fruiting-body development in filamentous fungi. Mol Microbiol. 2018;110(4):513–32. doi: 10.1111/mmi.14106.

3.	Hollstein L. S, Schmitt K., Valerius O., Stahlhut G., Pöggeler S. Establishment of *in vivo* proximity labeling with biotin using TurboID in the filamentous fungus *Sordaria macrospora*. Sci Rep. 2022;12(1):17727. doi: 10.1038/s41598-022-22545-x.

4.	Nowrousian M., Teichert I., Masloff S., Kück U. Whole-Genome Sequencing of *Sordaria macrospora* Mutants Identifies Developmental Genes. G3-Genes Genomes Genetics. 2012;2(2):261–70. doi: 10.1534/g3.111.001479.

5.	Pöggeler S., Kück U. Highly efficient generation of signal transduction knockout mutants using a fungal strain deficient in the mammalian *ku70* ortholog. Gene. 2006;378:1–10. doi: 10.1016/j.gene.2006.03.020.

6.	Groth A., Schmitt K., Valerius O., Herzog B., Pöggeler S. Analysis of the Putative Nucleoporin POM33 in the Filamentous Fungus *Sordaria macrospora*. J Fungi. 2021;7(9):682. doi: 10.3390/jof7090682.

7.	Schmidt S., Märker R., Ramšak B., Beier-Rosberger A. M, Teichert I., Kück U. Crosstalk Between Pheromone Signaling and NADPH Oxidase Complexes Coordinates Fungal Developmental Processes. Front Microbiol. 2020;11:1722. doi: 10.3389/fmicb.2020.01722.

8.	Heller J., Zhao J., Rosenfield G., Kowbel D. J, Gladieux P., Glass N. L. Characterization of Greenbeard Genes Involved in Long-Distance Kind Discrimination in a Microbial Eukaryote. PLoS Biol. 2016;14(4):e1002431. doi: 10.1371/journal.pbio.1002431.

9.	James P., Halladay J., Craig E. A. Genomic libraries and a host strain designed for highly efficient two-hybrid selection in yeast. Genetics. 1996;144(4):1425–36. doi: 10.1093/genetics/144.4.1425.

10.	Harper J. W, Adami G. R, Wei N., Keyomarsi K., Elledge S. J. The p21 Cdk-interacting protein Cip1 is a potent inhibitor of G1 cyclin-dependent kinases. Cell. 1993;75(4):805–16. doi: 10.1016/0092-8674(93)90499-g.
