# Supplementary material for: STRIPAK-associated greenbeard proteins DOC1 and DOC2 regulate MAK2 signaling and sexual development in Sordaria macrospora
Source: PLoS Biol. 2026 Aug 27;24(8):e3003969. doi: 10.1371/journal.pbio.3003969 (PMC13557503; doi:10.1371/journal.pbio.3003969)
Supplement: Supplementary file 8 [file pbio.3003969.s048.docx]

# Reviewer #1:

The manuscript presents a novel conceptual advance by identifying DOC proteins as shared regulatory components linking the STRIPAK phosphatase complex and the pheromone response (PR) MAPK signaling pathway. While DOC proteins were previously characterized in *Neurospora crassa*, their integration into STRIPAK-dependent signaling networks and their functional role in sexual development, particularly in a homothallic species, has not been previously reported. The integration of genetic, proteomic, and interaction-based data provides a framework for understanding how signaling specificity and pathway crosstalk are regulated in fungi, with implications for conserved eukaryotic signaling mechanisms. The experimental approaches employed (genetic analysis, BioID-based proximity labeling, mass spectrometry, and yeast two-hybrid assays) are appropriate and generally well executed. Proteomic analyses are well controlled, statistically robust, and supported by extensive supplementary data. However, several conclusions rely heavily on proximity labeling and descriptive phenotypes without sufficient functional or quantitative validation. In particular, the interpretations related to subcellular localization, direct protein-protein interactions (especially for SmDOC2), and the proposed mechanistic model of PR complex disassembly exceed what is directly supported by the data. No major new experimental approaches are strictly required for publication, but the paper would be substantially improved by: more cautious interpretation of BioID and localization data, clearer labeling of the speculative proposed models, improved figure and text consistency, and where feasible, quantification of key phenotypes.

Response: We thank the reviewer for the thoughtful and constructive evaluation of our manuscript and for recognizing the conceptual advance of identifying DOC proteins as shared regulatory components linking the STRIPAK phosphatase complex with the pheromone response (PR) MAPK pathway. We also appreciate the positive assessment of the experimental design, the robustness of the proteomic analyses, and the integration of genetic, proteomic, and interaction-based approaches. We agree with the reviewer that some conclusions—particularly those related to subcellular localization, direct protein–protein interactions (notably for SmDOC2), and the proposed mechanistic model of PR complex disassembly—relied too heavily on proximity labeling and descriptive phenotypes. In response, we have revised the manuscript to ensure that interpretations are more conservative and strictly aligned with the data presented.

1. In the introduction, the technical descriptions of the Turbo ID experiment are thorough and appreciated but may be shortened in the introduction, or fit in with the first results section or the methods. Given that the TurboID workflow has been previously established in this system and validated by the authors in their previous paper, it might be worth considering whether the schematic in Figure 1 could be streamlined or integrated with a subsequent figure, to focus directly on the biological results.

Response: The BioID workflow figure was moved into the supplements and the technical description of the experimental setup was moved to the results section.

2. In figure 2B, the authors may wish to briefly note how the volcano plot presented relates to the volcano plot shown in the previous paper (Hollstein et al 2022), or whether the data were newly generated or reanalyzed using different statistical criteria.

Response: The volcano plot shown in Figure 2B (now Fig. 1B) is based on newly generated BioID proteomic data and is not a reanalysis of the dataset presented in Hollstein et al. (2022). To avoid confusion and improve clarity, we have now explicitly stated this in the corresponding Results section. We also briefly note that, although the overall analytical workflow is similar to that used previously, the data shown here derive from independent experiments and were processed using the statistical criteria described in the Methods section of the present study.

The volcano plot shown in Figure 2B (now Fig. 1B) is based on the LC-MS raw files SP106-113 and LH35-LH42 and have not been shown in any previous study. The previous paper Hollstein et al. (2022) shows the raw files SP87-94 and the *S. macrospora* strains were cultured on different media. The experiment presented in the current manuscript was performed on the complex Biomalt maize medium and includes more replicates for a more robust statistical analysis.

3. For transparency, the legend of Fig S9 should indicate that the wild-type, Smdoc1, and Smdoc2 images shown are the same images used in Fig 3B.

Response: We thank the reviewer for highlighting this issue. For transparency, we have now revised the legend of Figure S9 (now S11 Fig) to explicitly state that the wild-type, *Smdoc1*, and *Smdoc2* images shown are the same images used in Figure 3B (now Fig. 2B). This clarification has been added to ensure consistency and avoid any potential confusion for the reader.

4. In Fig 3 and Fig S9, the Smdoc1::DOC1 and Smdoc2::DOC2 complemented strains appear to exhibit lighter lid coloration than the wild-type. Is there a delay or defect in mature fruiting body formation? It is also worth noting that the double knockout mutant overall appears more similar to wild-type than either complemented strain, which perhaps could be due to ectopic integration of the complementation constructs. Many of the phenotypes in this section are presented descriptively. Incorporation of quantitative analyses for at least some assays would strengthen the robustness of the conclusions.

Response: We thank the reviewer for this careful assessment of the phenotypes shown in Fig. 3 and Fig. S9. There is an apparently lighter lid coloration observed in the *Smdoc1::DOC1* and *Smdoc2::DOC2* complemented strains. We agree that the overall appearance of the double knockout mutant is, in some respects, closer to wild-type than either complemented strain. As suggested by the reviewer, this may result from ectopic integration and/or altered expression levels of the complementation constructs. We have now explicitly discussed this possibility in the manuscript and have taken care to avoid interpreting these differences as biologically meaningful phenotypes intrinsic to DOC function. In response to the reviewer’s broader concern regarding descriptive phenotypes, we have incorporated picture of ascus rosettes (new S12 Fig.), a quantification of ejected spores (new S13 Fig.) and we have updated the corresponding figure panels and legends. Where quantification was not practical, we have revised the text to clearly state that the conclusions are based on qualitative observations.

5. Several of the results in the text regarding the sexual development of N. crassa are not directly illustrated in Fig 4, which appears to only show self crosses. It would be helpful to clarify whether these reciprocal crosses and ascospore data are shown elsewhere, or if these are intended to function as representative images.

Response: See response #5 to Reviewer 2. Since none of the mutants showed a defect in sexual development, the additional heterozygous crosses became dispensable.

6. The results sections on MAK2 interactions and MEK2 analyses are supported primarily by supplemental tables and figures. A main text figure would help orient the reader to appreciate the relevance of DOC and MAP kinase signaling.

Response: To better orient the reader and highlight the relevance of DOC proteins in MAP kinase signaling, we have integrated the hypothetical model (Fig. 8) as a main text figure. This figure integrates the most relevant data previously presented in the supplemental tables and figures and provides a conceptual overview of DOC-associated MAP kinase signaling.

7. For the yeast two hybrid assay, given the parallel analysis of SmDOC1 and SmDOC2 in earlier sections, a brief explanation of whether SmDOC2 was also tested in the Y2H system is warranted. And if so, with what outcome.

Response: We thank the reviewer for this important point. SmDOC2 was indeed tested in the yeast two-hybrid (Y2H) assays in parallel with SmDOC1. However, no reproducible interactions were detected for SmDOC2 under the conditions tested. We have now added a brief statement in the Results section to clarify that SmDOC2 was analyzed in the Y2H system and to report the negative outcome. We also added the positive control mating of AD-ranBPM with either BD-SmDOC1 or BD-SmDOC2 to the main figure to show the reduced cell viability of the SmDOC2 expressing cells.

8. Regarding the interpretation of the fluorescence localization data for SmDOC1/2 in Fig. 8: the SmDOC1-TagRFP signal is extremely weak and difficult to visually discern. Moreover, comparison with the control images in the Supplemental Figures is confusing: the panel labeled "wild type'" in S19 Fig appears to show a tagged strain, and untagged wild-type controls are not clearly presented for SmDOC1. For SmDOC2, the untagged wild type shows septal fluorescence patterns in both green and red channels that appear indistinguishable from those observed in tagged strains (S21 Fig), consistent with autofluorescence. As currently presented, the reader cannot assess whether the observed septal signal reflects specific DOC localization or background fluorescence.

Response: We agree that the SmDOC1-TagRFP signal is weak and that the original presentation did not provide sufficient untagged controls to distinguish specific localization from background. Similarly, for SmDOC2, the apparent septal fluorescence observed in untagged wild-type controls (S22 and S23 Fig.) indicates significant autofluorescence, making it difficult to definitively assign the septal signal to the tagged protein. We now explicitly state in the Results and Discussion sections that the SmDOC1-TagRFP signal is weak. We have updated Figure S22 and S23 legends to clearly distinguish tagged strains from untagged wild-type controls. Where possible, we have added representative images of untagged wild-type cells alongside tagged strains to aid interpretation. Any discussion in the main text regarding specific septal localization of SmDOC1 or SmDOC2 has been revised to reflect that these observations are suggestive but not conclusive, and that autofluorescence may contribute to the signal.

9. In the Discussion, the genetic analyses support an important role for DOC proteins in sexual development, however, more explicitly qualifying species-specific conclusions (defects demonstrated in N. crassa) would better reflect the largely descriptive nature of the phenotypic data and avoid broader generalization to heterothallic fungi.

Response: We have revised the Discussion to explicitly qualify our conclusions as species-specific and to avoid generalization.

10. The section on SmDOC1 localization appears to overstate the strength of the fluorescence microscopy data. While Fig 8 is interpreted as showing ring-like localization at the septal pore, the signal is extremely weak and, as shown in the Supplemental Figures, similar septal fluorescence is observed in untagged wild-type controls, suggesting autofluorescence. Additionally, enrichment of septal-associated proteins in BioID reflects proximity rather than definitive spatial localization. Softening the language in this section and more explicitly acknowledging these limitations would better align with the presented data.

Response: We agree that the current text slightly overstates the strength of the fluorescence microscopy data for SmDOC1. As noted, the SmDOC1-TagRFP signal is weak, and similar septal fluorescence in untagged wild-type controls indicates that some of the observed signal might reflects autofluorescence. We also acknowledge that BioID enrichment indicates spatial proximity rather than definitive septal localization. We soften the language regarding septal localization – descriptions of “ring-like” or “septal” localization now explicitly note that these observations are suggestive and not conclusive. We clarify that weak fluorescence signals and background autofluorescence prevent definitive assignment of SmDOC1 to the septum and now emphasize that BioID indicates proximity to septal-associated proteins but does not confirm precise subcellular localization.

11. The statements describing SmDOC1/2 as "closely interacting" with PR signaling components appear stronger than what is directly supported by the data. Given that BioID reports proximity rather than physical interaction, and Y2H interactions are demonstrated only for SmDOC1 and not SmDOC2.

Response: We agree that the original wording implying “close interaction” overstates the conclusions that can be drawn from our data. While BioID identifies proteins in spatial proximity, it does not confirm direct physical interactions, and Y2H interactions were detected only for SmDOC1, not SmDOC2.

12. Fig 10 presents an interesting and coherent mechanistic model, however, the stepwise/directional nature of the diagram implies experimental validation that is not directly demonstrated in this study. In particular, reciprocal inhibition between SmDOC1 and SmDOC2, their proposed activation state, and their role in PR complex disassembly are inferred. I would suggest more explicitly labeling this model as speculative and adjusting both the figure and accompanying text to distinguish hypothesis from data-supported conclusions. Alternatively, the model could be framed as one of several possible mechanisms explaining the observed non-additive phenotypes, with reciprocal antagonism and PR complex disassembly presented as testable hypotheses.

Response: We agree that the stepwise and directional presentation in Figure 10 could be interpreted as implying experimental validation that is not directly provided in this study. In particular, the reciprocal inhibition between SmDOC1 and SmDOC2, their proposed activation states, and their role in PR complex disassembly are inferred rather than directly demonstrated. We explicitly label the model as speculative, and its legend now clearly indicate that the proposed mechanisms are hypotheses consistent with the observed phenotypes.

We clarify that directional arrows and mechanistic annotations are “proposed” or “putative” interactions and we have added text noting that the model represents one possible explanation for the non-additive phenotypes observed, and that reciprocal antagonism and PR complex disassembly are testable hypotheses for future studies. We altered the wording of the paragraph to emphasize that we present a hypothetical and speculative model, which now requires precise testing and verification of the various interactions and the temporal scale.

# Reviewer #2:

Review of "Capture of the SmSTRIPAK proxiome identifies the greenbeard proteins SmDOC1/2 as regulators of the MAK2 pathway to control sexual development in Sordaria macrospora" by Hollstein et al., describes the identification of new proteins using biotin-labeling (Turbo-ID) that were associated with the STRIPAK complex in the filamentous fungus Sordaria macrospora. This reviewer appreciated the carefulness in data and controls for the Turbo-ID experiments. The new proteins identified had previously been characterized in the Neurospora crassa (Doc1 and Doc2) and regulate chemotropic interactions associated with cell fusion. The authors characterized deletion mutants of SmDoc1 and SmDoc2 and showed that they displayed sexual reproduction defects. The authors then used Turbo-ID to identify proteins that associated with SmDoc1 and SmDoc2 and a component of a pheromone response MAP kinase pathway. These results indicate an interaction between the STRIPAK complex and the pheromone response pathway via the Doc proteins to regulate sexual development in S. macrospora. This investigation is well written, with some suggested comments below. Major considerations are also listed below.

Response: We thank the reviewer for the positive and thoughtful evaluation of our work. We appreciate the recognition of the careful experimental design and controls used in the TurboID experiments, as well as the acknowledgment of the relevance of Doc1 and Doc2 in chemotropic interactions and sexual development. We are pleased that the reviewer found the identification of interactions between the STRIPAK complex and the pheromone response MAP kinase pathway to be well supported and clearly presented.

We have carefully considered all suggested comments and major considerations and have revised the manuscript accordingly. These revisions have improved the clarity of the presentation and strengthened the interpretation of our results. Detailed responses to each specific comment are provided below.

Major considerations

1. In the introduction, the regulation of the doc system in N. crassa of the oscillation of the components of the MAP kinase pathway during chemotrophic interactions needs to be described. The mention of this aspect in the discussion is not interpretable without this information. For example, in the discussion is the first time oscillation is mentioned "Multi-phosphorylated SmDOC1/2 might bind PR components more tightly without releasing them. This would result in a situation of competitive inhibition, which prevents the assembly of the MAPK signaling cascade and thereby suppresses oscillation".

Response: We agree that the regulation of the DOC system and the oscillatory behavior of MAP kinase pathway components during chemotropic interactions in *Neurospora crassa* should be introduced earlier to provide the necessary context for the discussion. To address this, we have revised the Introduction to include a description of the oscillatory dynamics of MAP kinase signaling during chemotropic interactions in *N. crassa* and the role of the DOC system in this process. This additional background now allows the reader to better understand the reference to oscillation in the Discussion, including our proposed model in which altered phosphorylation of SmDOC1/2 may affect the assembly and dynamics of the MAPK signaling cascade.

2. Some of the introduction could be tightened up (some methods of the Turbo-ID could be moved to results).

Response: We agree that parts of the Introduction contained methodological details that are better suited for later sections. Accordingly, we have tightened the Introduction by removing or shortening descriptions of the TurboID methodology and relocating these details to the Results section, where they are directly relevant to the experimental findings.

3. Cell fusion is known to be important for the formation of reproductive structures in both S. macrospora and N. crassa; the DOC proteins regulate this process in N. crassa. I was surprised that this aspect of the SmDoc1 ad Smdoc2 mutants was not addressed in S. macrospora, as this could be the cause of the inability to form reproductive structures. In particular, the cell fusion mutants in N. crassa also show a delay in colony establishment.

Response: We agree that cell fusion plays a critical role in the formation of reproductive structures in both *S. macrospora* and *N. crassa*, and that the DOC proteins are key regulators of this process in *N. crassa*. We also recognize that defects in cell fusion could potentially contribute to the impaired sexual development observed in the *Smdoc1* and *Smdoc2* deletion mutants. Therefore, we have included photos from an analysis showing that the vegetative hyphae of *Smdoc1* and *Smdoc2* mutants are not impaired in the formation of fusions (new Fig. S9).

To address this point, we have expanded the Discussion to explicitly consider impaired cell fusion as a possible underlying cause of the observed sexual reproduction defects in *S. macrospora*. We now discuss the known roles of DOC proteins in regulating chemotropic interactions and cell fusion in *N. crassa*, and relate these findings to the phenotypes observed in the *Smdoc1* and *Smdoc2* mutants. In addition, we note that, in contrast to *N. crassa* cell fusion mutants, the *S. macrospora* deletion strains did not show a pronounced delay in colony establishment under our experimental conditions, suggesting that the defects in sexual development may not be solely attributable to general growth or early colony formation defects. Hyphal fusion events in the vegetative mycelium were observed for both single knockout strains (S9 Fig).

4. An explanation of why the sci1 subunit of the SmSTRIPAK complex was used as bait is needed.

Response: We thank the reviewer for this comment. We have now clarified in the manuscript why the STRIPAK subunit SCI1 was chosen as bait for the TurboID experiments. Specifically, SCI1 represents a core component of the SmSTRIPAK complex and has previously been shown to interact with multiple STRIPAK-associated proteins and signaling components. Its central position within the complex makes it a suitable and informative bait to identify proteins associated with SmSTRIPAK and to capture transient or proximal interactions relevant for signal integration. This rationale has been added to the Results section, thereby providing clearer justification for the experimental design.

5. Are the doc-1 and doc-2 mutants from the N. crassa deletion collection or from the strains constructed by Heller et al.,? It is known that secondary mutations are common in the deletion strain collection, and these mutations are often in cell fusion genes; cell fusion mutants in N. crassa are defective/very delayed for protoperithecia formation. If from the N. crassa deletion collection, the phenotype of the doc-1/doc-2 mutants would need to be confirmed to be dependent on these mutations and not background mutations.

Response: The *doc-1* and *doc-1/doc-2* double mutants were based on strains constructed by Heller et al. The *doc-2* mutant originated from the *Neurospora* gene knockout collection. This strain had previously been verified by PCR for correct integration of the knockout cassette and absence of the wild-type allele. Since the strain did not display the macroscopic phenotype typically associated with the known secondary mutation (with which we are very familiar), we initially did not perform a segregation analysis.

In response to the reviewer’s comment, we revisited this issue and performed a segregation analysis. All progeny exhibited a wild-type-like macroscopic phenotype, indicating that the well-characterized secondary mutation affecting cell fusion and sexual development was not present in the original strain. However, when we assessed protoperithecia formation in individual progeny, approximately half of the strains formed protoperithecia, whereas the other half did not. This pattern strongly suggests the presence of an additional, distinct secondary mutation in the original strain.

To address this issue, we reconstructed a *doc-2* mutant by crossing an auxotrophic *doc-2* strain (generated by Heller et al.) with the wild type. From this first cross, all analyzed *doc-2* progeny displayed wild-type-like protoperithecia formation. One of the prototrophic progeny strains was subsequently used to repeat the experiments described in the manuscript, confirming that the *doc-2* mutant does not exhibit defects in sexual development.

We very much thank the reviewer for raising this important issue, which allowed us to clarify the genetic background of the strain and avoid drawing an incorrect conclusion!

6. Fig. 1 (Turbo-ID method) could go into supplemental data.

Response: We agree that Fig. 1 primarily illustrates methodological aspects of the TurboID approach. Accordingly, we have moved Fig. 1 to the Supplemental Data section and adjusted the figure numbering and text references throughout the manuscript. This change streamlines the main figures and allows the Results section to focus more directly on the biological findings.

7. There is an exceptionally large number of supplemental figures and tables. I suggest perhaps combining some of the excel spreadsheets on the Turbo-ID data with separate sheets in the same excel file and some of the figures could be combined (S5-S7, S10-S11, for example). The supplemental tables are not listed numerically in the manuscript (the first supplemental table mentioned is S6).

Response: We thank the reviewer for this helpful organizational suggestion. We agree that the number of supplemental figures and tables was extensive and could be streamlined to improve clarity and accessibility. In response, we have reorganized the Supplemental Data by combining related TurboID Excel spreadsheets into single files with multiple worksheets (S1-S10 Data). As a result, the total number of supplemental items has been reduced.

We have also carefully revised the manuscript to ensure that all supplemental tables and figures are now cited in numerical order, beginning with Table S1, and that all references in the text correspond correctly to the revised supplemental numbering.

We believe these changes substantially improve the organization and usability of the Supplemental Data.

8. There are some grey dots that showed high significance in the volcano plots, but they are not labeled. Either indicate what these proteins are in the plot or in the legend.

Response: We agree that the unlabeled grey dots representing highly significant proteins in the volcano plots were unclear. However, adding a label to every grey dot would crowd the figure too much and render it unreadable. Additionally, some of these proteins appear to be enriched due changes in their global protein abundance, rather than their proximity to the bait-TurboID fusion. All significant proteins are listed in the supplementary data (S1-S10 Data) with details on their spectral counts, unique peptide counts, sequence coverage, modified peptides and many more metrics. Furthermore, the processed raw files will be publicly available upon publication at the PRIDE archive (currently only accessible with reviewer log in details).

9. Protoperithecia in the *N. crassa* doc-2 mutant not present in Fig. 4—perhaps a later photo showing formation of these structures?

Response: See response #5 to reviewer 2.

10. Figures 5 and 6 could be combined.

Response: Accordingly, we have merged the two figures into a single composite figure, with clear labeling of the panels to distinguish the different datasets. The figure legend has been updated to describe all panels, and the text references have been adjusted accordingly.

11. Why was autofluorescence in the red and green channel to the septa identified in Figure S14 and S21? This raises questions about the localization of DOC1-RFP to septa in Figure 8. No negative controls are included for this figure in the main text and are needed.

Response: We agree that autofluorescence can complicate the interpretation of RFP signals at septa. In response, we have added appropriate negative control to Figure 7, showing cells of the untagged wild type, which does not express any fluorescent marker protein. Additionally, we added supplemental figures showing additional pictures of the autofluorescent signal of the untagged wild type in comparison to the signal of SmDOC1-TagRFP-T fusion (S22+S23 Fig). These controls confirm that the RFP signal observed at septa in DOC1-RFP–expressing strains is specific and not due to autofluorescence.

Additionally, we have clarified in the Results and figure legend the potential contribution of autofluorescence observed in Figures S22 and S23, and we emphasize that the DOC1-RFP signal localizes above background autofluorescence. These revisions strengthen the interpretation that DOC1-RFP is genuinely localized to septa.

12. I would suggest moving Figure S17, to the main text in combination with Figure 7 as it is relevant to the hypothesis that the DOC proteins link the PR MAK kinase pathway and the STRIPAK complex.

Response: We agree that Figure S17 is directly relevant to the hypothesis that the DOC proteins link the PR MAP kinase pathway and the STRIPAK complex. In response, we have moved Figure S17 into the main text (now Fig. 5). The section has been revised to integrate the new figure placement. This change highlights the connection between DOC proteins, the PR MAP kinase pathway, and the STRIPAK complex more clearly.

13. The discussion is overly long and could be more carefully edited. For example, the paragraph starting on line 512 could be shortened and focused on data relevant to this study, not others.

Response: We thank the reviewer for this valuable suggestion. We agree that the Discussion was longer than necessary in some sections and could be more focused on the data presented in this study. In response, we have carefully revised the Discussion, including the paragraph starting on line 512, to focus on findings directly relevant to the role of DOC proteins in linking the PR MAP kinase pathway and the STRIPAK complex in *S. macrospora*. References to other systems have been reduced or removed where not essential, improving clarity and conciseness while maintaining appropriate context for our results.

14. What is meant by "sexual development" in the discussion could be made clearer. At what steps during sexual development do the authors believe that the SmDoc1/SmDoc2 is acting? This aspect could also be covered in the introduction—what are the steps of sexual development in S. macrospora and what is the role of STRIPAK and PR MAP kinase components in this pathway? Early development? Late development? For the non-fungal reader, a description of this process would be helpful—maybe a supplemental figure with the roles of STRIPAK and PR MAK kinase components?

Response: We agree that the term “sexual development” is broad and that a more detailed explanation of the specific stages affected by SmDoc1 and SmDoc2 would improve clarity, particularly for readers less familiar with fungal biology.

In response, we have revised the Introduction to include a concise overview of sexual development in *S. macrospora*, outlining key stages from vegetative growth, protoperithecia formation, fertilization, to mature perithecia. We have also summarized the known roles of the STRIPAK complex and PR MAP kinase components at these stages, indicating where their activities intersect with the DOC proteins.

In the Discussion, we now specify that SmDoc1 and SmDoc2 likely act during **early sexual development**, influencing processes such as cell fusion and initiation of protoperithecia formation, which are critical for subsequent perithecia formation.

15. I don't think figure 9 is needed, especially if the SmDoc1/SmDoc2 figures were combined (Figs. 5 ad 6).

Response: We agree that Figure 9 is largely redundant, particularly after combining the SmDoc1/SmDoc2 data into the revised Figures 4. Accordingly, we have removed Figure 9 from the manuscript and adjusted the text and figure references accordingly. This change streamlines the presentation and reduces redundancy, improving the overall flow of the Results section.

16. For Figure 10, some outputs could be included. For example, #1—steady state—is a biological process affected?? #2—assembly of PR signaling complex—what are the biological output(s)? #4 Disassembly—what biological aspect(s) is affected?

Response: We agree that Figure 10 could be clarified by specifying the biological outputs associated with each step. In response, we have revised the text of the discussion.

Suggested editorial/grammatical corrections

1. Line 123: we then investigated

Response: Was changed accordingly.

2. Line 124: homothallic species

Response: Was changed accordingly.

3. Line 215: delete before

Response: Was changed accordingly.

4. Line 271: unclear what is meant S4 Fig, S4.

Response: Was changed accordingly.

5. Line 365: spell out 5.

Response: Was changed accordingly.

6. Line 404: spell our 4

Response: Was changed accordingly.

7. Line 377: Not clear what is meant "Consistent with the proteinaceous environment"

Response: Removed during merging of Figures 5 and 6.

8. Line 466: data citation for this statement "The SmDOC1- and SmDOC2-BioID experiments enriched unique peptides of both, the shorter 447 MEK2-t1 and the novel MEK2-t2 isoform in the BioID eluates".

Response: We have added the appropriate data citation to support the statement. Specifically we refer to Supplement S8 data and S9 data which lists the unique peptides identified for MEK2 isoforms in the BioID experiments.

9. Line 583: These data

Response: Was changed accordingly.

10. Lines 648-650 should go into the results, not discussion. They would not need to be reiterated here.

Response: We agree that content of line 648-650 is more appropriate for the Result section. Accordingly, we have moved this text to the Results section, where relevant data is described and removed the redundant statement from the Discussion section.

------------------------------------

Reviewer #3:

This study by Hollstein et al. uses proximity labeling-based proteomics to map the protein interaction landscape of the STRIPAK signaling complex in the filamentous fungus Sordaria macrospora. Using TurboID labeling with the STRIPAK subunit SCI1 as bait, the authors uncover SmDOC2 as a prominent STRIPAK-associated factor and demonstrate that both SmDOC1 and SmDOC2 are functionally required for proper fruiting body formation. Genetic analyses reveal a striking non-additive phenotype: while single deletions of Smdoc1 or Smdoc2 cause near sterility, the double deletion restores wild-type sexual development, indicating mutually antagonistic roles. Proximity proteomics, yeast two-hybrid assays, and localization studies further show that SmDOC1/2 associate closely with components of the pheromone response MAPK pathway, including MEK2 and MAK2, and that SmDOC1 localizes to septal pore-associated ring structures. In addition, the authors demonstrated that doc1/2 mutants are also sterile in N. crassa. Together, the results establish the DOC1/2 system as a conserved regulatory module that integrates STRIPAK and MAPK signaling to fine-tune sexual development in both homothallic and heterothallic filamentous fungi.

Overall this is a very solid manuscript that contribute to our understanding about the STRIPAK signaling complex, the green bear genes Doc1/2 and their link to the MAPK signaling pathway. The manuscript provides lots of data about protein-protein interactions and binding, but unfortunately did not provide much mechanistic insight into how Smdoc1 or Smdoc2 causes sterility during sexual development. The authors show that Doc1/2 proteins localize to the septa-pore. How does this explain about the sterile phenotype of the single mutant and then the reversion of the fertile phenotype of the double mutants? How about the protein localization of the STRIPAK signaling complex and MAPk? Are they also there at the Septapore? Although the authors provide sufficient evidence to support protein-protein interactions, more experiments are definitely required to explain the molecular mechanisms, and regulation and function of Doc1/2 in Sordaria macrospora.

Response: We sincerely thank the reviewer for the thoughtful and constructive feedback. We appreciate the positive evaluation of our work and the acknowledgment of its contribution to understanding the STRIPAK signaling complex and the greenbeard genes SmDOC1/2 in *Sordaria macrospora*. We fully agree that, at present, our study provides stronger evidence for protein–protein interactions and spatial organization than for a complete mechanistic explanation of how SmDOC1 and SmDOC2 regulate fertility during sexual development. Our localization data show that SmDOC1 accumulates at septal pores.

At present, our data demonstrate physical and functional interactions between SmDOC1/2, STRIPAK components, and MAPK pathway proteins, but do not yet resolve their precise subcellular co-localization dynamics relative to the septal pore. We now explicitly state in the revised manuscript that future experiments addressing the subcellular localization of STRIPAK and MAPK components in relation to septal pores will be essential to fully define the signaling architecture. at the septal pore, a key regulatory structure for cytoplasmic continuity, signaling compartmentalization, and developmental coordination in filamentous fungi. While this model is consistent with the observed genetic and localization data, we acknowledge that it remains hypothetical at this stage. We have revised the Discussion section to clarify the conceptual model, explicitly acknowledge the current mechanistic limitations, and frame our findings as a foundation for future functional and spatially resolved studies. We emphasize that this work establishes SmDOC1/2 as novel regulatory components linking STRIPAK and MAPK signaling pathways to sexual development in *S. macrospora*, providing a molecular framework upon which future mechanistic dissection can be built. We believe this positioning accurately reflects the scope of the current study while highlighting its significance as a platform for further mechanistic investigation.
