# Supplementary material for: STRIPAK-associated greenbeard proteins DOC1 and DOC2 regulate MAK2 signaling and sexual development in Sordaria macrospora
Source: PLoS Biol. 2026 Aug 27;24(8):e3003969. doi: 10.1371/journal.pbio.3003969 (PMC13557503; doi:10.1371/journal.pbio.3003969)
Supplement: Peer review history

# Editor report: Decision Letter 0

**DOI:** 10.1371/journal.pbio.3003969.r001

**Authors:** Melissa Vazquez Hernandez

25 Nov 2025

Dear Dr Pöggeler,

Thank you for submitting your manuscript entitled "Capture of the SmSTRIPAK proxiome identifies the greenbeard proteins SmDOC1/2 as regulators of the MAK2 pathway to control sexual development in Sordaria macrospora" for consideration as a Research Article by PLOS Biology.

Your manuscript has now been evaluated by the PLOS Biology editorial staff as well as by an academic editor with relevant expertise and I am writing to let you know that we would like to send your submission out for external peer review.

However, before we can send your manuscript to reviewers, we need you to complete your submission by providing the metadata that is required for full assessment. To this end, please login to Editorial Manager where you will find the paper in the 'Submissions Needing Revisions' folder on your homepage. Please click 'Revise Submission' from the Action Links and complete all additional questions in the submission questionnaire.

Once your full submission is complete, your paper will undergo a series of checks in preparation for peer review. After your manuscript has passed the checks it will be sent out for review. To provide the metadata for your submission, please Login to Editorial Manager (https://www.editorialmanager.com/pbiology) within two working days, i.e. by Nov 27 2025 11:59PM.

If your manuscript has been previously peer-reviewed at another journal, PLOS Biology is willing to work with those reviews in order to avoid re-starting the process. Submission of the previous reviews is entirely optional and our ability to use them effectively will depend on the willingness of the previous journal to confirm the content of the reports and share the reviewer identities. Please note that we reserve the right to invite additional reviewers if we consider that additional/independent reviewers are needed, although we aim to avoid this as far as possible. In our experience, working with previous reviews does save time.

If you would like us to consider previous reviewer reports, please edit your cover letter to let us know and include the name of the journal where the work was previously considered and the manuscript ID it was given. In addition, please upload a response to the reviews as a 'Prior Peer Review' file type, which should include the reports in full and a point-by-point reply detailing how you have or plan to address the reviewers' concerns.

During the process of completing your manuscript submission, you will be invited to opt-in to posting your pre-review manuscript as a bioRxiv preprint. Visit http://journals.plos.org/plosbiology/s/preprints for full details. If you consent to posting your current manuscript as a preprint, please upload a single Preprint PDF.

Feel free to email us at plosbiology@plos.org if you have any queries relating to your submission.

Kind regards,

Melissa

Melissa Vazquez Hernandez, Ph.D.

Associate Editor

PLOS Biology

mvazquezhernandez@plos.org

# Peer review: Decision Letter 1

**DOI:** 10.1371/journal.pbio.3003969.r002

**Authors:** Melissa Vazquez Hernandez

7 Jan 2026

Dear Dr Pöggeler,

Happy New Year!

Thank you for your patience while your manuscript "Capture of the SmSTRIPAK proxiome identifies the greenbeard proteins SmDOC1/2 as regulators of the MAK2 pathway to control sexual development in Sordaria macrospora" was peer-reviewed at PLOS Biology. It has now been evaluated by the PLOS Biology editors, an Academic Editor with relevant expertise, and by three independent reviewers.

In light of the reviews, which you will find at the end of this email, we would like to invite you to revise the work to thoroughly address the reviewers' reports. As you will see below, majority of reviewers are positive about the relevance and novelty of the study, yet some concerns have raised during revision. Reviewer 1 is supportive but raises major concerns, emphasizing that while the identification of DOC proteins as links between STRIPAK and pheromone-response MAPK signaling is novel and well supported by proteomics and genetics, several conclusions, particularly regarding direct interactions, subcellular localization, and the proposed mechanistic model of PR complex disassembly, go beyond the data and should be softened, better quantified, or explicitly framed as speculative. Reviewer 2 highlights missing biological context (e.g., MAPK oscillation and cell fusion), insufficient justification for experimental choices, concerns about localization data due to autofluorescence and lack of negative controls, and the need to better define what stage(s) of sexual development are affected. Reviewer 3 considers the study solid and interesting but notes a major gap in mechanistic explanation, arguing that although the genetic and interaction data are convincing, the manuscript does not yet explain how DOC1/2 localization and antagonism translate into the observed sterility and rescue phenotypes, nor how STRIPAK and MAPK components are spatially and functionally coordinated. We agree with all reviewer concerns and would require some additional experimental revisions to address them, as we consider that this would strengthen the work.

Given the extent of revision needed, we cannot make a decision about publication until we have seen the revised manuscript and your response to the reviewers' comments. Your revised manuscript is likely to be sent for further evaluation by all or a subset of the reviewers.

In addition to these revisions, you will need to complete some formatting changes, which you will receive in a follow up email. A member of our team will be in touch with a set of requests shortly.

We expect to receive your revised manuscript within 4 months. Please email us (plosbiology@plos.org), or me, if you have any questions or concerns, or would like to request an extension.

At this stage, your manuscript remains formally under active consideration at our journal; please notify us by email if you do not intend to submit a revision so that we may withdraw it.

**IMPORTANT - SUBMITTING YOUR REVISION**

Your revisions should address the specific points made by each reviewer. Please submit the following files along with your revised manuscript:

1. A 'Response to Reviewers' file - this should detail your responses to the editorial requests, present a point-by-point response to all of the reviewers' comments, and indicate the changes made to the manuscript.

*NOTE: In your point-by-point response to the reviewers, please provide the full context of each review. Do not selectively quote paragraphs or sentences to reply to. The entire set of reviewer comments should be present in full and each specific point should be responded to individually, point by point.

You should also cite any additional relevant literature that has been published since the original submission and mention any additional citations in your response.

2. In addition to a clean copy of the manuscript, please also upload a 'track-changes' version of your manuscript that specifies the edits made. This should be uploaded as a "Revised Article with Changes Highlighted" file type.

*Re-submission Checklist*

When you are ready to resubmit your revised manuscript, please refer to this re-submission checklist: https://plos.io/Biology_Checklist

To submit a revised version of your manuscript, please go to https://www.editorialmanager.com/pbiology/ and log in as an Author. Click the link labelled 'Submissions Needing Revision' where you will find your submission record.

Please make sure to read the following important policies and guidelines while preparing your revision:

*Published Peer Review*

Please note while forming your response, if your article is accepted, you may have the opportunity to make the peer review history publicly available. The record will include editor decision letters (with reviews) and your responses to reviewer comments. If eligible, we will contact you to opt in or out. Please see here for more details:

https://blogs.plos.org/plos/2019/05/plos-journals-now-open-for-published-peer-review/

*PLOS Data Policy*

Please note that as a condition of publication PLOS' data policy (http://journals.plos.org/plosbiology/s/data-availability) requires that you make available all data used to draw the conclusions arrived at in your manuscript. If you have not already done so, you must include any data used in your manuscript either in appropriate repositories, within the body of the manuscript, or as supporting information (N.B. this includes any numerical values that were used to generate graphs, histograms etc.). For an example see here: http://www.plosbiology.org/article/info%3Adoi%2F10.1371%2Fjournal.pbio.1001908#s5

*Blot and Gel Data Policy*

We require the original, uncropped and minimally adjusted images supporting all blot and gel results reported in an article's figures or Supporting Information files. We will require these files before a manuscript can be accepted so please prepare them now, if you have not already uploaded them. Please carefully read our guidelines for how to prepare and upload this data: https://journals.plos.org/plosbiology/s/figures#loc-blot-and-gel-reporting-requirements

*Protocols deposition*

To enhance the reproducibility of your results, we recommend that if applicable you deposit your laboratory protocols in protocols.io, where a protocol can be assigned its own identifier (DOI) such that it can be cited independently in the future. Additionally, PLOS ONE offers an option for publishing peer-reviewed Lab Protocol articles, which describe protocols hosted on protocols.io. Read more information on sharing protocols at https://plos.org/protocols?utm_medium=editorial-email&utm_source=authorletters&utm_campaign=protocols

Thank you again for your submission to our journal. We hope that our editorial process has been constructive thus far, and we welcome your feedback at any time. Please don't hesitate to contact us if you have any questions or comments.

Sincerely,

Melissa

Melissa Vazquez Hernandez, Ph.D.

Associate Editor

PLOS Biology

mvazquezhernandez@plos.org

------------------------------------

REVIEWERS' COMMENTS

------------------------------------

Reviewer #1:

The manuscript presents a novel conceptual advance by identifying DOC proteins as shared regulatory components linking the STRIPAK phosphatase complex and the pheromone response (PR) MAPK signaling pathway. While DOC proteins were previously characterized in Neurospora crassa, their integration into STRIPAK-dependent signaling networks and their functional role in sexual development, particularly in a homothallic species, has not been previously reported. The integration of genetic, proteomic, and interaction-based data provides a framework for understanding how signaling specificity and pathway crosstalk are regulated in fungi, with implications for conserved eukaryotic signaling mechanisms. The experimental approaches employed (genetic analysis, BioID-based proximity labeling, mass spectrometry, and yeast two-hybrid assays) are appropriate and generally well executed. Proteomic analyses are well controlled, statistically robust, and supported by extensive supplementary data. However, several conclusions rely heavily on proximity labeling and descriptive phenotypes without sufficient functional or quantitative validation. In particular, the interpretations related to subcellular localization, direct protein-protein interactions (especially for SmDOC2), and the proposed mechanistic model of PR complex disassembly exceed what is directly supported by the data. No major new experimental approaches are strictly required for publication, but the paper would be substantially improved by: more cautious interpretation of BioID and localization data, clearer labeling of the speculative proposed models, improved figure and text consistency, and where feasible, quantification of key phenotypes.

In the introduction, the technical descriptions of the Turbo ID experiment are thorough and appreciated but may be shortened in the introduction, or fit in with the first results section or the methods. Given that the TurboID workflow has been previously established in this system and validated by the authors in their previous paper, it might be worth considering whether the schematic in Figure 1 could be streamlined or integrated with a subsequent figure, to focus directly on the biological results.

In figure 2B, the authors may wish to briefly note how the volcano plot presented relates to the volcano plot shown in the previous paper (Hollstein et al 2022), or whether the data were newly generated or reanalyzed using different statistical criteria.

For transparency, the legend of Fig S9 should indicate that the wild-type, Smdoc1, and Smdoc2 images shown are the same images used in Fig 3B.

In Fig 3 and Fig S9, the Smdoc1::DOC1 and Smdoc2::DOC2 complemented strains appear to exhibit lighter lid coloration than the wild-type. Is there a delay or defect in mature fruiting body formation? It is also worth noting that the double knockout mutant overall appears more similar to wild-type than either complemented strain, which perhaps could be due to ectopic integration of the complementation constructs. Many of the phenotypes in this section are presented descriptively. Incorporation of quantitative analyses for at least some assays would strengthen the robustness of the conclusions.

Several of the results in the text regarding the sexual development of N. crassa are not directly illustrated in Fig 4, which appears to only show self crosses. It would be helpful to clarify whether these reciprocal crosses and ascospore data are shown elsewhere, or if these are intended to function as representative images.

The results sections on MAK2 interactions and MEK2 analyses are supported primarily by supplemental tables and figures. A main text figure would help orient the reader to appreciate the relevance of DOC and MAP kinase signaling.

For the yeast two hybrid assay, given the parallel analysis of SmDOC1 and SmDOC2 in earlier sections, a brief explanation of whether SmDOC2 was also tested in the Y2H system is warranted. And if so, with what outcome.

Regarding the interpretation of the fluorescence localization data for SmDOC1/2 in Fig. 8: the SmDOC1-TagRFP signal is extremely weak and difficult to visually discern. Moreover, comparison with the control images in the Supplemental Figures is confusing: the panel labeled "wild type'" in S19 Fig appears to show a tagged strain, and untagged wild-type controls are not clearly presented for SmDOC1. For SmDOC2, the untagged wild type shows septal fluorescence patterns in both green and red channels that appear indistinguishable from those observed in tagged strains (S21 Fig), consistent with autofluorescence. As currently presented, the reader cannot assess whether the observed septal signal reflects specific DOC localization or background fluorescence.

In the Discussion, the genetic analyses support an important role for DOC proteins in sexual development, however, more explicitly qualifying species-specific conclusions (defects demonstrated in N. crassa) would better reflect the largely descriptive nature of the phenotypic data and avoid broader generalization to heterothallic fungi.

The section on SmDOC1 localization appears to overstate the strength of the fluorescence microscopy data. While Fig 8 is interpreted as showing ring-like localization at the septal pore, the signal is extremely weak and, as shown in the Supplemental Figures, similar septal fluorescence is observed in untagged wild-type controls, suggesting autofluorescence. Additionally, enrichment of septal-associated proteins in BioID reflects proximity rather than definitive spatial localization. Softening the language in this section and more explicitly acknowledging these limitations would better align with the presented data.

The statements describing SmDOC1/2 as "closely interacting" with PR signaling components appear stronger than what is directly supported by the data. Given that BioID reports proximity rather than physical interaction, and Y2H interactions are demonstrated only for SmDOC1 and not SmDOC2.

Fig 10 presents an interesting and coherent mechanistic model, however, the stepwise/directional nature of the diagram implies experimental validation that is not directly demonstrated in this study. In particular, reciprocal inhibition between SmDOC1 and SmDOC2, their proposed activation state, and their role in PR complex disassembly are inferred. I would suggest more explicitly labeling this model as speculative and adjusting both the figure and accompanying text to distinguish hypothesis from data-supported conclusions. Alternatively, the model could be framed as one of several possible mechanisms explaining the observed non-additive phenotypes, with reciprocal antagonism and PR complex disassembly presented as testable hypotheses.

------------------------------------

Reviewer #2:

Review of "Capture of the SmSTRIPAK proxiome identifies the greenbeard proteins SmDOC1/2 as regulators of the MAK2 pathway to control sexual development in Sordaria macrospora" by Hollstein et al., describes the identification of new proteins using biotin-labeling (Turbo-ID) that were associated with the STRIPAK complex in the filamentous fungus Sordaria macrospora. This reviewer appreciated the carefulness in data and controls for the Turbo-ID experiments. The new proteins identified had previously been characterized in the Neurospora crassa (Doc1 and Doc2) and regulate chemotropic interactions associated with cell fusion. The authors characterized deletion mutants of SmDoc1 and SmDoc2 and showed that they displayed sexual reproduction defects. The authors then used Turbo-ID to identify proteins that associated with SmDoc1 and SmDoc2 and a component of a pheromone response MAP kinase pathway. These results indicate an interaction between the STRIPAK complex and the pheromone response pathway via the Doc proteins to regulate sexual development in S. macrospora. This investigation is well written, with some suggested comments below. Major considerations are also listed below.

Major considerations

1.In the introduction, the regulation of the doc system in N. crassa of the oscillation of the components of the MAP kinase pathway during chemotrophic interactions needs to be described. The mention of this aspect in the discussion is not interpretable without this information. For example, in the discussion is the first time oscillation is mentioned "Multi-phosphorylated SmDOC1/2 might bind PR components more tightly without releasing them. This would result in a situation of competitive inhibition, which prevents the assembly of the MAPK signaling cascade and thereby suppresses oscillation".

2.Some of the introduction could be tightened up (some methods of the Turbo-ID could be moved to results).

3.Cell fusion is known to be important for the formation of reproductive structures in both S. macrospora and N. crassa; the DOC proteins regulate this process in N. crassa. I was surprised that this aspect of the SmDoc1 ad Smdoc2 mutants was not addressed in S. macrospora, as this could be the cause of the inability to form reproductive structures. In particular, the cell fusion mutants in N. crassa also show a delay in colony establishment.

4.An explanation of why the sci1 subunit of the SmSTRIPAK complex was used as bait is needed.

5.Are the doc-1 and doc-2 mutants from the N. crassa deletion collection or from the strains constructed by Heller et al.,? It is known that secondary mutations are common in the deletion strain collection, and these mutations are often in cell fusion genes; cell fusion mutants in N. crassa are defective/very delayed for protoperithecia formation. If from the N. crassa deletion collection, the phenotype of the doc-1/doc-2 mutants would need to be confirmed to be dependent on these mutations and not background mutations.

6.Fig. 1 (Turbo-ID method) could go into supplemental data.

7.There is an exceptionally large number of supplemental figures and tables. I suggest perhaps combining some of the excel spreadsheets on the Turbo-ID data with separate sheets in the same excel file and some of the figures could be combined (S5-S7, S10-S11, for example). The supplemental tables are not listed numerically in the manuscript (the first supplemental table mentioned is S6).

8.There are some grey dots that showed high significance in the volcano plots, but they are not labeled. Either indicate what these proteins are in the plot or in the legend.

9.Protoperithecia in the N. crassa doc-2 mutant not present in Fig. 4—perhaps a later photo showing formation of these structures?

10.Figures 5 and 6 could be combined.

11.Why was autofluorescence in the red and green channel to the septa identified in Figure S14 and S21? This raises questions about the localization of DOC1-RFP to septa in Figure 8. No negative controls are included for this figure in the main text and are needed.

12.I would suggest moving Figure S17, to the main text in combination with Figure 7 as it is relevant to the hypothesis that the DOC proteins link the PR MAK kinase pathway and the STRIPAK complex.

13.The discussion is overly long and could be more carefully edited. For example, the paragraph starting on line 512 could be shortened and focused on data relevant to this study, not others.

14.What is meant by "sexual development" in the discussion could be made clearer. At what steps during sexual development do the authors believe that the SmDoc1/SmDoc2 is acting? This aspect could also be covered in the introduction—what are the steps of sexual development in S. macrospora and what is the role of STRIPAK and PR MAP kinase components in this pathway? Early development? Late development? For the non-fungal reader, a description of this process would be helpful—maybe a supplemental figure with the roles of STRIPAK and PR MAK kinase components?

15.I don't think figure 9 is needed, especially if the SmDoc1/SmDoc2 figures were combined (Figs. 5 ad 6).

16.For Figure 10, some outputs could be included. For example, #1—steady state—is a biological process affected?? #2—assembly of PR signaling complex—what are the biological output(s)? #4 Disassembly—what biological aspect(s) is affected?

Suggested editorial/grammatical corrections

1.Line 123: we then investigated

2.Line 124: homothallic species

3.Line 215: delete before

4.Line 271: unclear what is meant S4 Fig, S4.

5.Line 365: spell out 5.

6.Line 404: spell our 4

7.Line 377: Not clear what is meant "Consistent with the proteinaceous environment"

8.Line 466: data citation for this statement "The SmDOC1- and SmDOC2-BioID experiments enriched unique peptides of both, the shorter 447 MEK2-t1 and the novel MEK2-t2 isoform in the BioID eluates".

9.Line 583: These data

10.Lines 648-650 should go into the results, not discussion. They would not need to be reiterated here.

------------------------------------

Reviewer #3:

This study by Hollstein et al. uses proximity labeling-based proteomics to map the protein interaction landscape of the STRIPAK signaling complex in the filamentous fungus Sordaria macrospora. Using TurboID labeling with the STRIPAK subunit SCI1 as bait, the authors uncover SmDOC2 as a prominent STRIPAK-associated factor and demonstrate that both SmDOC1 and SmDOC2 are functionally required for proper fruiting body formation. Genetic analyses reveal a striking non-additive phenotype: while single deletions of Smdoc1 or Smdoc2 cause near sterility, the double deletion restores wild-type sexual development, indicating mutually antagonistic roles. Proximity proteomics, yeast two-hybrid assays, and localization studies further show that SmDOC1/2 associate closely with components of the pheromone response MAPK pathway, including MEK2 and MAK2, and that SmDOC1 localizes to septal pore-associated ring structures. In addition, the authors demonstrated that doc1/2 mutants are also sterile in N. crassa. Together, the results establish the DOC1/2 system as a conserved regulatory module that integrates STRIPAK and MAPK signaling to fine-tune sexual development in both homothallic and heterothallic filamentous fungi.

Overall this is a very solid manuscript that contribute to our understanding about the STRIPAK signaling complex, the green bear genes Doc1/2 and their link to the MAPK signaling pathway. The manuscript provides lots of data about protein-protein interactions and binding, but unfortunately did not provide much mechanistic insight into how Smdoc1 or Smdoc2 causes sterility during sexual development. The authors show that Doc1/2 proteins localize to the septa-pore. How does this explain about the sterile phenotype of the single mutant and then the reversion of the fertile phenotype of the double mutants? How about the protein localization of the STRIPAK signaling complex and MAPk? Are they also there at the Septapore? Although the authors provide sufficient evidence to support protein-protein interactions, more experiments are definitely required to explain the molecular mechanisms, and regulation and function of Doc1/2 in Sordaria macrospora.

# Author comment: Author response to Decision Letter 2

**DOI:** 10.1371/journal.pbio.3003969.r003

4 Jun 2026

Attachment Submitted filename: 2026-06-01 Responses to the reviewers.docx

# Peer review: Decision Letter 2

**DOI:** 10.1371/journal.pbio.3003969.r004

**Authors:** Melissa Vazquez Hernandez

8 Jul 2026

Dear Stefanie,

Thank you for your patience while we considered your revised manuscript "Capture of the SmSTRIPAK proxiome identifies the greenbeard proteins SmDOC1/2 as regulators of the MAK2 pathway to control sexual development in Sordaria macrospora" for consideration as a Research Article at PLOS Biology. Your revised study has now been evaluated by the PLOS Biology editors, the Academic Editor and the original reviewers.

In light of the reviews, which you will find at the end of this email, we are pleased to offer you the opportunity to address the comments from the reviewers in a revision that we anticipate should not take you very long. We will then assess your revised manuscript and your response to the reviewers' comments with our Academic Editor aiming to avoid further rounds of peer-review, although we might need to consult with the reviewers, depending on the nature of the revisions.

As you will see in the reports, reviewers #2 and #3 are now broadly satisfied with the revised manuscript, and reviewer #1 also acknowledges the substantial improvements in clarity, balance, controls, and quantification. However, reviewer #1 remains concerned that key aspects of the proposed mechanistic model are still speculative and that the localization data do not yet provide sufficiently strong support for the conclusions drawn. In addition, reviewer #2 raises related concerns about the interpretation of SmDOC1 localization, particularly the description of a “ring-like” structure based on 2D images, and asks that the apparent septal-pore localization be discussed more directly in relation to its possible biological function in sexual development. After discussion with the Academic Editor, we are therefore inviting a light major revision focused on these remaining issues. While we will not require further experiments for mechanistic insights, we do ask that you strengthen and further qualify the SmDOC1 localization data, and ensure that any mechanistic conclusions are appropriately supported by the data or explicitly framed as speculative. Please also address reviewer #2’s remaining textual, citation, and clarification points.

In addition to these revisions, you may need to complete some formatting changes, which you will receive in a follow up email. A member of our team will be in touch with a set of requests shortly. If you do not receive a separate email within a few days, please assume that checks have been completed, and no additional changes are required.

We expect to receive your revised manuscript within 1 month. Please email us (plosbiology@plos.org) if you have any questions or concerns, or would like to request an extension.

At this stage, your manuscript remains formally under active consideration at our journal; please notify us by email if you do not intend to submit a revision so that we withdraw the manuscript.

**IMPORTANT - SUBMITTING YOUR REVISION**

Your revisions should address the specific points made by each reviewer. Please submit the following files along with your revised manuscript:

1. A 'Response to Reviewers' file - this should detail your responses to the editorial requests, present a point-by-point response to all of the reviewers' comments, and indicate the changes made to the manuscript.

*NOTE: In your point-by-point response to the reviewers, please provide the full context of each review. Do not selectively quote paragraphs or sentences to reply to. The entire set of reviewer comments should be present in full and each specific point should be responded to individually.

You should also cite any additional relevant literature that has been published since the original submission and mention any additional citations in your response.

2. In addition to a clean copy of the manuscript, please also upload a 'track-changes' version of your manuscript that specifies the edits made. This should be uploaded as a "Revised Article with Changes Highlighted " file type.

*Resubmission Checklist*

When you are ready to resubmit your revised manuscript, please refer to this resubmission checklist: https://plos.io/Biology_Checklist

To submit a revised version of your manuscript, please go to https://www.editorialmanager.com/pbiology/ and log in as an Author. Click the link labelled 'Submissions Needing Revision' where you will find your submission record.

Please make sure to read the following important policies and guidelines while preparing your revision:

*Published Peer Review*

Please note while forming your response, if your article is accepted, you may have the opportunity to make the peer review history publicly available. The record will include editor decision letters (with reviews) and your responses to reviewer comments. If eligible, we will contact you to opt in or out. Please see here for more details:

https://blogs.plos.org/plos/2019/05/plos-journals-now-open-for-published-peer-review/

*PLOS Data Policy*

Please note that as a condition of publication PLOS' data policy (http://journals.plos.org/plosbiology/s/data-availability) requires that you make available all data used to draw the conclusions arrived at in your manuscript. If you have not already done so, you must include any data used in your manuscript either in appropriate repositories, within the body of the manuscript, or as supporting information (N.B. this includes any numerical values that were used to generate graphs, histograms etc.). For an example see here: http://www.plosbiology.org/article/info%3Adoi%2F10.1371%2Fjournal.pbio.1001908#s5

*Blot and Gel Data Policy*

We require the original, uncropped and minimally adjusted images supporting all blot and gel results reported in an article's figures or Supporting Information files. We will require these files before a manuscript can be accepted so please prepare them now, if you have not already uploaded them. Please carefully read our guidelines for how to prepare and upload this data: https://journals.plos.org/plosbiology/s/figures#loc-blot-and-gel-reporting-requirements

*Protocols deposition*

To enhance the reproducibility of your results, we recommend that if applicable you deposit your laboratory protocols in protocols.io, where a protocol can be assigned its own identifier (DOI) such that it can be cited independently in the future. Additionally, PLOS ONE offers an option for publishing peer-reviewed Lab Protocol articles, which describe protocols hosted on protocols.io. Read more information on sharing protocols at https://plos.org/protocols?utm_medium=editorial-email&utm_source=authorletters&utm_campaign=protocols

Thank you again for your submission to our journal. We hope that our editorial process has been constructive thus far, and we welcome your feedback at any time. Please don't hesitate to contact us if you have any questions or comments.

Sincerely,

Melissa

Melissa Vazquez Hernandez, Ph.D.

Senior Editor

PLOS Biology

mvazquezhernandez@plos.org

----------------------------------------------------------------

REVIEWERS' COMMENTS:

----------------------------------------------------------------

Reviewer #1:

I appreciate the effort the authors put into revising the manuscript. They have addressed the specific concerns raised by the reviewers, added important controls and quantification, clarified several aspects of the study, and generally made the manuscript more balanced and easier to follow. I also appreciate that the authors have substantially tempered a number of their conclusions and now more clearly distinguish between observations supported by the data and the more speculative aspects of the proposed model.

With that said, my overall assessment regarding the suitability for PLOS Biology is unchanged. While the revised manuscript presents an interesting set of genetic, proteomic, and interaction data linking DOC proteins, STRIPAK, and the pheromone response MAPK pathway, the central mechanistic model is still largely speculative. The revisions mainly improve interpretation of the existing data rather than providing new evidence that directly establishes how these components function together. Although the authors now appropriately acknowledge that key aspects of the proposed model remain hypothetical and are supported largely by proximity and genetic data, the revision does not substantially advance the mechanistic understanding of how DOC1/2 regulate MAPK signaling or explain the suppression phenotype observed in the double mutant.

I also remain unconvinced by the localization data. The authors appropriately softened their interpretation, but the signal is weak and difficult to separate from background fluorescence, limiting the conclusions that can be drawn from these experiments. I believe the study presents a valuable dataset and a useful framework for future investigation, but in its current form it remains largely descriptive and hypothesis-generating rather than mechanistic. For this reason, while I consider the work scientifically sound and potentially suitable for a strong journal such as PLOS Genetics, I do not feel that it provides the level of mechanistic insight expected for publication in PLOS Biology.

----------------------------------------------------------------

Reviewer #2:

This reviewer appreciates the thoughtful responses by the authors to reviewer's questions and suggestions and the thorough revision of the manuscript. My comments below are minor, with a couple of important questions that can be addressed without additional experimentation.

Line 137: hardly to hard.

Line 147: reference for ccg-1 and use as a promoter.

Line 279: reference for the trpC promoter.

Line 309: reference for anthranilate synthase terminator in A. nidulans.

Line 376: spell out Streptomyces first time it is used.

Line 428: Not clear why this control sample data was "interesting".

Line 467, plus other sections of the manuscript (Fig. 7 legend; line 583) where localization of SmDOC1 is discussed. Localization of SmDOC1 to a "ring-like structure" is not apparent from the localization data presented, as the images are in 2D.

Line 510: define "SIN" and its function.

Line 519: delete "the".

Line 527 "While these data…"

Line 570: during vegetative growth....

Line 582-595: I was surprised that the localization of SmDOC1 to the septal pore (and specifically to the pore, not the entire septum), was not discussed in relation to its biological function in sexual development. This aspect needs some discussion, not just a list of localization of other proteins to septa in this paragraph.

----------------------------------------------------------------

Reviewer #3:

The authors have significantly improved the clarity and rigor of the manuscript and sufficiently addressed the concerns and comments raised by the reviewers.

# Author comment: Author response to Decision Letter 3

**DOI:** 10.1371/journal.pbio.3003969.r005

17 Jul 2026

Attachment Submitted filename: Response to the reviewers.pdf

# Editor report: Decision Letter 3

**DOI:** 10.1371/journal.pbio.3003969.r006

**Authors:** Melissa Vazquez Hernandez

23 Jul 2026

Dear Dr Pöggeler,

Thank you for your patience while we considered your revised manuscript "Capture of the SmSTRIPAK proxiome identifies the greenbeard proteins SmDOC1/2 as regulators of the MAK2 pathway to control sexual development in Sordaria macrospora" for publication as a Research Article at PLOS Biology. This revised version of your manuscript has been evaluated by the PLOS Biology editors, and the Academic Editor.

Based on our Academic Editor's assessment of your revision, we are likely to accept this manuscript for publication, provided you satisfactorily address the following data and other policy-related requests.

1) We routinely suggest changes to titles to ensure maximum accessibility for a broad, non-specialist readership, and to ensure they reflect the contents of the paper. Please ensure you change both the manuscript file and the online submission system, as they need to match for final acceptance:

"STRIPAK-associated greenbeard proteins DOC1 and DOC2 regulate MAK2 signaling and sexual development in Sordaria macrospora"

2) Please add weblink of the funding agencies in the Financial Disclosure statement in the manuscript details.

3) We do not have a word limit. Please move the methods and references present in the supplements to the main text which can provide the readers an easier access to all information.

4) You may be aware of the PLOS Data Policy, which requires that all data be made available without restriction: http://journals.plos.org/plosbiology/s/data-availability. For more information, please also see this editorial: http://dx.doi.org/10.1371/journal.pbio.1001797

Please supply the numerical values either in the a supplementary file or as a permanent DOI’d deposition for the following figures:

Figure 1B, 4B, 5, S5, S10AB, S13

NOTE: the numerical data provided should include all replicates AND the way in which the plotted mean and errors were derived (it should not present only the mean/average values).

5) Please cite the location of the data clearly in all relevant main and supplementary Figure legends, e.g. “The data underlying this Figure can be found in S1 Data” or “The data underlying this Figure can be found in https://doi.org/10.5281/zenodo.XXXXX”

6) Please ensure that you are using best practice for statistical reporting and data presentation. These are our guidelines https://journals.plos.org/plosbiology/s/best-practices-in-research-reporting#loc-statistical-reporting and a useful resource on data presentation https://journals.plos.org/plosbiology/article?id=10.1371/journal.pbio.1002128

7) If you are reporting experiments where n ≤ 5, please plot each individual data point.

8) We require the original, uncropped and minimally adjusted images supporting all blot and gel results reported in the Figures S3ABC, S6AB, S7AB, S8AB, S14AB, S15AB, S19, S24ABC

-- We will require these files before a manuscript can be accepted so please prepare and upload them now. Please carefully read our guidelines for how to prepare and upload this data: https://journals.plos.org/plosbiology/s/figures#loc-blot-and-gel-reporting-requirements

9) Supplementary files (e.g., excel). Please ensure that all data files are uploaded as 'Supporting Information' and are invariably referred to (in the manuscript, figure legends, and the Description field when uploading your files) using the following format verbatim: S1 Data, S2 Data, etc. Multiple panels of a single or even several figures can be included as multiple sheets in one excel file that is saved using exactly the following convention: S1_Data.xlsx (using an underscore).

10) Please ensure that your Data Statement in the submission system accurately describes where your data can be found and is in final format, as it will be published as written there

11) Per journal policy, if you have generated any custom code during the course of this investigation, please make it available without restrictions. Please ensure that the code is sufficiently well documented and reusable, and that your Data Statement in the Editorial Manager submission system accurately describes where your code can be found. More information on our Code Policy, what and how to share can be found here: https://journals.plos.org/plosbiology/s/code-availability

Please note that we cannot accept sole deposition of code in GitHub, as this could be changed after publication. However, you can archive this version of your publicly available GitHub code to Zenodo. Once you do this, it will generate a DOI number, which you will need to provide in the Data Accessibility Statement (you are welcome to also provide the GitHub access information). See the process for doing this here: https://docs.github.com/en/repositories/archiving-a-github-repository/referencing-and-citing-content

As you address these items, please take this last chance to review your reference list to ensure that it is complete and correct. If you have cited papers that have been retracted, please include the rationale for doing so in the manuscript text, or remove these references and replace them with relevant current references. Any changes to the reference list should be mentioned in the cover letter that accompanies your revised manuscript.

In addition to these revisions, you may need to complete some formatting changes, which you will receive in a follow up email. A member of our team will be in touch with a set of requests shortly. If you do not receive a separate email within a few days, please assume that checks have been completed, and no additional changes are required.

We expect to receive your revised manuscript within two weeks.

To submit your revision, please go to https://www.editorialmanager.com/pbiology/ and log in as an Author. Click the link labelled 'Submissions Needing Revision' to find your submission record. Your revised submission must include the following:

- a cover letter that should detail your responses to any editorial requests, if applicable, and whether changes have been made to the reference list

- a Response to Reviewers file that provides a detailed response to the reviewers' comments (if applicable, if not applicable please do not delete your existing 'Response to Reviewers' file.)

- a track-changes file indicating any changes that you have made to the manuscript.

NOTE: If Supporting Information files are included with your article, note that these are not copyedited and will be published as they are submitted. Please ensure that these files are legible and of high quality (at least 300 dpi) in an easily accessible file format. For this reason, please be aware that any references listed in an SI file will not be indexed. For more information, see our Supporting Information guidelines:

https://journals.plos.org/plosbiology/s/supporting-information

*Published Peer Review History*

Please note that you may have the opportunity to make the peer review history publicly available. The record will include editor decision letters (with reviews) and your responses to reviewer comments. If eligible, we will contact you to opt in or out. Please see here for more details:

https://plos.org/published-peer-review-history/

*Press*

Should you, your institution's press office or the journal office choose to press release your paper, please ensure you have opted out of Early Article Posting on the submission form. We ask that you notify us as soon as possible if you or your institution is planning to press release the article.

*Protocols deposition*

To enhance the reproducibility of your results, we recommend that if applicable you deposit your laboratory protocols in protocols.io, where a protocol can be assigned its own identifier (DOI) such that it can be cited independently in the future. Additionally, PLOS ONE offers an option for publishing peer-reviewed Lab Protocol articles, which describe protocols hosted on protocols.io. Read more information on sharing protocols at https://plos.org/protocols?utm_medium=editorial-email&utm_source=authorletters&utm_campaign=protocols

Please do not hesitate to contact me should you have any questions.

Sincerely,

Melissa

Melissa Vazquez Hernandez, Ph.D.

Senior Editor

mvazquezhernandez@plos.org

PLOS Biology

# Author comment: Author response to Decision Letter 4

**DOI:** 10.1371/journal.pbio.3003969.r007

6 Aug 2026

Attachment Submitted filename: Response_to_the_reviewers_auresp_4.pdf

# Editor report: Decision Letter 4

**DOI:** 10.1371/journal.pbio.3003969.r008

**Authors:** Melissa Vazquez Hernandez

12 Aug 2026

Dear Dr Pöggeler,

Thank you for the submission of your revised Research Article "STRIPAK-associated greenbeard proteins DOC1 and DOC2 regulate MAK2 signaling and sexual development in Sordaria macrospora " for publication in PLOS Biology. On behalf of my colleagues and the Academic Editor, Joseph Heitman, I am pleased to say that we can in principle accept your manuscript for publication, provided you address any remaining formatting and reporting issues. These will be detailed in an email you should receive within 2-3 business days from our colleagues in the journal operations team; no action is required from you until then. Please note that we will not be able to formally accept your manuscript and schedule it for publication until you have completed any requested changes.

Please take a minute to log into Editorial Manager at http://www.editorialmanager.com/pbiology/, click the "Update My Information" link at the top of the page, and update your user information to ensure an efficient production process.

PRESS

We frequently collaborate with press offices. If your institution or institutions have a press office, please notify them about your upcoming paper at this point, to enable them to help maximise its impact. If the press office is planning to promote your findings, we would be grateful if they could coordinate with biologypress@plos.org. If you have previously opted in to the early version process, we ask that you notify us immediately of any press plans so that we may opt out on your behalf.

We also ask that you take this opportunity to read our Embargo Policy regarding the discussion, promotion and media coverage of work that is yet to be published by PLOS. As your manuscript is not yet published, it is bound by the conditions of our Embargo Policy. Please be aware that this policy is in place both to ensure that any press coverage of your article is fully substantiated and to provide a direct link between such coverage and the published work. For full details of our Embargo Policy, please visit http://www.plos.org/about/media-inquiries/embargo-policy/.

Thank you again for choosing PLOS Biology for publication and supporting Open Access publishing. We look forward to publishing your study.

Sincerely,

Melissa

Melissa Vazquez Hernandez, Ph.D., Ph.D.

Senior Editor

PLOS Biology

mvazquezhernandez@plos.org
